# Investigation on the reactivity of α-azidochalcones with carboxylic acids: Formation of α-amido-1,3-diketones and highly substituted 2-(trifluoromethyl)oxazoles

**DOI:** 10.3762/bjoc.11.219

**Published:** 2015-10-29

**Authors:** Kandasamy Rajaguru, Arumugam Mariappan, Rajendran Suresh, Periasamy Manivannan, Shanmugam Muthusubramanian

**Affiliations:** 1 Department of Organic Chemistry, School of Chemistry, Madurai Kamaraj University, Madurai, 625 021, India; 2 Syngene International Limited, Biocon, Bangalore, 560 100, India

**Keywords:** α-amido-1,3-diketones, α-azidochalcones, carboxylic acids, 2*H*-azirines, oxazoles

## Abstract

The reaction of α-azidochalcones with carboxylic acids has been investigated resulting in the formation of α-amido-1,3-diketones under microwave irradiation via in situ formation of 2*H*-azirine intermediates. An interesting reaction is described wherein, with trifluoroacetic acid at lower temperature, it affords highly substituted 2-(trifluoromethyl)oxazoles. These flexible transformations proceed under solvent free conditions in good to excellent yields without any catalyst.

## Introduction

**Figure 1:**
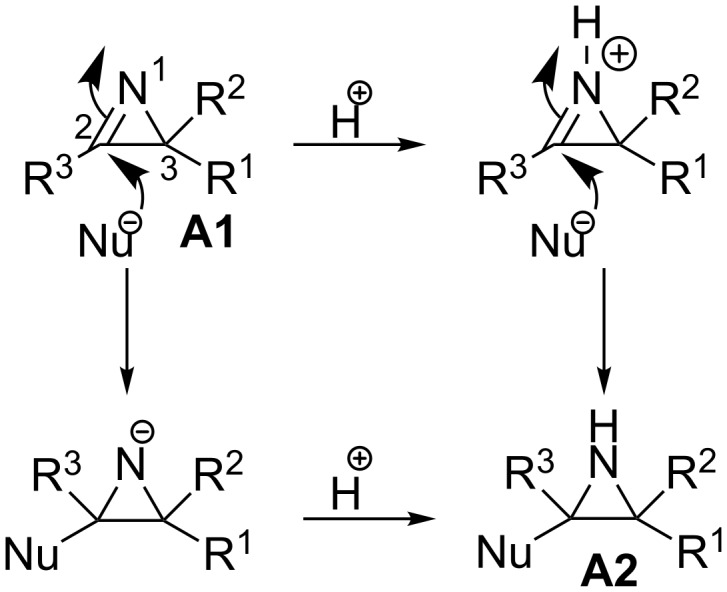
Formation of substituted aziridine.

**Figure 2:**
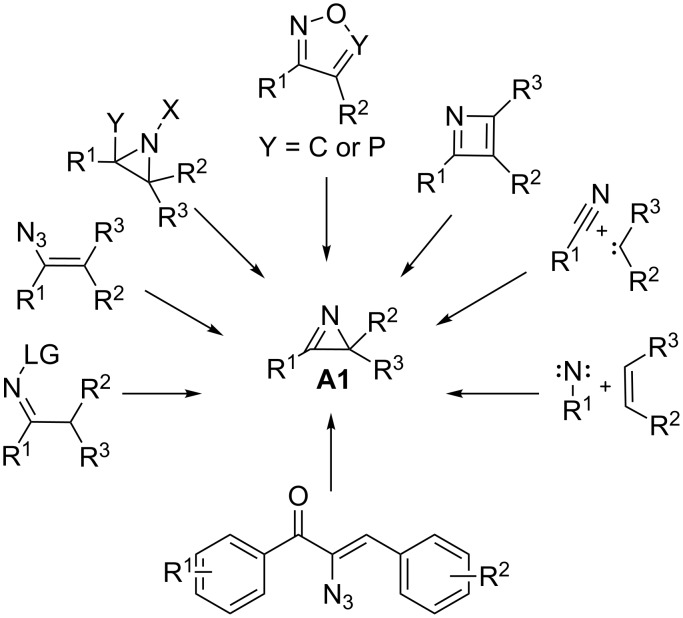
Various strategies for the formation of 2*H*-azirine.

α-Azidochalcones are one of the most attractive three-atom synthons for the formation of nitrogen-containing organic motifs. Due to their versatile reactivity, they have attracted considerable attention since last decade [1–6]. In drug discovery, secondary amides are an important class of compounds. The *N*-(2-keto)amide skeleton is of particular interest, as it serves as a synthetic precursor for various small heterocyclic compounds such as imidazoles, oxazoles and thiazoles [7–9]. Azirine, the smallest nitrogen-containing unsaturated three-membered heterocyclic system, is well known as a reactive intermediate in several synthetic transformations [10–15]. The chemistry of 2*H*-azirines has been extensively explored because of its strained molecular structure, unique reactivity and synthetic applications [10–22]. α-Substituted 2*H*-azirines are impressive intermediates for the synthesis of various substituted heterocyclic compounds [23–25]. Nucleophiles can interact with the electrophilic carbon of the strained 2*H*-azirine rings and due to the ring strain in the three-membered 2*H*-azirine ring system **A1**, the electrophilic character of the C(2)–N double bond is higher than a normal imine (Figure 1) [10,26–27]. The generated substituted aziridines **A2** may undergo further ring opening reactions. The α-azidochalcones **1** are suitable as precursors for the generation of 2*H*-azirines **A1**, though several other methods are available (Figure 2) [10,24].

In this article, we intend to demonstrate the reactivity of 2*H*-azirine **A1** towards carboxylic acids [28]. α-Azidochalcones have been chosen to generate 2*H*-azirines via vinyl nitrene intermediates. α-Azidochalcones can be synthesized from the corresponding benzylidene acetophenones in two steps following a literature procedure [29].

## Results and Discussion

**Scheme 1:**
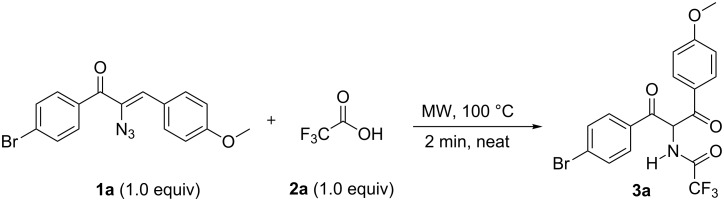
Attempted reaction for the synthesis of **3a**.

The investigation was initiated by treating α-azidochalcone **1a** (R^1^ = 4-Br, R^2^ = 4-OMe) with trifluoroacetic acid (**2a**). This acid was allowed to react with the azidochalcone at room temperature for 30 minutes and a low yield of **3a** (34%) was noticed with undesirable side products. Due to operational simplicity and efficiency, microwave irradiation in organic synthesis has become more popular as an environmental friendly way [30–32]. Thus, to increase the product yield, the investigation was continued by treatment of α-azidochalcone **1a** (R^1^ = 4-Br, R^2^ = 4-OMe) with trifluoroacetic acid under microwave conditions [33] at 100 °C for 2 minutes to obtain diketone **3a** (79%) as a solid without any side products (Scheme 1).

The ^1^H NMR spectrum of **3a** exhibits a methine proton doublet at 6.78 ppm. In the ^13^C NMR spectrum, two carbonyl carbon signals and an amide carbon signal appear at 190.6, 188.8, and 165.1 ppm, respectively. In addition, a methine carbon signal appears at 60.4 ppm. The structural confirmation of α-amido-1,3-diketone **3e** by one- and two-dimensional NMR spectroscopic data is depicted (see Supporting Information File 1).

**Figure 3:**
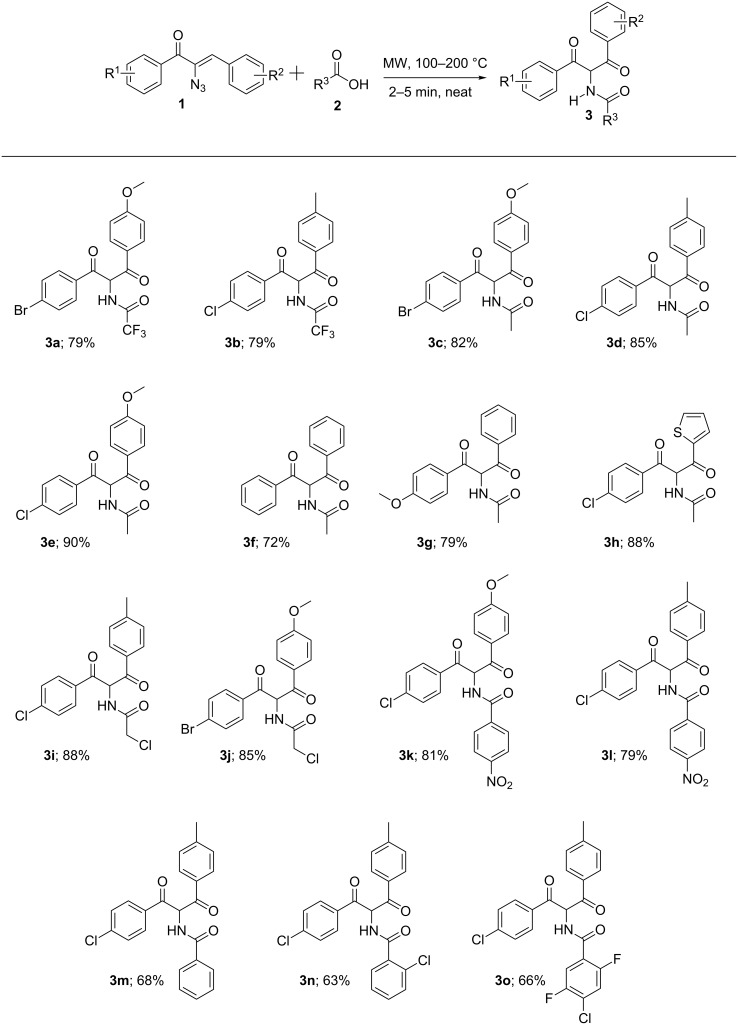
Synthesis of α-amido-1,3-diketone (**3a–o**). Reaction conditions: α-azidochalcone **1** (1.0 equiv) and carboxylic acid **2** (1.0 equiv), 100–200 °C, 2–5 min.

With this optimised protocol, we subsequently extended the scope of this transformation with other carboxylic acids as well. The reaction works well with different acids including acetic acid (**2b**) (**3c**–**h**) and chloroacetic acid (**2c**) (**3i** and **3j**). It should be mentioned that the reaction goes well with various substituted benzoic acids **2d–g** as well and the resultant α-amido-1,3-diketones **3k**–**o** are obtained in moderate to good yields (Figure 3).

**Scheme 2:**
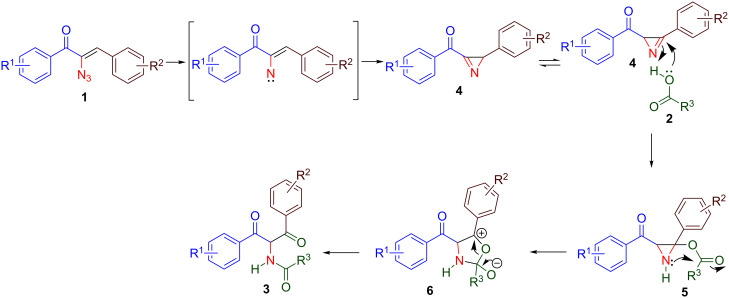
Plausible mechanism.

The mechanism for the formation of α-amido-1,3-diketone **3** is given in Scheme 2. Initially, by thermolysis, α-azidochalcone undergoes denitrogenative decomposition to form a cyclic imine – a highly strained three-membered 2*H*-azirine, **4**. Subsequent attack by the acid results in the aziridine adduct **5**, which then undergoes intramolecular nucleophilic addition on the carboxylic group with the nitrogen lone pair, ultimately yielding α-amido-1,3-diketone **3** by ring opening of **6** (Scheme 2).

**Scheme 3:**
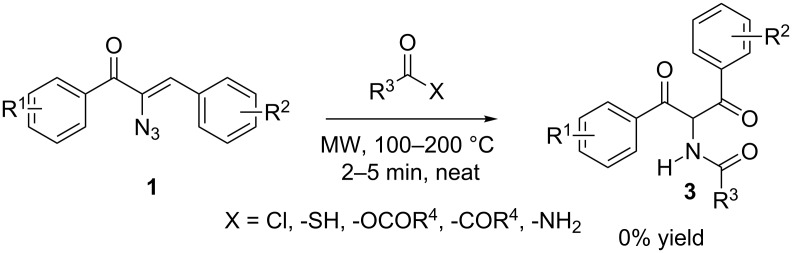
Attempted reaction with acid derivatives.

Having established the formation of **3** from azidochalcones with carboxylic acids under microwave conditions, the efficiency of this conversion was checked by replacing the carboxylic acids with equivalent systems such as acid chlorides, thioacetic acids, anhydrides, esters and amides under the same conditions. However, the expected products were not formed, emphasising the need for the hydroxy group in product formation (Scheme 3).

**Scheme 4:**
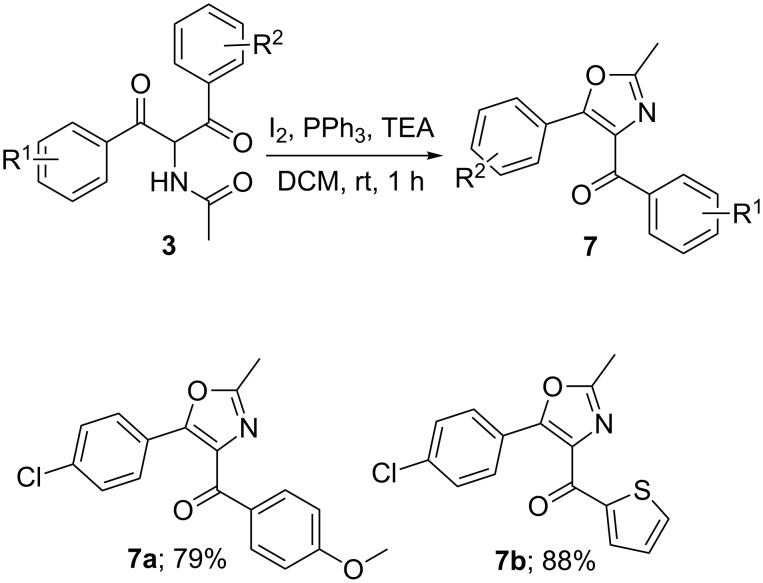
Oxazole formation from **3**.

Compounds **3** have 1,4-dicarbonyl functionality and are capable undergoing further useful transformations yielding different heterocyclic systems. This is illustrated with one reaction in which **3** was subjected to treatment with triphenylphosphine in the presence of iodine and triethylamine in dry dichloromethane at room temperature (Scheme 4) [34–35]. As expected, compounds **3** are converted to 2,4,5-substituted oxazole **7** regiospecifically in good to excellent yield.

**Figure 4:**
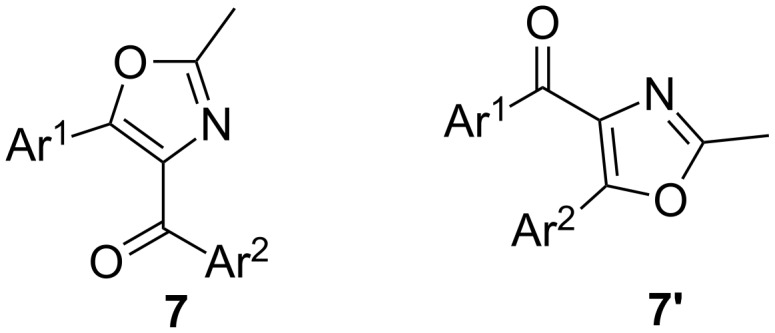
Possible isomers for **7**.

Compounds **7a** and **7b** have been characterized by one and two-dimensional NMR spectroscopy (see Supporting Information File 1). It can be noticed that the cyclisation could have happened in two different ways yielding either **7** or **7'** (Figure 4). The fact that the compound formed is **7** and not **7'** is confirmed by the HMBC spectrum of **7a**. The carbonyl carbon at 187.1 ppm has a HMBC contour with the hydrogens at 8.11 ppm, which have been shown to be *meta* to methoxy not *meta* to chloro by an H,H-COSY experiment.

**Scheme 5:**
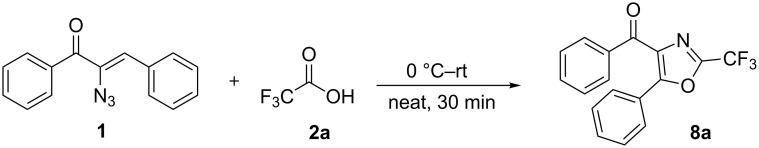
Oxazole formation.

When the α-azidochalcone **1** (R^1^ = H, R^2^ = H) was treated with trifluoroacetic acid at 0 °C and allowed to warm to ambient temperature for 30 min, **8a** was obtained (Scheme 5) [36].

**Figure 5:**
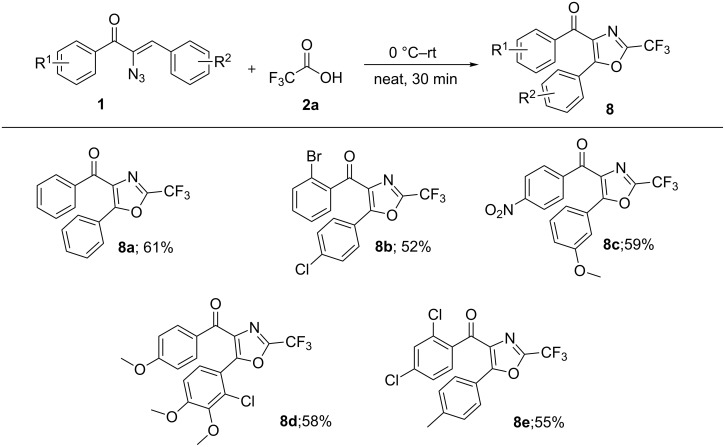
Synthesis of highly substituted 2-(trifluoromethyl)oxazoles (**8a–e**). Reaction conditions: α-azidochalcone **1** (1.0 equiv) and trifluoro acetic acid **2a** (2.0 equiv), 0 °C–rt, 30 min.

In ^1^H NMR spectrum of **8a**, there is no methine proton signal as in the case of **3** and in the ^13^C NMR spectrum, only one carbonyl carbon signal was observed at 187.3 ppm with no amide carbonyl carbon. The investigation of the structure of **8a** indicated that the compound is a highly substituted 2-(trifluoromethyl)oxazole. With different α-azidochalcones, differently substituted **8** have been isolated (Figure 5).

**Scheme 6:**
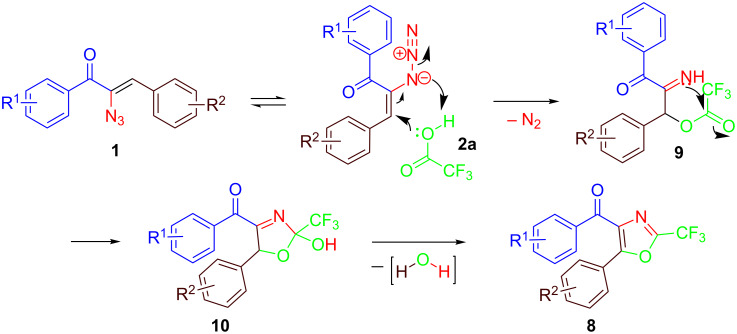
Mechanism for the formation of **8**.

It should be mentioned that oxazole formation is observed only at low temperature with trifluoroacetic acid and not with other carboxylic acids. A plausible mechanism has been illustrated for the formation of substituted oxazoles (Scheme 6). Initial attack of trifluoroacetic acid on the azide via Michael addition affords active intermediate **9**. Subsequently, an intramolecular nucleophilic addition takes place to form intermediate **10**. This is followed by the elimination of water leading to **8**. The reaction goes on the azide directly at low temperature, while at elevated temperature, it proceeds through azirine intermediates.

## Conclusion

In conclusion, the present work describes a new strategy for the synthesis of α-amido-1,3-diketones and highly substituted 2-(trifluoromethyl)oxazoles from α-azidochalcones and commercially available carboxylic acids without any catalyst in high yields. The entire sequence is realized under mild and simple conditions. This reaction is synthetically useful for the construction of other heterocyclic systems.

## Experimental

### General

A CEM Discover microwave synthesizer (Model No: 908010) operating at 180/264 V and 50/60 Hz with microwave power maximum level of 300 W and microwave frequency of 2455 MHz was employed for the microwave-assisted experiments. Nuclear Magnetic Resonance (^1^H, ^13^C two-dimensional COSY NMR) spectra were recorded on 300 MHz or 400 MHz spectrometers (Bruker) in CDCl_3_ and DMSO-*d*_6_ using TMS as an internal standard. Chemical shifts are reported in parts per million (δ), coupling constants (*J* values) are reported in Hertz (Hz) and spin multiplicities are indicated by the following symbols: s (singlet), d (doublet), t (triplet), m (multiplet). ^13^C NMR spectra were routinely run with broadband decoupling. Pre coated silica gel on aluminium plates (Merck) were used for TLC analysis with a mixture of petroleum ether (60–80 °C) and ethyl acetate as the eluent. Electrospray ionization (ESI) mass spectra were obtained on an LCQ Fleet mass spectrometer, Thermo Fisher Instruments Limited, US and an Agilent mass spectrometer. Infrared spectra were recorded on a Shimadzu FTIR instrument (KBr pellet). Elemental analyses were performed on a Perkin Elmer 2400 Series II Elemental CHNS analyser. The NH signal, being very broad, is not noticed in many ^1^H NMR spectra.

### General procedure

**Synthesis of 3:** A mixture of α-azidochalcone **1** (1.0 equiv) and carboxylic acid **2** (1.0 equiv or slight excess, if liquid acid was employed) was taken in a 10 mL quartz vial and placed in the microwave oven. The vial was sealed with a pressure cap and subjected to microwave irradiation. The irradiation was programmed between 100–200 °C, 120 W, 5 bar, for 5 min depending on the boiling point/melting point of the respective carboxylic acids. The reaction was monitored by TLC using petroleum ether/ethyl acetate mixture (7:3) as the eluent. After the reaction was cooled to room temperature ice-cold water was added. The precipitate obtained was filtered, dried in vacuum and recrystallized from ethanol to afford **3**.

**Representative scale-up example for the synthesis of 3e using the 80 mL vessel**: In an 80 mL glass vessel was placed α-azidochalcone **1** (R^1^ = 4-Cl, R^2^ = 4-OMe, 2.0 g, 1.0 equiv), acetic acid **2b** (0.4 mL, 1.0 equiv slight excess). The vessel was sealed with a pressure cap and placed into the microwave cavity. The irradiation was programmed at 100 °C, 120 W, 5 bar, for 5 min. After allowing the reaction mixture to cool to room temperature, ice-cold water was added. The precipitate obtained was filtered, dried in vacuum and recrystallized from ethanol to afford **3e** (yield: 1.93 g; 88%).

**Synthesis of 7:** To a stirred solution of triphenylphosphine (2.0 equiv) and iodine in dichloromethane, triethylamine was added slowly. After 10 minutes a solution of α-amido-1,3-diketone **3** (1.0 equiv) in dichloromethane (5 mL) was added dropwise. The completion of reaction was monitored by TLC employing petroleum ether/ethyl acetate mixture (8:2) as the eluent. After completion of the reaction, the solvent was removed in vacuo, and the residue was directly purified by column chromatography on silica gel (hexane/ethyl acetate 9:1) to yield oxazole **7**.

**Synthesis of 8:** In a dry two neck round bottom reaction vessel equipped with a calcium guard tube and a magnetic bar, α-azidochalcone (1.0 equiv) was taken and trifluoro acetic acid (2.0 equiv) was added to the reaction vessel dropwise at 0 °C. Then, the temperature of the reaction mixture was slowly raised to room temperature in 30 min. The reaction was monitored by TLC using petroleum ether/ethyl acetate mixture (4:1) as the eluent. After completion of the reaction, the mass was treated with ice cold saturated solution of sodium bicarbonate and extracted with ethyl acetate (2 × 10 mL). The combined organic layers were dried with anhydrous Na_2_SO_4_ and concentrated in vacuo to afford oxazole **8**.

**Representative scale-up example for the synthesis of 8a:** In a 25 mL dry two neck round bottom reaction vessel equipped with a calcium guard tube and a magnetic bar, α-azidochalcone **1** (R^1^ = H, R^2^ = H, 2.0 g, 1.0 equiv) was taken and trifluoroacetic acid (**2a**, 1.2 mL, 2.0 equiv) was added to the reaction vessel dropwise at 0 °C. The temperature of the reaction mixture was slowly raised to room temperature in 30 min. The reaction was monitored by TLC using petroleum ether/ethyl acetate mixture (8:2) as the eluent. After completion of the reaction, the resultant mass was treated with an ice-cold saturated solution of sodium bicarbonate and extracted with ethyl acetate (3 × 10 mL). The combined organic layers were dried over anhydrous Na_2_SO_4_ and concentrated in vacuo to afford oxazole **8a** (yield: 1.52 g; 60%).

## Supporting Information

File 1 Characterization data of new compounds **3**, **7** and **8** and copies of ^1^H ^13^C, two-dimentional NMR and ESI mass spectra.
