# Supplementary material for: Investigation on the reactivity of α-azidochalcones with carboxylic acids: Formation of α-amido-1,3-diketones and highly substituted 2-(trifluoromethyl)oxazoles
Source: Beilstein J Org Chem. 2015 Oct 29;11:2021–8. doi: 10.3762/bjoc.11.219 (PMC4661007; doi:10.3762/bjoc.11.219)
Supplement: File 1 — Characterization data of new compounds 3, 7 and 8 and copies of 1H 13C, two-dimentional NMR and ESI mass spectra. [file Beilstein_J_Org_Chem-11-2021-s001.pdf]

Supporting Information

for

Investigation on the reactivity of α-azidochalcones with carboxylic acids: Formation of α-amido-1,3-diketones and highly substituted 2-(trifluoromethyl)oxazoles

Kandasamy Rajaguru,[1] Arumugam Mariappan,[1] Rajendran Suresh,[2] Periasamy Manivannan[1] and Shanmugam Muthusubramanian[1*]

Address: [1]Department of Organic Chemistry, School of Chemistry, Madurai Kamaraj University, Madurai, 625 021, India and [2]Syngene International Limited, Biocon, Bangalore, 560 100, India

Email: Shanmugam Muthusubramanian - muthumanian2001@yahoo.com

*Corresponding author

Characterization data of new compounds 3, 7 and 8 and copies of ^{1}H, ^{13}C, two-dimentional NMR and ESI mass spectra

**Table of contents**	**Page No**
Characterization of compound **3a-3o**	3
Characterization of compound **7a & 7b**	12
Characterization of compound **8a-8e**	13
Key NMR assignments and HMBCs of compound **3e** & **7a**	16
^{1}H, ^{13}C NMR & Mass spectra of compound **3a**	17
^{1}H, ^{13}C NMR & Mass spectra of compound **3b**	20
^{1}H, ^{13}C NMR & Mass spectra of compound **3c**	23
^{1}H, ^{13}C NMR & Mass spectra of compound **3d**	26
^{1}H, ^{13}C, DEPT-135, COSY NMR & Mass spectra of compound **3e**	29
^{1}H, ^{13}C NMR & Mass spectra of compound **3f**	36
^{1}H, ^{13}C NMR & Mass spectra of compound **3g**	39
^{1}H, ^{13}C NMR & Mass spectra of compound **3h**	42
^{1}H, ^{13}C NMR & Mass spectra of compound **3i**	45
^{1}H, ^{13}C NMR & Mass spectra of compound **3j**	48
^{1}H, ^{13}C NMR & Mass spectra of compound **3k**	51
^{1}H, ^{13}C NMR & Mass spectra of compound **3l**	54
^{1}H, ^{13}C NMR & Mass spectra of compound **3m**	57
^{1}H, ^{13}C NMR & Mass spectra of compound **3n**	60
^{1}H, ^{13}C NMR & Mass spectra of compound **3o**	63
^{1}H, ^{13}C, H, H COSY, HMBC & Mass spectra of compound **4a**	66
^{1}H, ^{13}C NMR & Mass spectra of compound **4b**	71
^{1}H, ^{13}C NMR & Mass spectra of compound **8a**	75
^{1}H, ^{13}C NMR & Mass spectra of compound **8b**	78
^{1}H, ^{13}C NMR & Mass spectra of compound **8c**	81
^{1}H, ^{13}C NMR & Mass spectra of compound **8d**	84
^{1}H, ^{13}C NMR & Mass spectra of compound **8e**	87

***N*-(1-(4-Bromophenyl)-3-(4-methoxyphenyl)-1,3-dioxopropan-2-yl)-2,2,2-trifluoroacetamide (3a)**

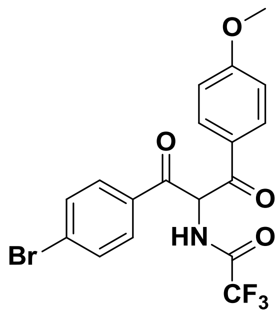

Isolated as white solid; R_f: 0.23 (EtOAc/Petether 3.5:1.5 v/v); mp: 173-174 °C; IR (KBr) 3284, 3070, 2966, 1718, 1689, 1664, 1263, 1070, 682, 570 cm^{-1}; ^{1}H NMR (300 MHz, $CDCl_3$) δ 8.01 (d, *J* = 8.0 Hz, 2H), 7.86 (d, *J* = 8.0 Hz, 2H), 7.61 (d, *J* = 8.0 Hz, 2H), 6.95 (d, *J* = 8.1 Hz, 2H), 6.77 (d, *J* = 7.1 Hz, 1H), 3.88 (s, 3H). ^{13}C NMR (75 MHz, $CDCl_3$) δ 190.6, 188.8, 165.1, 156.7, 132.9, 132.4, 131.8, 130.3, 130.0, 126.4, 117.3, 114.5, 113.6 60.4, 55.6. Anal. Calcd for: $C_{18}H_{13}BrF_3NO_4$: C, 48.67; H, 2.95; N, 3.15 %. Found: C, 48.70; H, 2.91; N, 3.12 %. ESI-MS *m/z* calcd $[M+Na]^+$ 465.99; Found: $[M+Na]^+$ 466.48.

***N*-(1-(4-Chlorophenyl)-1,3-dioxo-3-(p-tolyl)propan-2-yl)-2,2,2-trifluoroacetamide (3b)**

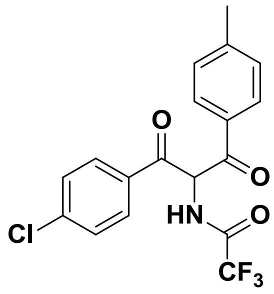

Isolated as off white solid; R_f: 0.38 (EtOAc/Petether 3.5:1.5 v/v); mp 170-172 °C; IR (KBr) 3271, 3068, 2937, 1714, 1692, 1663, 1267, 1093, 722, 687 cm^{-1}; ^{1}H NMR (300 MHz, $CDCl_3$) δ 7.96 (d, *J* = 8.6 Hz, 2H), 7.90 (d, *J* = 8.2 Hz, 2H), 7.45 (d, *J* = 8.6 Hz, 2H), 7.28 (d, *J* = 8.2 Hz, 2H), 6.82 (d, *J* = 7.4 Hz, 1H), 2.42 (s, 3H). ^{13}C NMR (75

MHz, $CDCl_3$)* δ 190.13, 190.1, 146.4, 141.4, 132.5, 131.3, 130.5, 129.9, 129.5, 129.3, 60.7, 21.8. Anal. Calcd for: $C_{18}H_{13}ClF_3NO_3$: C, 56.34; H, 3.41; N, 3.65 %. Found: C, 56.38; H, 3.43; N, 3.62 %. *The CF_3 carbon has not been picked up. ESI-MS *m/z* calcd $[M+H]^+$ 383.05; Found: $[M+H]^+$ 384.17.

***N*-(1-(4-Bromophenyl)-3-(4-methoxyphenyl)-1,3-dioxopropan-2-yl)acetamide (3c)**

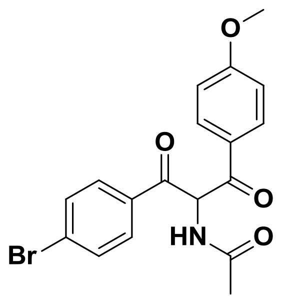

Isolated as white solid; R_f: 0.25 (EtOAc/Petether 3.5:1.5 v/v); mp: 250-251 °C; IR (KBr) 3292, 3057, 2924, 2846, 1701, 1641, 1629, 1294, 1068, 596 cm^{-1}; ^1H NMR (300 MHz, $CDCl_3$) δ 8.03 (d, J = 8.9 Hz, 2H), 7.90 (d, J = 8.5 Hz, 2H), 7.59 (d, J = 8.4 Hz, 2H), 7.02 (d, J = 7.8 Hz, 1H), 6.94 (d, J = 7.1 Hz, 2H), 3.86 (s, 3H), 2.08 (s, 3H). ^{13}C NMR (75 MHz, $CDCl_3$) δ 192.8, 191.1, 169.7, 164.6, 133.4, 132.2, 131.6, 130.4, 129.4, 127.0, 114.2, 114.0, 59.9, 55.5, 22.9. Anal. Calcd for: $C_{18}H_{16}BrNO_4$: C, 55.40; H, 4.13; N, 3.59 %. Found: C, 55.44; H, 4.18; N, 3.54 %. ESI-MS *m/z* calcd $[M+Na]^+$ 412.01; Found: $[M+Na]^+$ 413.20.

N-(1-(4-Chlorophenyl)-1,3-dioxo-3-(p-tolyl)propan-2-yl)acetamide (3d)

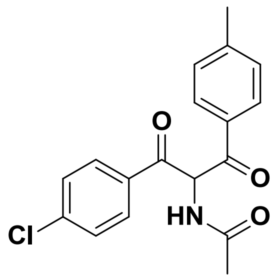

Isolated as white solid; R_f: 0.38 (EtOAc/Petether 3.5:1.5 v/v); mp: 227-229 °C; IR (KBr) 3288, 3068, 2947, 2852, 1718, 1696, 1654, 1091, 702 cm^{-1}; ^{1}H NMR (300 MHz, $CDCl_3$) δ 7.98 (d, J = 8.6 Hz, 2H), 7.91 (d, J = 8.2 Hz, 2H), 7.42 (d, J = 8.6 Hz, 2H), 7.25 (d, J = 8.2 Hz, 2H), 7.14 (d, J = 8.0 Hz, 1H), 6.98 (d, J = 8.0 Hz, 1H), 2.40 (s, 3H), 2.09 (s, 3H). ^{13}C NMR (75 MHz, $CDCl_3$) δ 192.5, 192.4, 169.6, 145.6, 140.7, 132.9, 131.7, 130.4, 129.7, 129.3, 129.2, 60.0, 23.0, 21.7. Anal. Calcd for: $C_{18}H_{16}ClNO_3$: C, 65.56; H, 4.89; N, 4.25 %. Found: C, 65.58; H, 4.83; N, 4.28 %. ESI-MS *m/z* calcd [M+H]$^+$ 329.08; Found: [M+H]$^+$ 329.96.

N-(1-(4-Chlorophenyl)-3-(4-methoxyphenyl)-1,3-dioxopropan-2-yl)acetamide (3e)

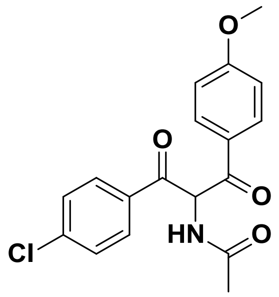

Isolated as pale yellow solid; R_f: 0.25 (EtOAc/Petether 3.5:1.5 v/v); mp: 236-237 °C; IR (KBr) 3298, 3068, 2995, 2841, 1718, 1688, 1647, 1288, 1095, 773 cm^{-1}; ^{1}H NMR (300 MHz, $CDCl_3$) δ 8.02 (d, J = 8.9 Hz, 2H), 7.97 (d, J = 8.6 Hz, 2H), 7.41 (d, J = 8.6 Hz, 2H), 7.09 (d, J = 9 Hz, 1H), 6.92 (d, J = 8.7 Hz, 3H)*, 3.86 (s, 3H), 2.08 (s, 3H). ^{13}C NMR (75 MHz, $CDCl_3$) δ 192.6, 191.2, 169.7, 164.5, 140.5, 132.9, 131.6, 130.8, 130.3, 129.1, 127.0, 114.1, 113.9, 59.9, 55.5, 22.9. Anal. Calcd for: $C_{18}H_{16}ClNO_4$: C,

62.52; H, 4.66; N, 4.05 %. Found: C, 62.57; H, 4.64; N, 4.01 %. ESI-MS *m/z* calcd $[M+H]^+$ 345.08; Found: $[M+H]^+$ 345.16. * Merge with N-H

***N*-(1,3-Dioxo-1,3-diphenylpropan-2-yl)acetamide (3f)**

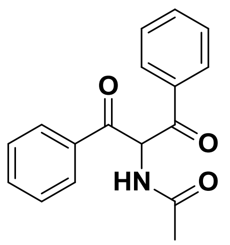

Isolated as white solid; R_f: 0.52 (EtOAc/Petether 3.5:1.5 v/v); mp: 164-166 °C; IR (KBr) 3294, 3081, 1724, 1677, 1653, 2849, 2937 cm^{-1}; ^1H NMR (300 MHz, $CDCl_3$) δ 8.04 – 8.01 (m, 4H), 7.61 – 7.56 (m, 2H), 7.47 – 7.42 (m, 4H), 7.06 (s, 2H), 2.09 (s, 3H). ^{13}C NMR (75 MHz, $CDCl_3$) δ 193.4, 169.7, 134.5, 134.2, 129.0, 128.9, 60.1, 22.9. Anal. Calcd for: $C_{17}H_{15}NO_3$: C, 72.58; H, 5.37; N, 4.98 %. Found: C, 72.62; H, 5.34; N, 4.95 %. ESI-MS *m/z* calcd $[M+H]^+$ 281.11; Found: $[M+H]^+$ 282.33.

***N*-(1-(4-Methoxyphenyl)-1,3-dioxo-3-phenylpropan-2-yl)acetamide (3g)**

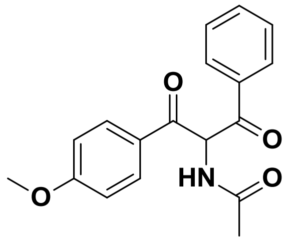

Isolated as off-white solid; R_f: 0.33 (EtOAc/Petether 3.5:1.5 v/v); mp: 161-163 °C; IR (KBr) 3287, 3064, 2831, 1721, 1636, 1627, 1286 cm^{-1}; ^1H NMR (300 MHz, $CDCl_3$) δ 7.96 – 7.90 (m, 4H), 7.61 (bs, 1H), 7.49 (t, *J* = 7.4 Hz, 1H), 7.35 (t, *J* = 7.6 Hz, 2H), 6.94 (d, *J* = 8.5 Hz, 1H), 6.83 (d, *J* = 8.9 Hz, 2H), 3.77 (s, 3H), 1.99 (s, 3H). ^{13}C NMR (75 MHz, CDCl3) δ 193.5, 191.4, 169.6, 164.1, 134.4, 133.6, 131.2, 128.6, 128.5, 127.0, 113.9, 59.5, 55.2, 22.5. Anal. Calcd for: $C_{18}H_{17}NO_4$: C, 69.44; H, 5.50; N, 4.50

%. Found: C, 69.47; H, 5.52; N, 4.47 %. ESI-MS *m/z* calcd $[M+Na]^+$ 334.11; Found: $[M+Na]^+$ 334.24.

N-(1-(4-Chlorophenyl)-1,3-dioxo-3-(thiophen-2-yl)propan-2-yl)acetamide (3h)

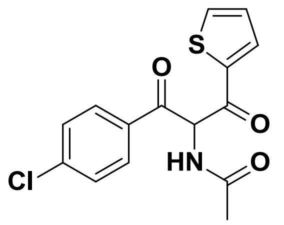

Isolated as brown solid; R_f: 0.71 (EtOAc/Petether 3.5:1.5 v/v); mp 189-190 °C; IR (KBr) 3298, 3172, 2931, 2852, 1701, 1695, 1643, 1091, 738 cm^{-1}; ^1H NMR (300 MHz, $CDCl_3$) δ 7.99 (d, J = 8.6 Hz, 2H), 7.75 (d, J = 4.9 Hz, 1H), 7.44 (d, J = 8.6 Hz, 2H), 7.20 – 7.14 (m, 2H), 6.82 (d, J = 7.9 Hz, 1H), 2.10 (s, 3H). ^{13}C NMR (75 MHz, $CDCl_3$) δ 191.9, 185.3, 169.9, 140.8, 140.6, 136.1, 134.7, 132.6, 130.2, 129.1, 128.6, 61.0, 22.7. Anal. Calcd for: $C_{15}H_{12}ClNO_3S$: C, 55.99; H, 3.76; N, 4.35, S, 9.96 %. Found: C, 55.96; H, 3.80; N, 4.31, S 9.98 %. ESI-MS *m/z* calcd $[M+Na]^+$ 344.0; Found: $[M+Na]^+$ 344.24.

2-Chloro-N-(1-(4-chlorophenyl)-1,3-dioxo-3-(p-tolyl)propan-2-yl)acetamide (3i)

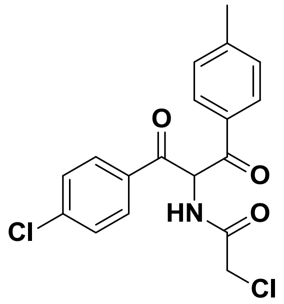

Isolated as white solid; R_f: 0.51 (EtOAc/Petether 3.5:1.5 v/v); mp: 253-255 °C; IR (KBr) 3277, 3066, 2858, 1718, 1701, 1664, 1458, 1089, 786 cm^{-1}; ^1H NMR (300 MHz, $CDCl_3$) δ 8.09 (d, J = 7.4 Hz, 1H), 7.98 (d, J = 8.7 Hz, 2H), 7.92 (d, J = 8.3 Hz,

2H), 7.44 (d, J = 8.7 Hz, 2H), 7.27 (d, J = 6.0 Hz, 2H), 6.85 (d, J = 7.7 Hz, 1H), 4.10 (s, 2H), 2.41 (s, 3H). ^{13}C NMR (75 MHz, $CDCl_3$) δ 191.4**, 165.8, 145.8, 140.9, 132.7, 131.5, 130.4, 129.7, 129.3,* 128.8, 128.5, 60.9, 42.3, 21.7. Anal. Calcd for: $C_{18}H_{15}Cl_2NO_3$: C, 59.36; H, 4.15; N, 3.85 %. Found: C, 59.38; H, 4.13; N, 3.89 %. ** Two carbonyl carbon signals merged here; *Two aryl carbon signals mergrd here. ESI-MS m/z calcd $[M+Na]^+$ 386.02; Found: $[M+Na]^+$ 386.05.

***N*-(1-(4-Bromophenyl)-3-(4-methoxyphenyl)-1,3-dioxopropan-2-yl)-2-chloroacetamide (3j)**

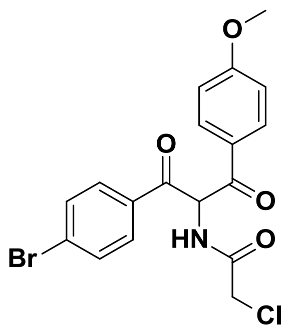

Isolated as white solid; R_f: 0.48 (EtOAc/Petether 3.5:1.5 v/v); mp: 164-166 °C; IR (KBr) 3282, 3066, 2841, 1701, 1685, 1654, 1463, 1294, 1070, 788, 582 cm^{-1}; ^{1}H NMR (300 MHz, $CDCl_3$) δ 8.03 (d, J = 8.3 Hz, 3H), 7.89 (d, J = 7.7 Hz, 2H), 7.61 (d, J = 7.5 Hz, 2H), 6.95 (d, J = 8.0 Hz, 2H), 6.81 (d, J = 6.9 Hz, 1H), 4.10 (s, 2H), 3.87 (s, 3H). ^{13}C NMR (75 MHz, $CDCl_3$) δ 191.8, 190.1, 166.0, 164.7, 133.1, 132.2, 131.7, 130.4, 129.6, 126.8, 114.3, 60.8, 55.6, 42.2. Anal. Calcd for: $C_{18}H_{15}BrClNO_4$: C, 50.91; H, 3.56; N, 3.30 %. Found: C, 50.93; H, 3.58; N, 3.28 %. ESI-MS m/z calcd $[M+Na]^+$ 445.97; Found: $[M+Na]^+$ 446.26.

***N*-(1-(4-Chlorophenyl)-3-(4-methoxyphenyl)-1,3-dioxopropan-2-yl)-4-nitrobenzamide (3k)**

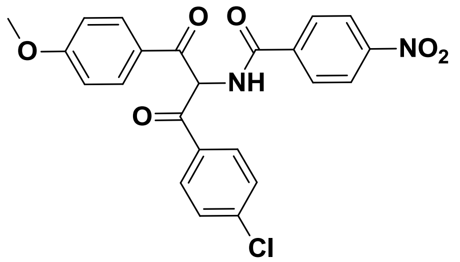

Isolated as white solid; R_f: 0.18 (EtOAc/Petether 3.5:1.5 v/v); mp: 178-180 °C; IR (KBr) 3325, 3066, 2841, 1701, 1684, 1643, 1527, 1290, 1091, 721 cm^{-1}; ^{1}H NMR (300 MHz, $CDCl_3$) δ 8.30 (d, J = 8.8 Hz, 2H), 8.08 (d, J = 9.0 Hz, 2H), 8.03 (d, J = 8.4 Hz, 3H), 7.93 (d, J = 7.4 Hz, 1H), 7.45 (d, J = 8.6 Hz, 2H), 7.08 (d, J = 7.6 Hz, 1H), 6.95 (d, J = 8.9 Hz, 2H), 3.88 (s, 3H). ^{13}C NMR (75 MHz, $CDCl_3$) δ 192.1, 190.7, 164.9, 164.8, 149.8, 140.9, 138.5, 132.8, 131.7, 130.4, 129.3, 128.6, 126.8, 123.7, 114.3, 60.5, 55.6. Anal. Calcd for: $C_{23}H_{17}ClN_2O_6$: C, 61.00; H, 3.78; N, 6.19 %. Found: C, 61.05; H, 3.81; N, 6.14 %. ESI-MS *m/z* calcd $[M+Na]^+$ 475.07; Found: $[M+Na]^+$ 475.82.

***N*-(1-(4-Chlorophenyl)-1,3-dioxo-3-(p-tolyl)propan-2-yl)-4-nitrobenzamide (3l)**

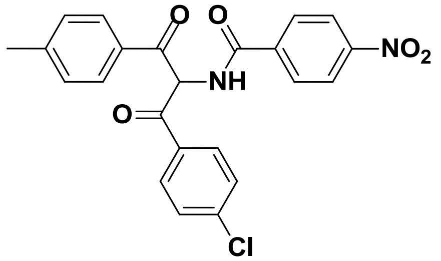

Isolated as white solid; R_f: 0.20 (EtOAc/Petether 3.5:1.5 v/v); mp: 152-153 °C; IR (KBr) 3327, 3034, 2920, 2850, 1718, 1687, 1654, 1560, 1093, 761 cm^{-1}; ^{1}H NMR (300 MHz, $CDCl_3$) δ 8.24 (d, J = 8.8 Hz, 2H), 8.06 (d, J = 7.8 Hz, 1H), 8.07 – 8.01 (m, 5H)*, 7.94 (d, J = 8.2 Hz, 2H), 7.43 (d, J = 8.6 Hz, 2H), 7.26 (d, J = 8.0 Hz, 2H),

7.16 (d, J = 7.8 Hz, 1H), 2.41 (s, 3H). ^{13}C NMR (75 MHz, $CDCl_3$) δ 191.93, 191.88, 164.9, 149.9, 146.0, 141.0, 138.6, 132.8, 131.5, 130.5, 129.8, 129.4, 129.2, 128.6, 123.8, 60.6, 21.8. Anal. Calcd for: $C_{23}H_{17}ClN_2O_5$: C, 63.24; H, 3.92; N, 6.41 %. Found: C, 63.28; H, 3.96; N, 6.45 %. ESI-MS *m/z* calcd $[M+H]^+$ 436.08; Found: $[M+H]^+$ 436.31. *merged with N-H

_N_-(1-(4-Chlorophenyl)-1,3-dioxo-3-(p-tolyl)propan-2-yl)benzamide (3m)

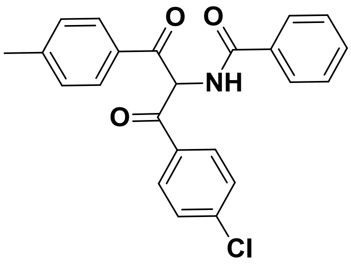

Isolated as pale yellow solid; R_f: 0.48 (EtOAc/Petether 3.5:1.5 v/v); mp: 168-170 °C; IR (KBr) 3336, 3051, 2960, 2854, 1718, 1707, 1637, 1093, 781 cm^{-1}; ^{1}H NMR (300 MHz, $CDCl_3$) δ 8.06 (d, J = 8.6 Hz, 2H), 7.98 (d, J = 8.1 Hz, 2H), 7.86 (d, J = 7.9 Hz, 2H), 7.56 – 7.43 (m, 5H), 7.27 (d, J = 8.4 Hz, 2H), 7.14 (d, J = 7.8 Hz, 1H), 2.40 (s, 3H). ^{13}C NMR (75 MHz, $CDCl_3$) δ 192.43, 192.38 166.8, 145.6, 140.8, 133.23, 133.20, 132.2, 132.0, 130.5, 129.9, 129.8, 129.3, 128.9, 128.7, 128.5, 127.3, 60.8, 21.7. Anal. Calcd for: $C_{23}H_{18}ClNO_3$: C, 70.50; H, 4.63; N, 3.57 %. Found: C, 70.54; H, 4.66; N, 3.52 %. ESI-MS *m/z* calcd $[M+Na]^+$ 414.08; Found: $[M+Na]^+$ 414.37.

2-Chloro-*N*-(1-(4-chlorophenyl)-1,3-dioxo-3-(p-tolyl)propan-2-yl)benzamide (3n)

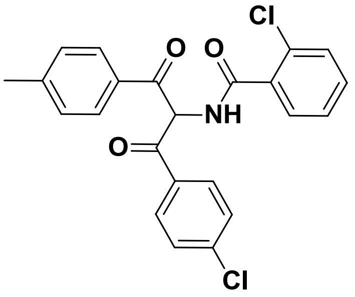

Isolated as white solid; R_f: 0.45 (EtOAc/Petether 3.5:1.5 v/v); mp: 184-185 °C; IR (KBr) 3267, 3064, 2954, 2852, 1707, 1691, 1654, 1093, 1037, 783 cm^{-1}; ^{1}H NMR (300 MHz, $CDCl_3$) δ 8.04 (d, *J* = 8.5 Hz, 2H), 7.98 (d, *J* = 8.1 Hz, 2H), 7.87 (d, *J* = 7.6 Hz, 1H), 7.63 (d, *J* = 6.9 Hz, 1H), 7.45 – 7.39 (m, 4H), 7.27 (d, *J* = 6.9 Hz, 2H), 7.08 (d, *J* = 7.6 Hz, 1H), 2.41 (s, 3H). ^{13}C NMR (75 MHz, $CDCl_3$) δ 191.97, 191.91, 165.8, 145.7, 140.8, 133.9, 133.6, 133.1, 131.9, 131.3, 130.6, 130.5, 130.3, 129.7, 129.4, 129.3, 127.1, 61.7, 21.8. Anal. Calcd for: $C_{23}H_{17}Cl_2NO_3$: C, 64.80; H, 4.02; N, 3.29 %. Found: C, 64.86; H, 4.05; N, 3.26 %. ESI-MS *m/z* calcd $[M+Na]^+$ 448.05; Found: $[M+Na]^+$ 448.10.

4-Chloro-*N*-(1-(4-chlorophenyl)-1,3-dioxo-3-(p-tolyl)propan-2-yl)-2,5-difluorobenzamide (3o)

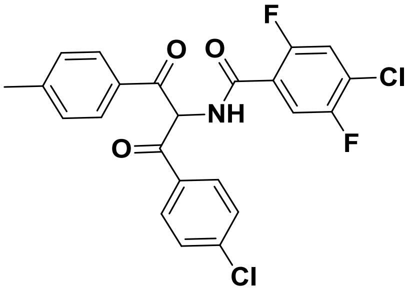

Isolated as white solid; R_f: 0.53 (EtOAc/Petether 3.5:1.5 v/v); mp: 177-179 °C; IR (KBr) 3284, 3066, 2920, 2850, 1718,, 1698, 1654, 1238, 1091, 761 cm^{-1}; ^{1}H NMR (300 MHz, $CDCl_3$) δ 8.01 (d, *J* = 8.1 Hz, 2H), 7.96 (d, *J* = 7.7 Hz, 2H), 7.69 (d, *J* = 6.7

Hz, 1H), 7.44 (d, J = 8.0 Hz, 2H), 7.27 (d, J = 6.5 Hz, 3H), 7.04 – 6.99 (m, 3H), 2.41 (s, 3H). ^{13}C NMR (75 MHz, $CDCl_3$)* δ 191.6, 191.5, 161.9, 159.1, 145.9, 140.9, 137.7, 132.9, 131.6, 130.5, 129.8, 129.4, 129.3, 129.1, 128.9, 113.6, 113.3, 61.5, 21.8. Anal. Calcd for: $C_{23}H_{15}Cl_2F_2NO_3$: C, 59.76; H, 3.27; N, 3.03 %. Found: C, 59.79; H, 3.24; N, 3.01 %. *One aromatic carbon not picked up. ESI-MS *m/z* calcd $[M+Na]^+$ 484.02 ; Found: $[M+Na]^+$ 484.14.

(5-(4-Chlorophenyl)-2-methyloxazol-4-yl)(4-methoxyphenyl)methanone (7a)

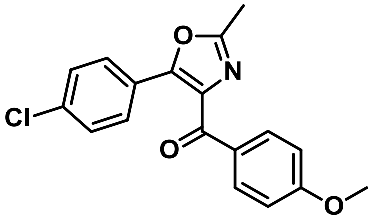

Isolated as off white solid; R_f: 0.82 (EtOAc/Petether 4.5:0.5 v/v); mp 152-153 °C; IR (KBr) 3034, 2922, 1850, 1718, 1602, 1288, 1095, 773 cm^{-1}; ^{1}H NMR (300 MHz, $CDCl_3$) δ 8.11 (d, J = 8.7 Hz, 2H), 7.93 (d, J = 8.6 Hz, 2H), 7.39 (d, J = 8.6 Hz, 2H), 6.95 (d, J = 8.8 Hz, 2H), 3.88 (s, 3H), 2.58 (s, 3H). ^{13}C NMR (75 MHz, $CDCl_3$) δ 187.1, 163.6, 158.9, 153.0, 135.7, 134.3, 132.7, 130.1, 128.7, 128.67, 125.9, 113.5, 55.42, 13.8. Anal. Calcd for: $C_{18}H_{14}ClNO_3$: C, 65.96; H, 4.31; N, 4.27 %. Found: C, 65.98; H, 4.34; N, 4.24 %. ESI-MS *m/z* calcd $[M+Na]^+$ 350.06; Found: $[M+Na]^+$ 350.14.

(5-(4-Chlorophenyl)-2-methyloxazol-4-yl)(thiophen-2-yl)methanone (7b)

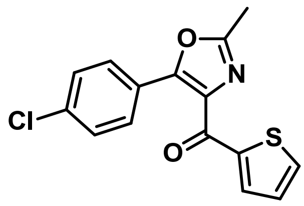

Isolated as pale brown solid; R_f: 0.94 (EtOAc/Petether 4.5:0.5 v/v); mp 170-172 °C; IR (KBr) 3169, 3039, 2933, 2854, 1704, 1606, 1093, 741 cm^{-1}; ^{1}H NMR (300 MHz, $CDCl_3$) δ 8.34 (dd, J = 3.9, 1.2 Hz, 1H), 8.03 (d, J = 8.8 Hz, 2H), 7.64 (dd, J = 5.0, 1.2 Hz, 1H), 7.35 (d, J = 8.8 Hz, 2H), 7.10 (dd, J = 4.9, 3.9 Hz, 1H), 2.53 (s, 3H). ^{13}C NMR (75 MHz, $CDCl_3$) δ 178.8, 158.9, 153.9, 143.4, 136.1, 135.8, 134.9, 133.6, 129.3, 128.6, 127.9, 125.7, 13.78. Anal. Calcd for: $C_{15}H_{10}ClNO_2S$: C, 59.31; H, 3.32; N, 4.61; S, 10.56 %. Found: C, 59.35; H, 3.34; N, 4.58; S, 10.59 %. ESI-MS *m/z* calcd $[M+Na]^+$ 326.00; Found: $[M+Na]^+$ 326.75.

Phenyl(5-phenyl-2-(trifluoromethyl)oxazol-4-yl)methanone (8a)

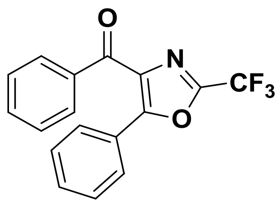

Isolated as yellow oil; R_f: 0.32 (EtOAc/Petether 4:1 v/v); IR (KBr) 3066, 1709, 1612, 1288, 684 cm^{-1}; ^{1}H NMR (400 MHz, DMSO-d_6) δ: 8.09 (d, J = 8.0 Hz, 2H), 8.03 (t, J = 8.0 Hz, 2H), 7.62 (t, J = 7.6 Hz, 1H), 7.47-7.52 (m, 5H). ^{13}C NMR (100 MHz, DMSO-d_6) δ: 187.7, 156.8, 147.7, 136.5, 133.8, 133.6, 131.4, 130.3, 128.8, 128.4, 128.2, 125.7, 116.3. Anal. Calcd for: $C_{17}H_{10}F_3NO_2$: C, 64.36; H, 3.18; N, 4.14 %. Found: C, 64.30; H, 3.14; N, 4.19 %. ESI-MS *m/z* calcd $[M+H]^+$ 317.07; Found: $[M+H]^+$ 318.20.

(2-Bromophenyl)(5-(4-chlorophenyl)-2-(trifluoromethyl)oxazol-4-yl)methanone (8b)

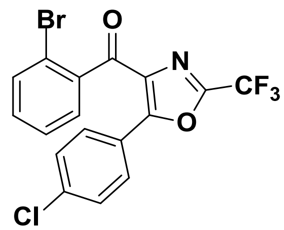

Isolated as pale yellow oil; *R*f: 0.41 (EtOAc/Petether 4:1 v/v); IR (KBr) 3054, 1702, 1601, 1291, 1083, 1031, 723, 672 cm^{-1}; ^{1}H NMR (400 MHz, $CDCl_3$) δ: 8.14 (d, *J* = 8.4 Hz, 2H), 7.71 (dd, *J* = 8.0, 1.2 Hz, 1H), 7.57 (dd, *J* = 8.8, 2.0 Hz, 1H), 7.43-7.48 (m, 3H), 7.42 (td, *J* = 7.6, 2.0 Hz, 1H). ^{13}C NMR (100 MHz, $CDCl_3$) δ: 184.1, 156.1, 149.0, 140.2, 135.6, 134.2, 133.4, 132.5, 132.3, 131.7, 128.8,* 127.4, 123.5, 116.1. Anal. Calcd for: $C_{17}H_8BrClF_3NO_2$: C, 47.42; H, 1.87; N, 3.25 %. Found: C, 47.38; H, 1.82; N, 3.29 %. *Two carbons merged here. ESI-MS *m/z* calcd $[M+Na]^+$ 453.59; Found: $[M+Na]^+$ 453.7.

(5-(3-Methoxyphenyl)-2-(trifluoromethyl)oxazol-4-yl)(4-nitrophenyl)methanone (8c)

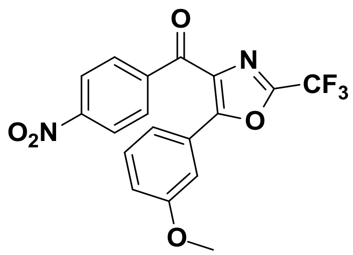

Isolated as yellow oil; *R*f: 0.25 (EtOAc/Petether 4:1 v/v); IR (KBr) 3043, 1701, 1609, 1563, 1288, 1272, 679 cm^{-1}; ^{1}H NMR (400 MHz, $CDCl_3$) δ: 8.35 (d, *J* = 9.2 Hz, 2H), 8.26 (d, *J* = 9.2 Hz, 2H), 7.71 (d, *J* = 7.6 Hz, 1H), 7.64 (t, *J* = 2.0 Hz, 1H), 7.44 (t, *J* = 8.0 Hz, 1H), 7.21 (dd, *J* = 8.0, 2.0 Hz, 1H), 3.88 (s, 3H). ^{13}C NMR (100 MHz, $CDCl_3$) δ: 186.6, 159.7, 154.0, 149.0, 137.1, 135.9, 131.3, 129.6, 129.3, 129.2, 123.9, 123.8, 123.5, 120.8,114.3, 55.5. Anal. Calcd for: $C_{18}H_{11}F_3N_2O_5$: C, 55.11; H, 2.83; N, 7.14

%. Found: C, 55.15; H, 2.87; N, 7.09 %. ESI-MS *m/z* calcd $[M+H]^+$ 392.06; Found: $[M+H]^+$ 392.1.

(5-(2-Chloro-3,4-dimethoxyphenyl)-2-(trifluoromethyl)oxazol-4-yl)(4-methoxyphenyl)methanone (8d)

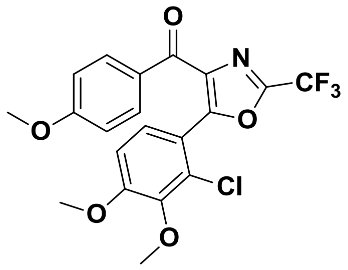

Isolated as yellow oil; R_f: 0.23 (EtOAc/Petether 4:1 v/v); IR (KBr) 3072, 1711, 1609, 1287, 1051, 1273, 718, 688 cm^{-1}; ^1H NMR (400 MHz, $CDCl_3$) δ: 8.16 (d, J = 8.4 Hz, 2H), 7.37 (d, J = 8.4 Hz, 1H), 6.95 (d, J = 8.4 Hz, 2H), 6.93 (d, J = 8.4 Hz, 1H), 3.94 (s, 3H), 3.88 (s, 3H), 3.87 (s, 3H). ^{13}C NMR (100 MHz, $CDCl_3$) δ: 184.4, 163.9, 155.8, 154.0, 148.5, 145.9, 136.2, 132.8, 128.9, 128.7, 127.5, 118.3,* 116.3, 114.6, 113.7, 110.2, 54.3. Anal. Calcd for: $C_{20}H_{15}ClF_3NO_5$: C, 54.37; H, 3.42; N, 3.17 %. Found: C, 54.32; H, 3.38; N, 3.23 % * Two carbon signals merged here. ESI-MS *m/z* calcd $[M+H]^+$ 441.06; Found: $[M+H]^+$ 441.9.

(2,4-Dichlorophenyl)(5-(p-tolyl)-2-(trifluoromethyl)oxazol-4-yl)methanone (8e)

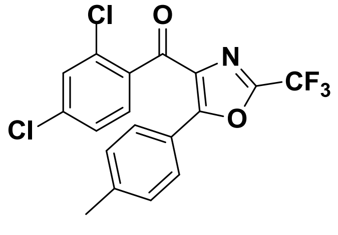

Isolated as yellow oil; R_f: 0.31 (EtOAc/Petether 4:1 v/v); IR (KBr) 3068, 2849, 2960, 1703, 1602, 1281, 1053, 720, 672 cm^{-1}; ^1H NMR (400 MHz, $CDCl_3$) δ: 8.06 (d, J = 8.4 Hz, 2H), 7.58 (d, J = 8.4 Hz, 1H), 7.53 (d, J = 1.6 Hz, 1H), 7.40 (dd, J = 8.4, 1.6 Hz, 1H), 7.29 (d, J =

8.4 Hz, 2H), 2.44 (s, 3H). ^{13}C NMR (100 MHz, $CDCl_3$) δ: 185.4, 153.1, 144.8, 137.9, 136.8, 134.8, 133.1, 132.7, 130.4,* 130.2, 129.1, 127.3, 123.9, 117.4, 21.7; Anal. Calcd for: $C_{18}H_{10}Cl_2F_3NO_2$: C, 54.02; H, 2.52; N, 3.50 %. Found C, 53.93; H, 2.46; N, 3.57 % *Two carbons merged here. ESI-MS *m/z* calcd $[M+H]^+$ 399.0; Found: $[M+H]^+$ 399.9.

Key NMR assignments and HMBCs of compound **3e**

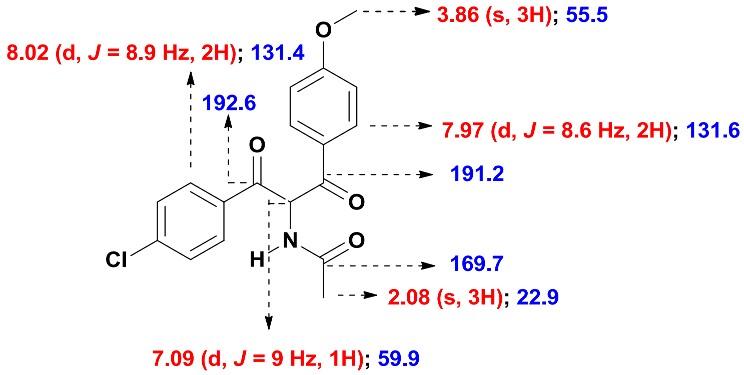

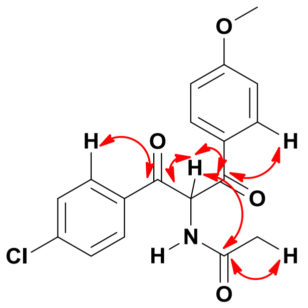

Key HMBC assignment in compound **7a**

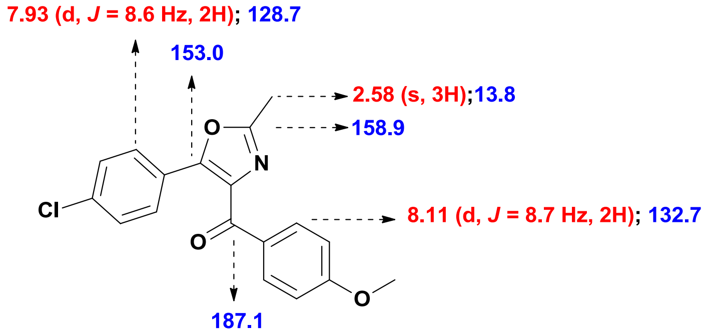

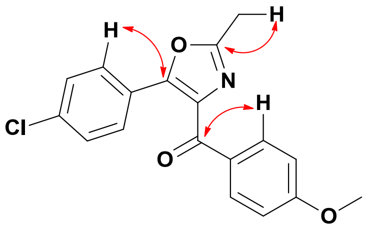

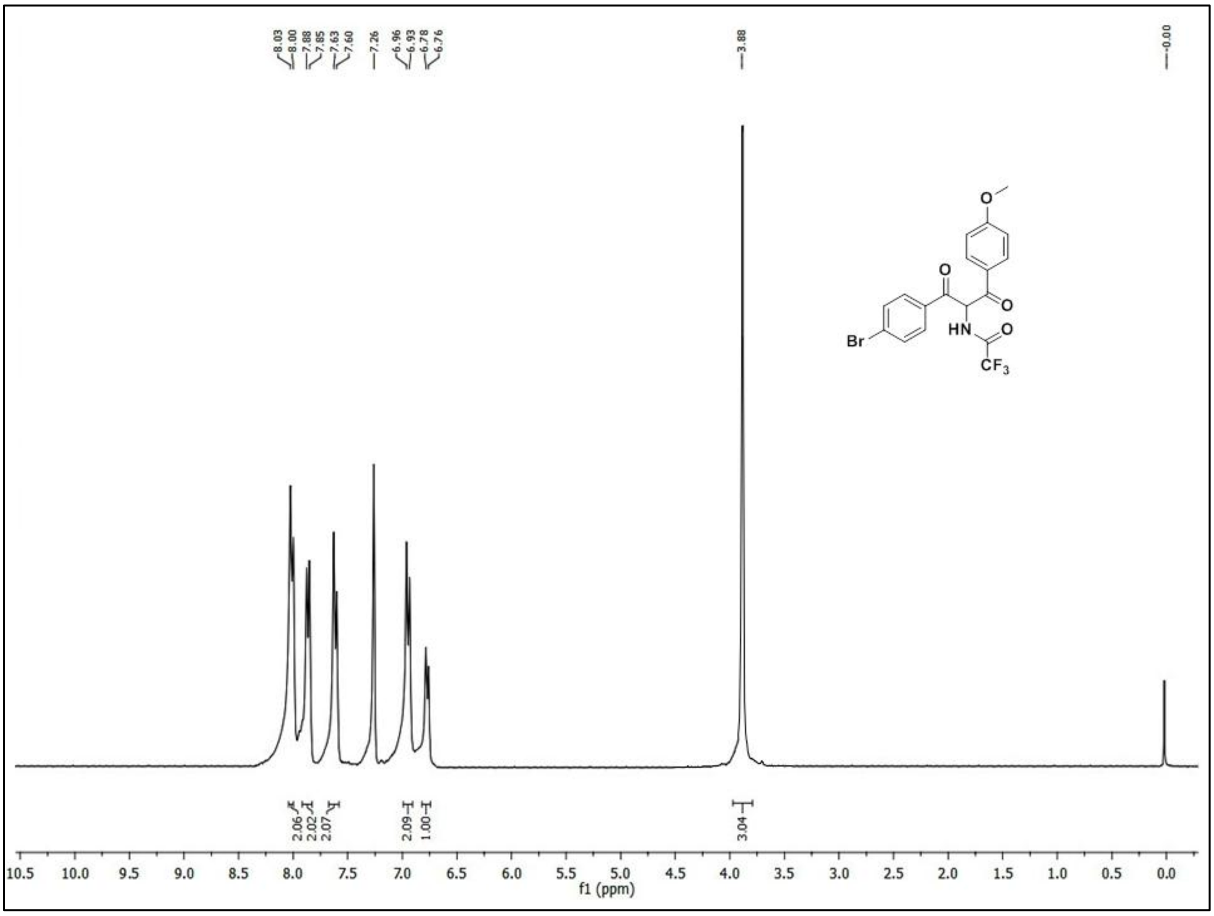

Fig. 1: ^{1}H NMR Spectrum of compound **3a**

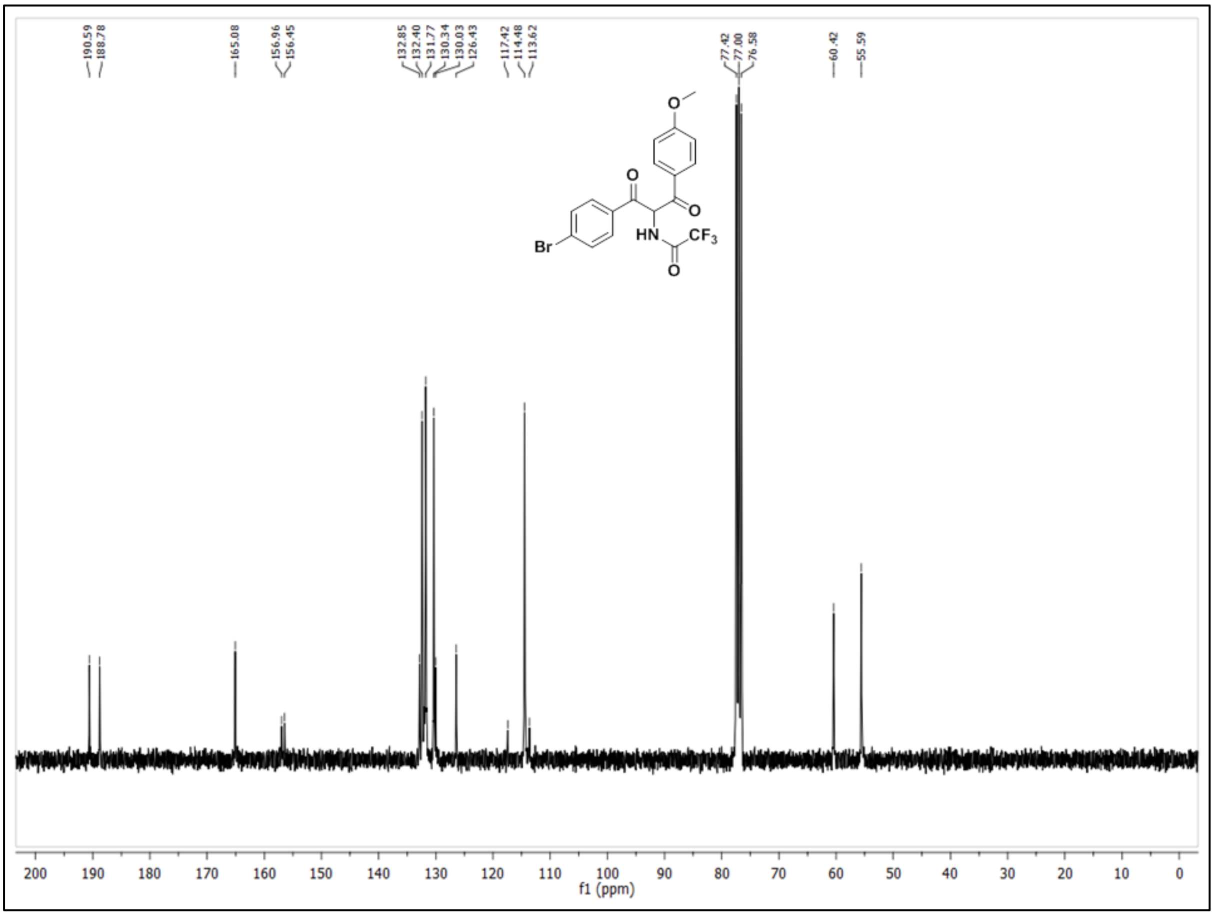

Fig. 2: ^{13}C NMR Spectrum of compound **3a**

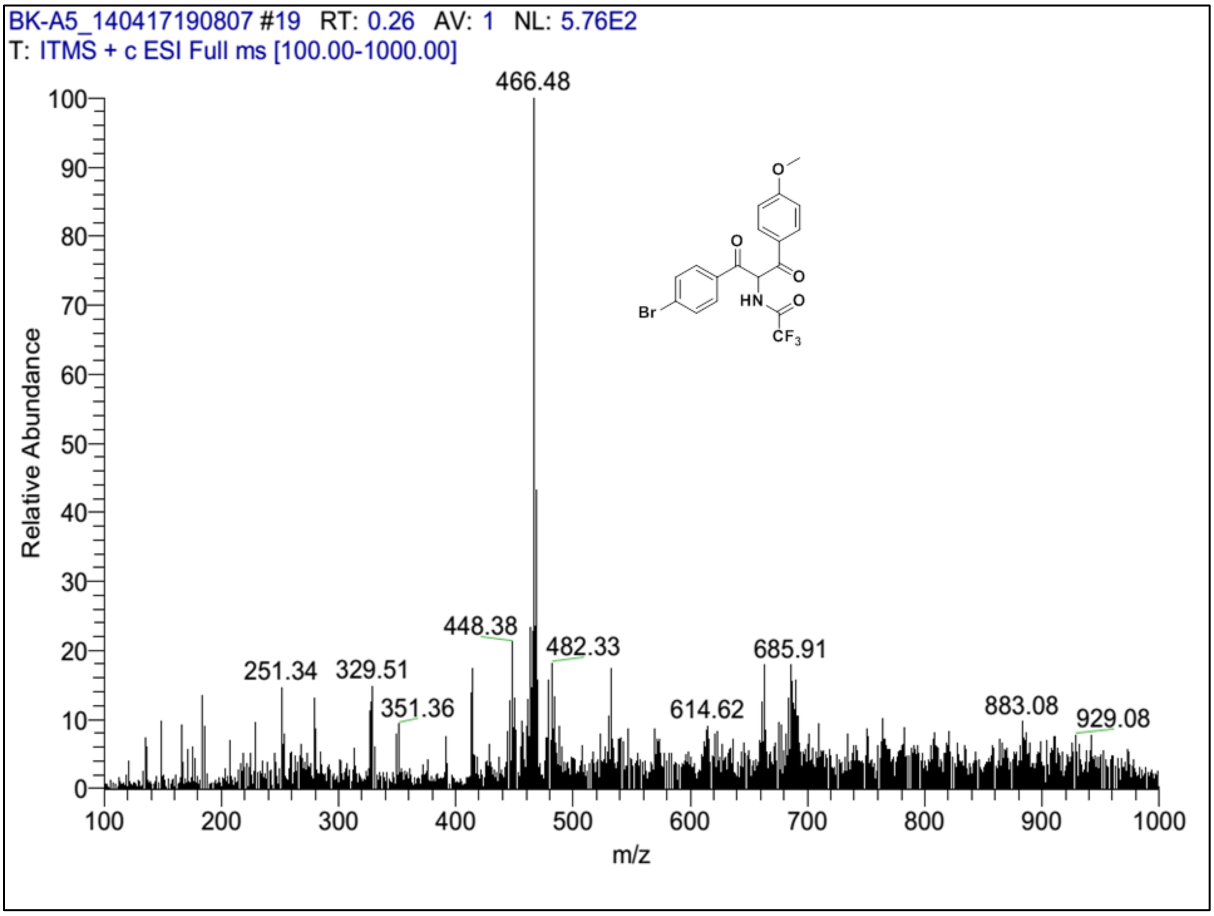

Fig. 3: Mass Spectrum of compound **3a**

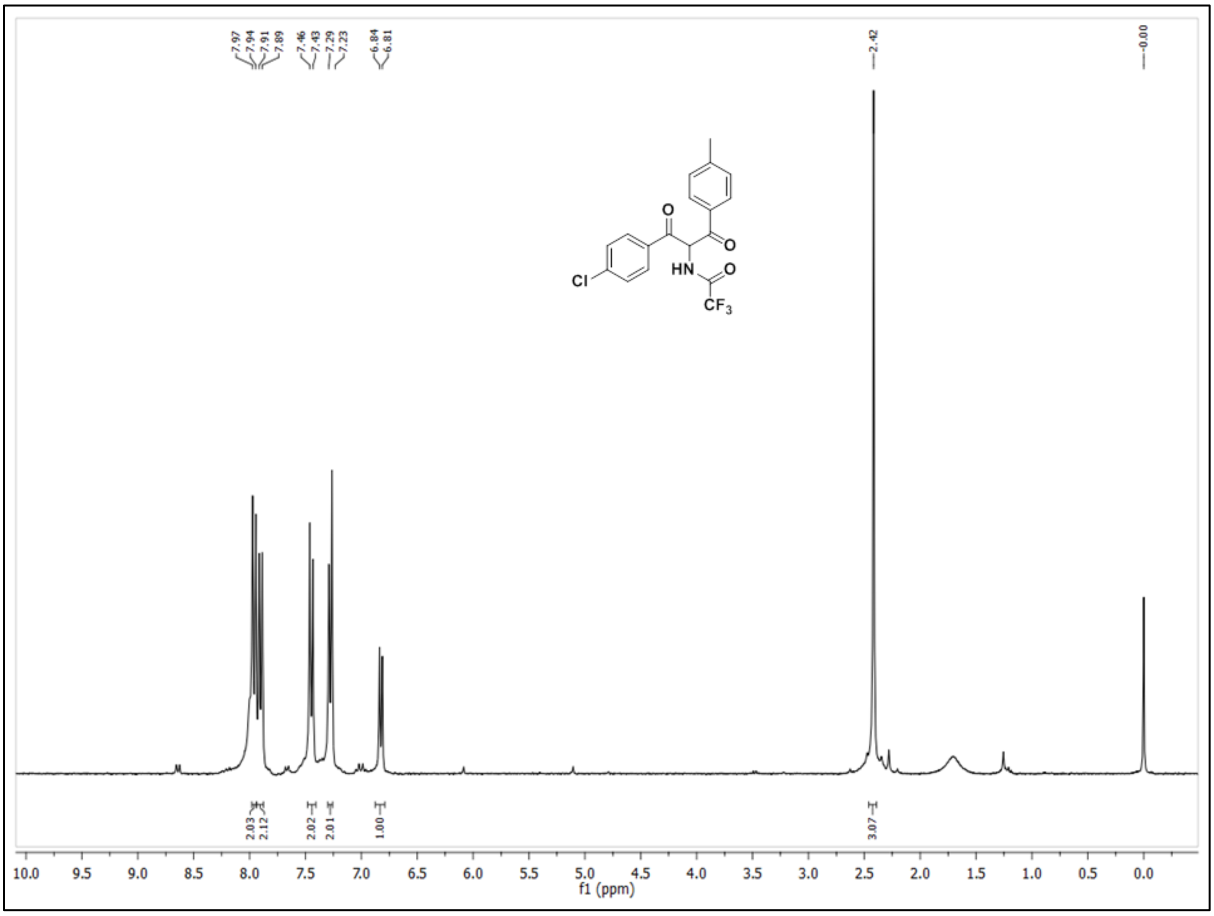

Fig. 4: ^{1}H NMR Spectrum of compound **3b**

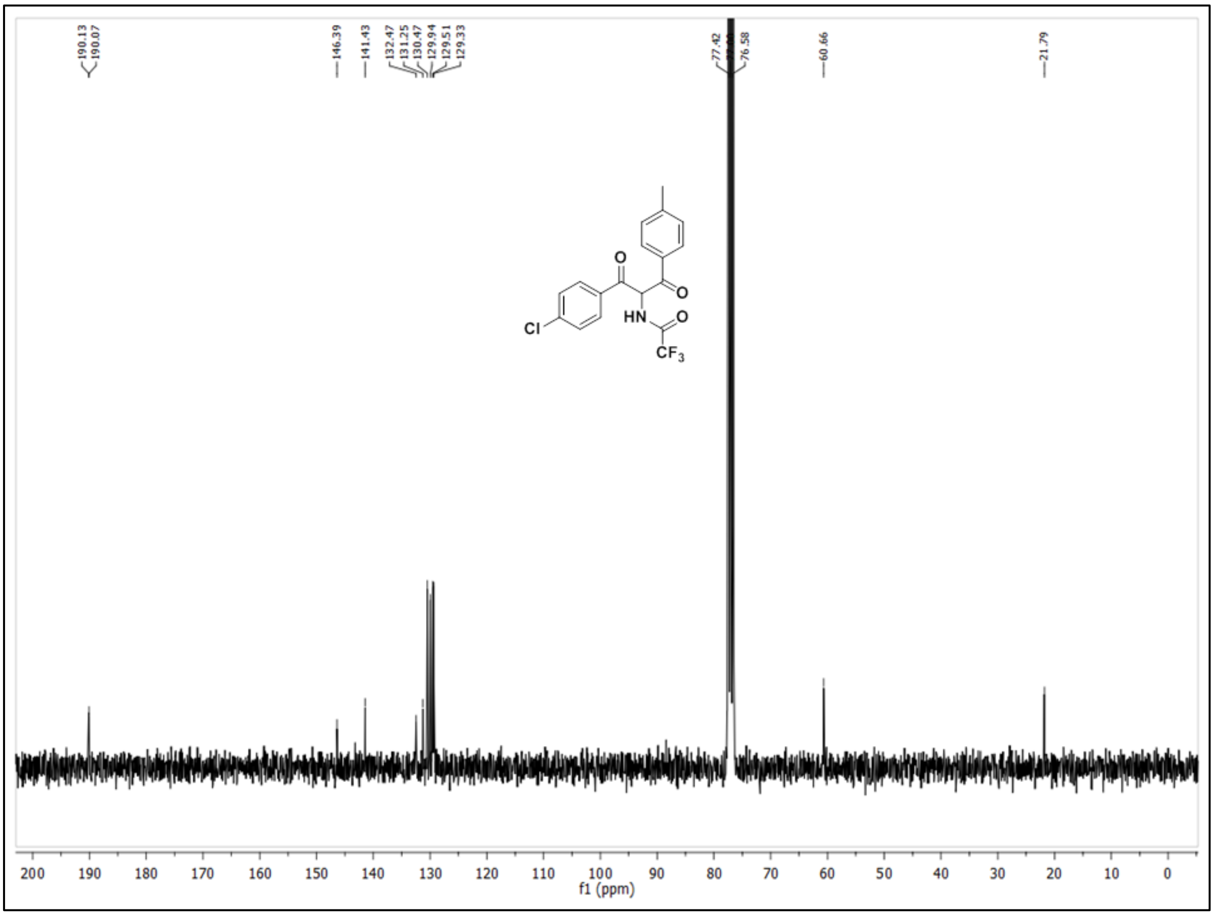

Fig. 5: ^{13}C NMR Spectrum of compound **3b**

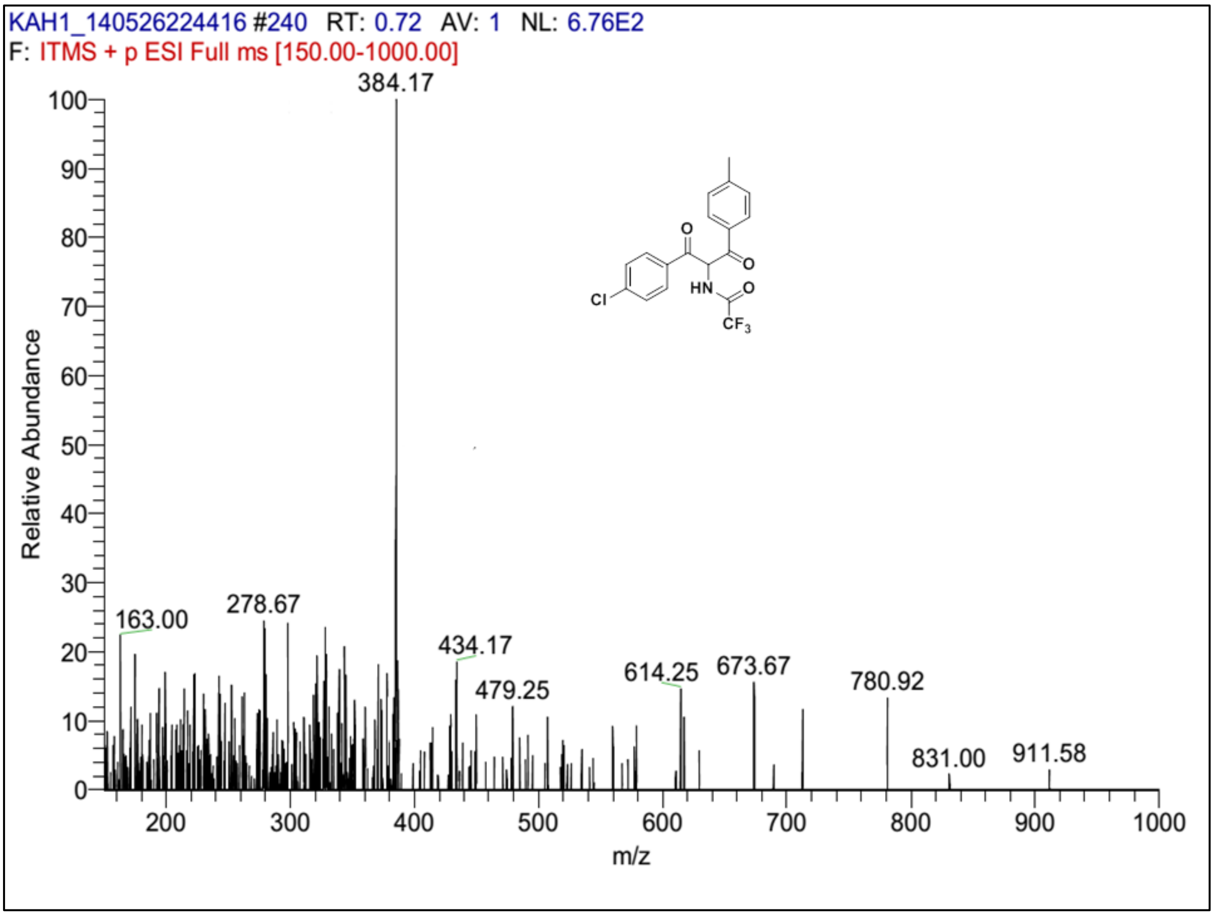

Fig. 6: Mass Spectrum of compound **3b**

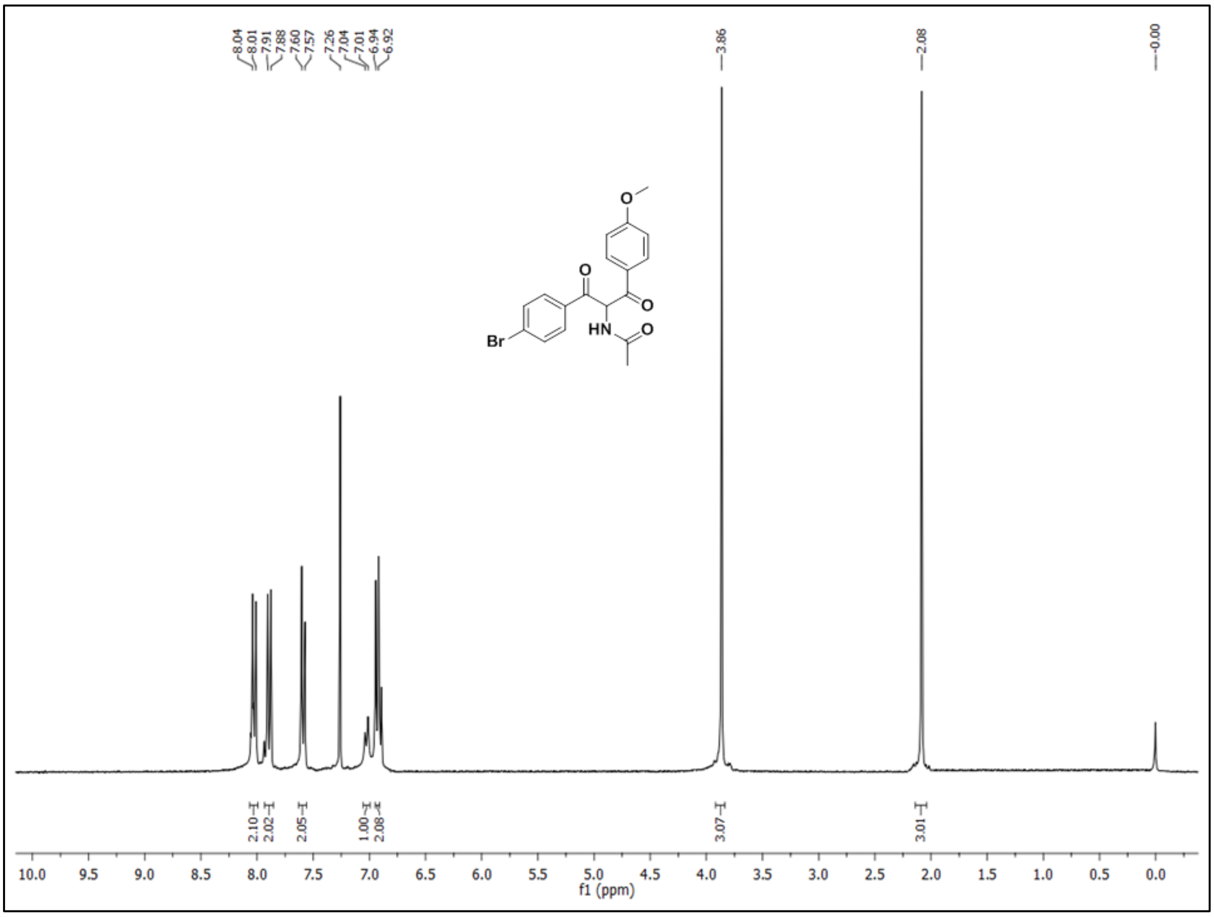

Fig. 7: ^{1}H NMR Spectrum of compound **3c**

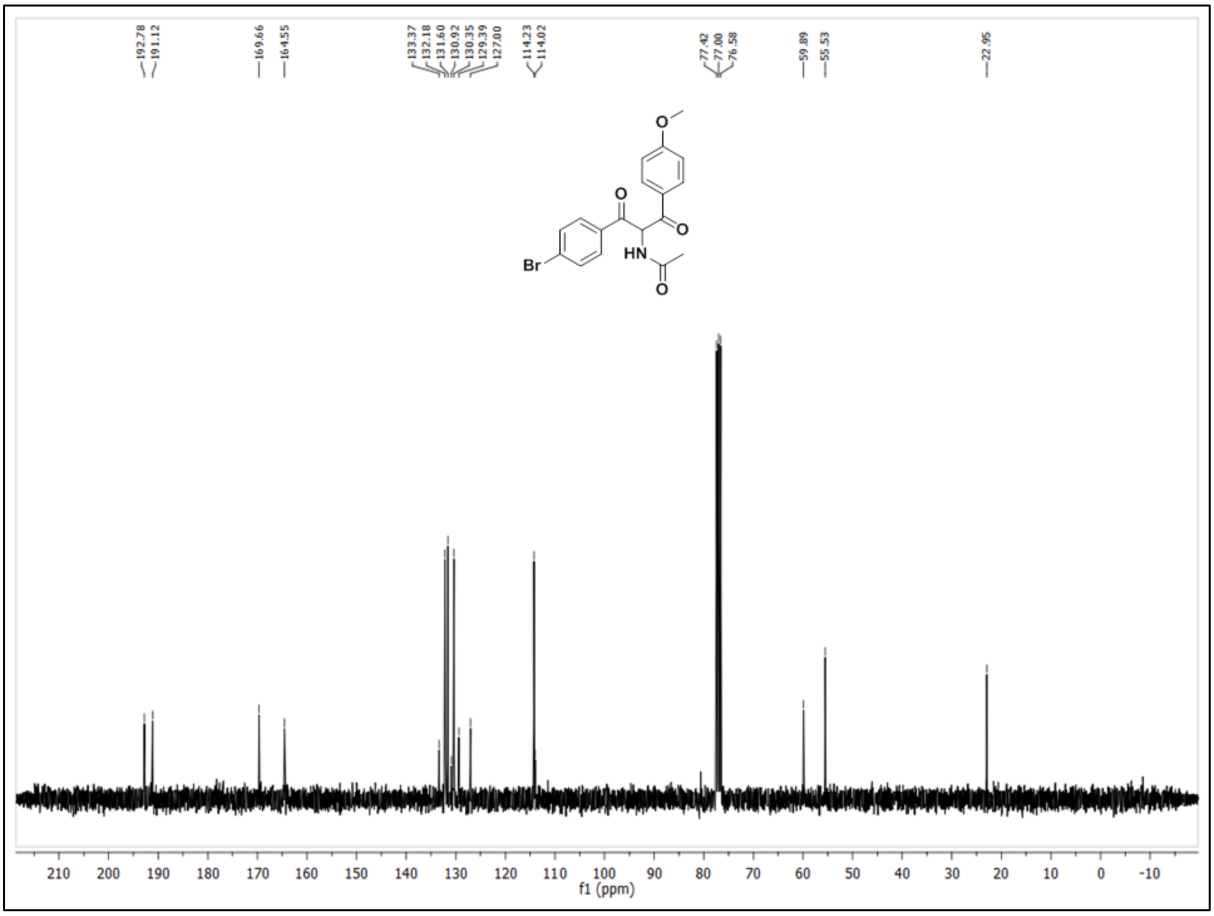

Fig. 8: ^{13}C NMR Spectrum of compound **3c**

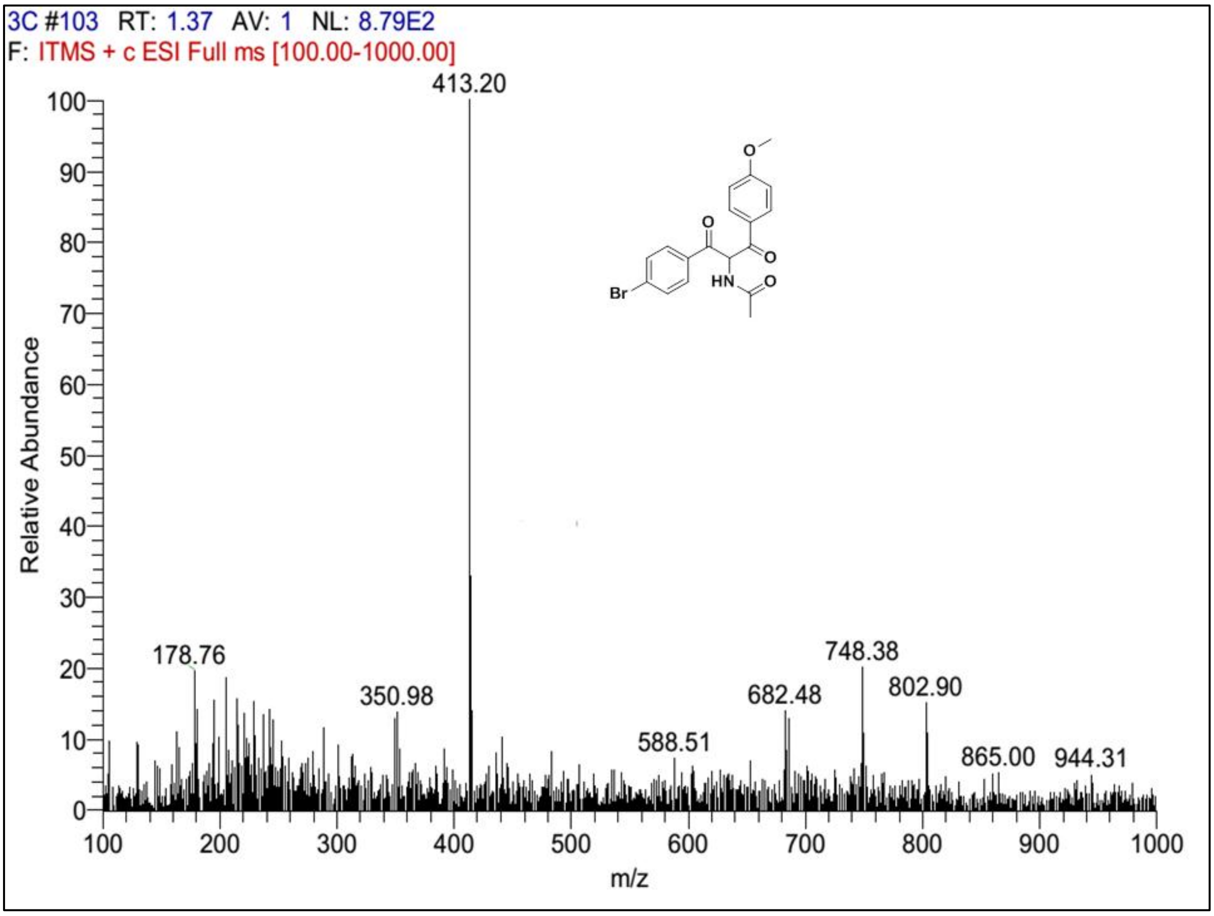

Fig. 9: Mass Spectrum of compound **3c**

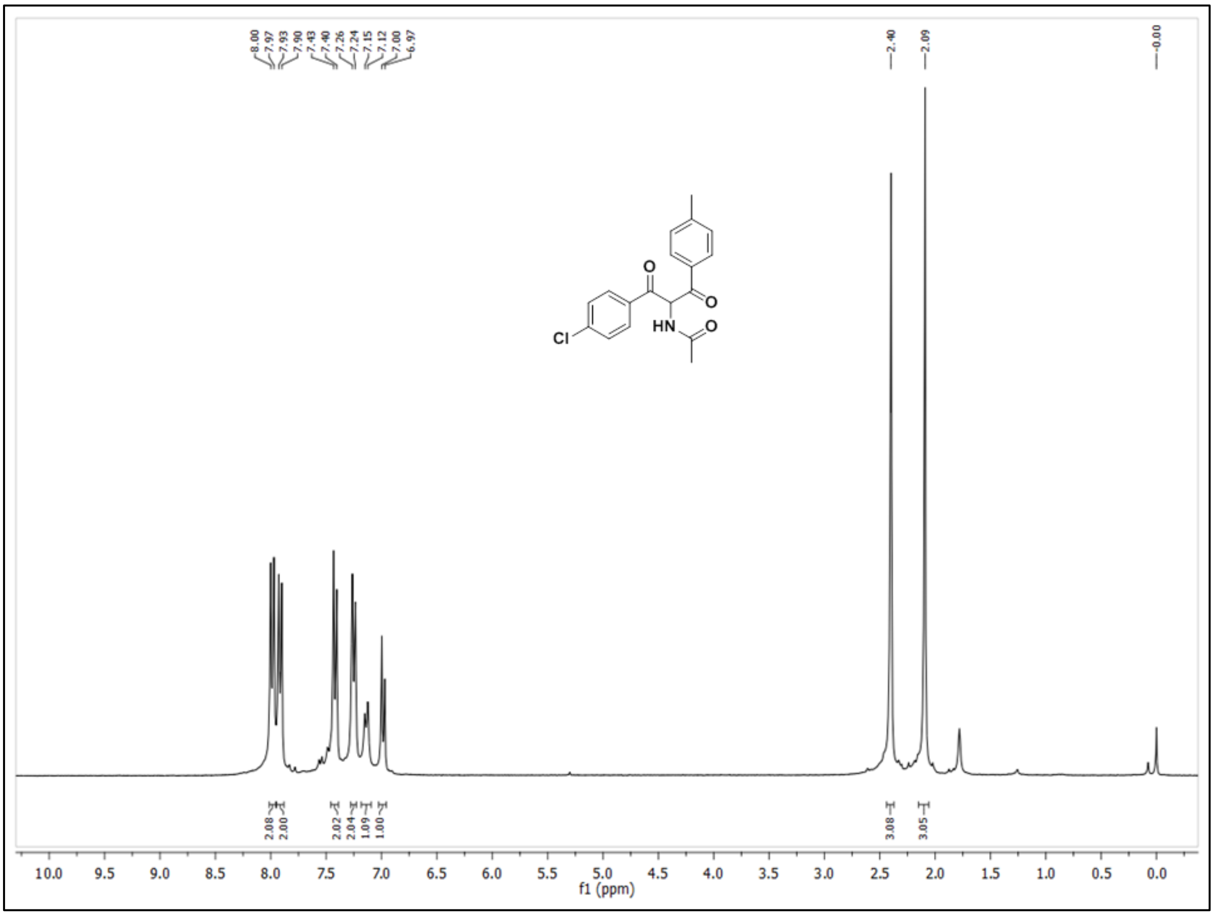

Fig. 10: ^{1}H NMR Spectrum of compound **3d**

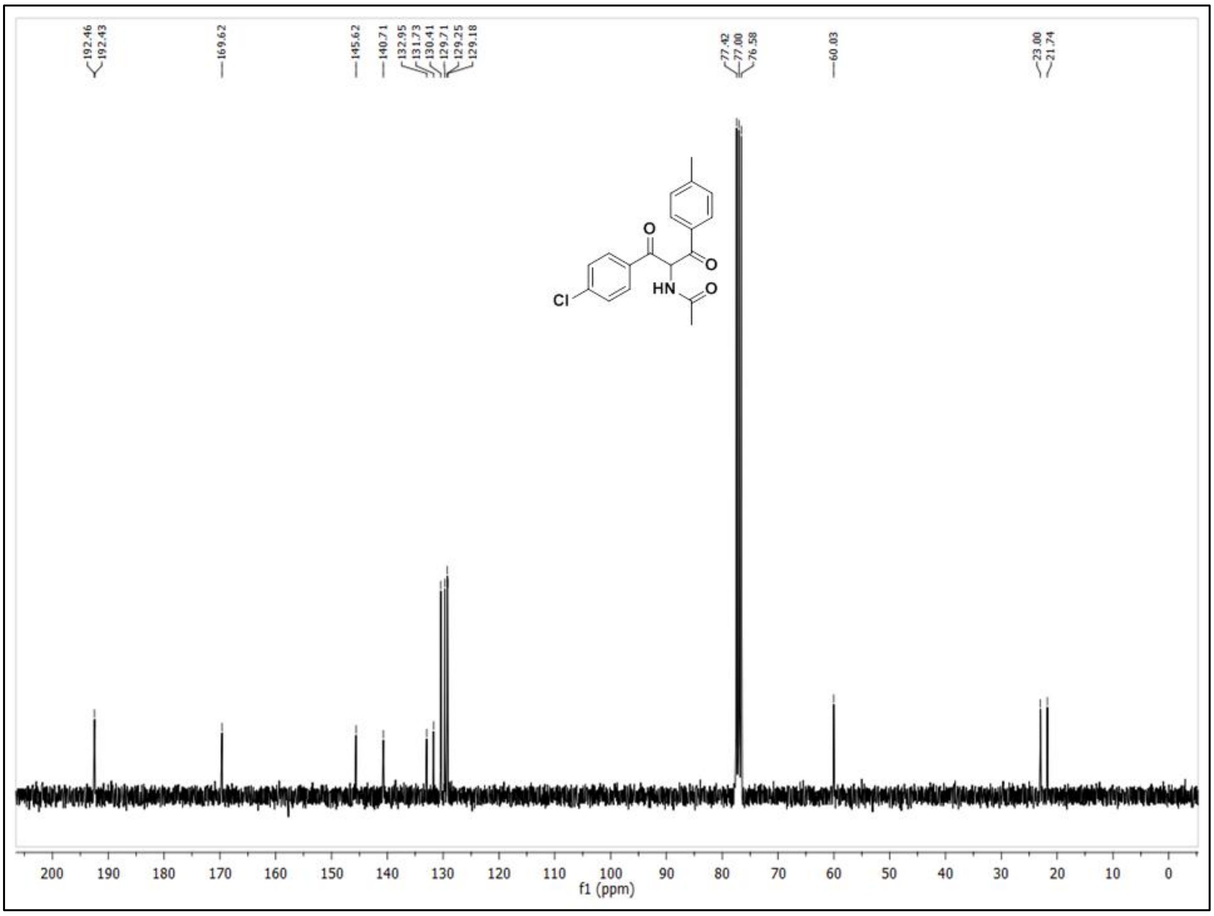

Fig. 11: ^{13}C NMR Spectrum of compound **3d**

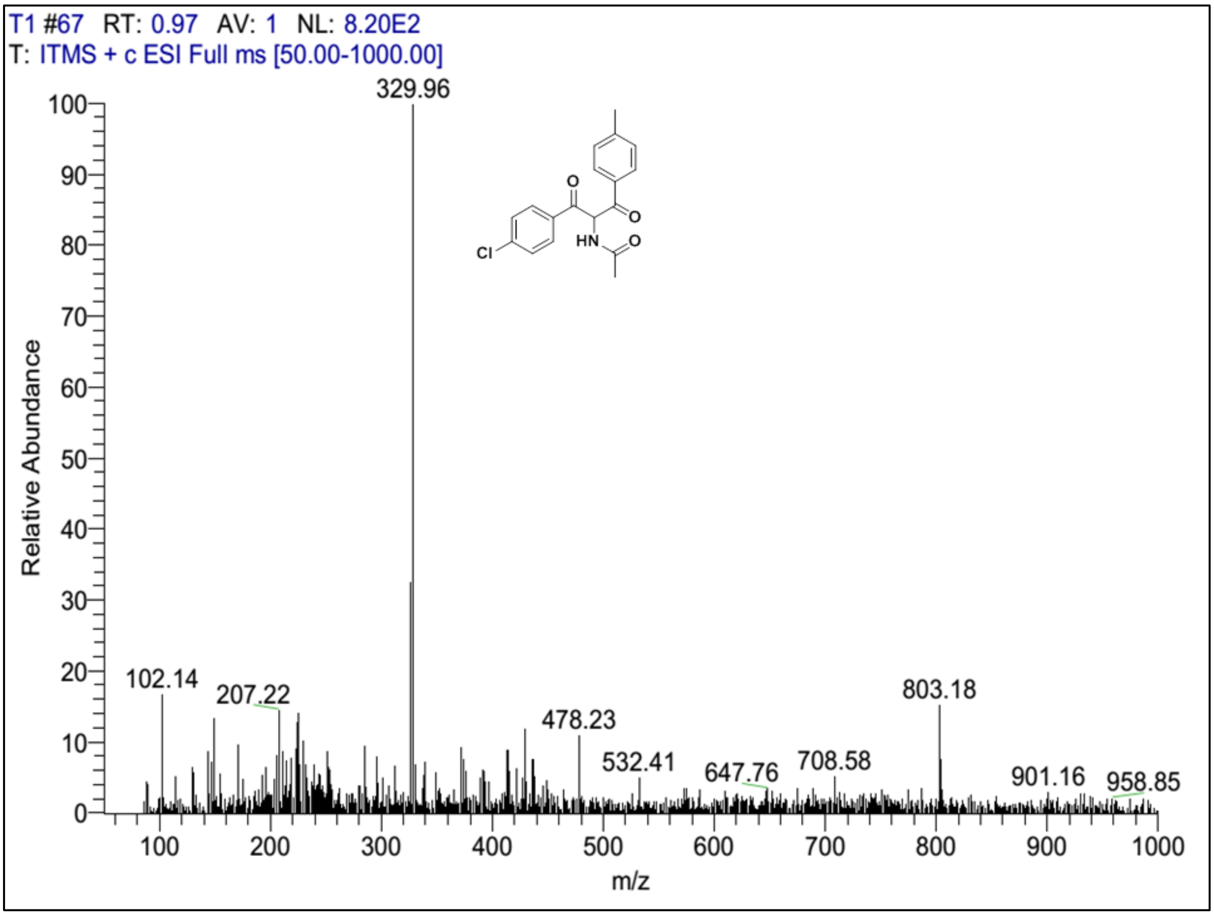

Fig.12 : Mass Spectrum of compound **3d**

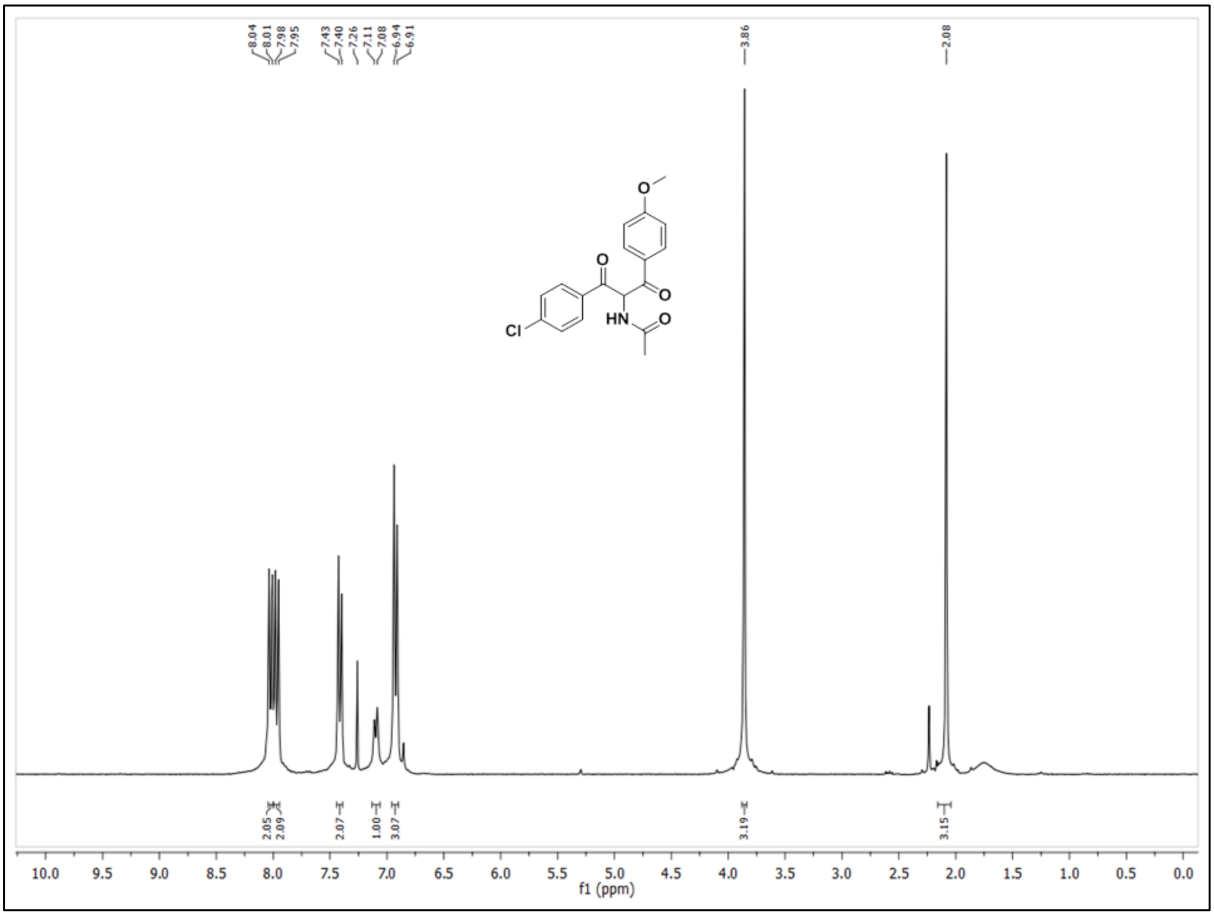

Fig. 13: ^{1}H NMR Spectrum of compound **3e**

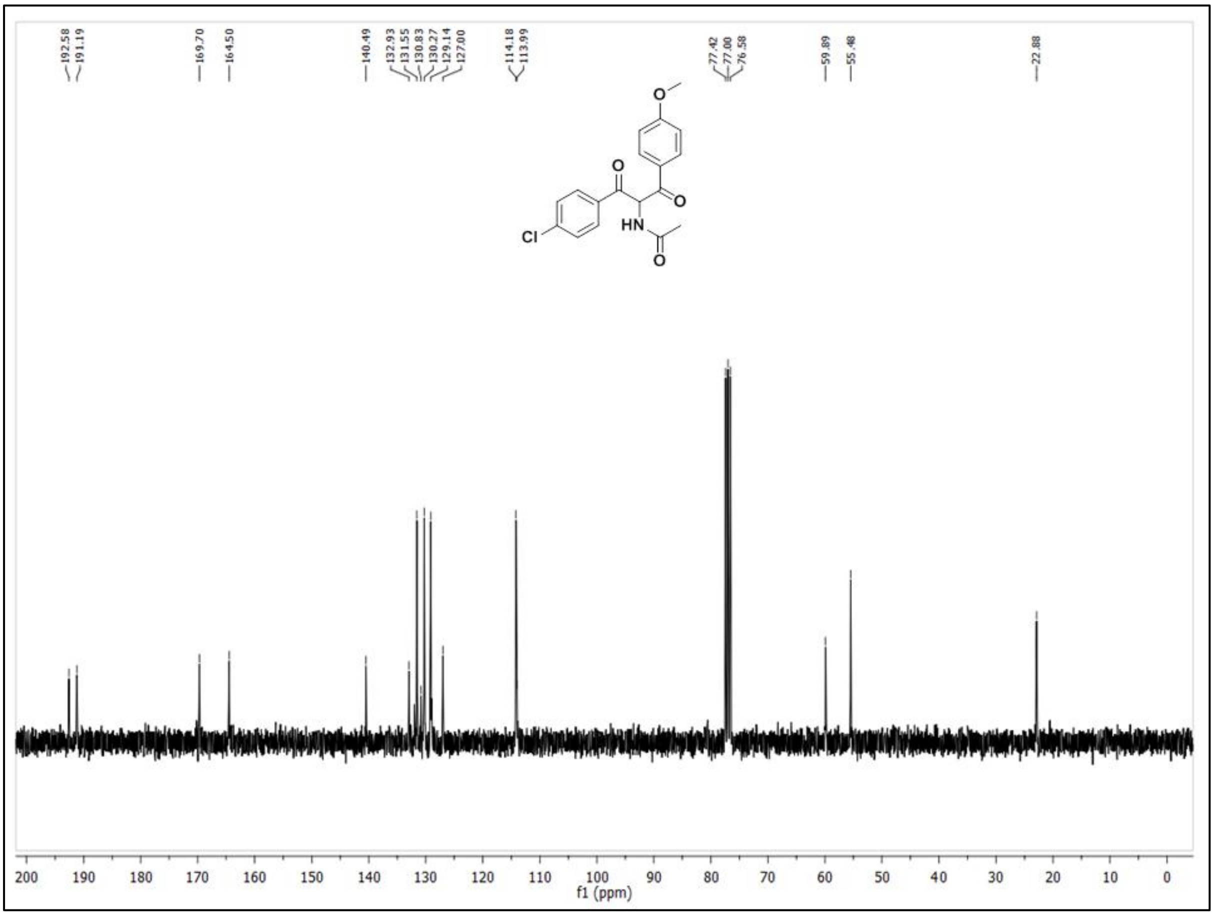

Fig. 14: ^{13}C NMR Spectrum of compound **3e**

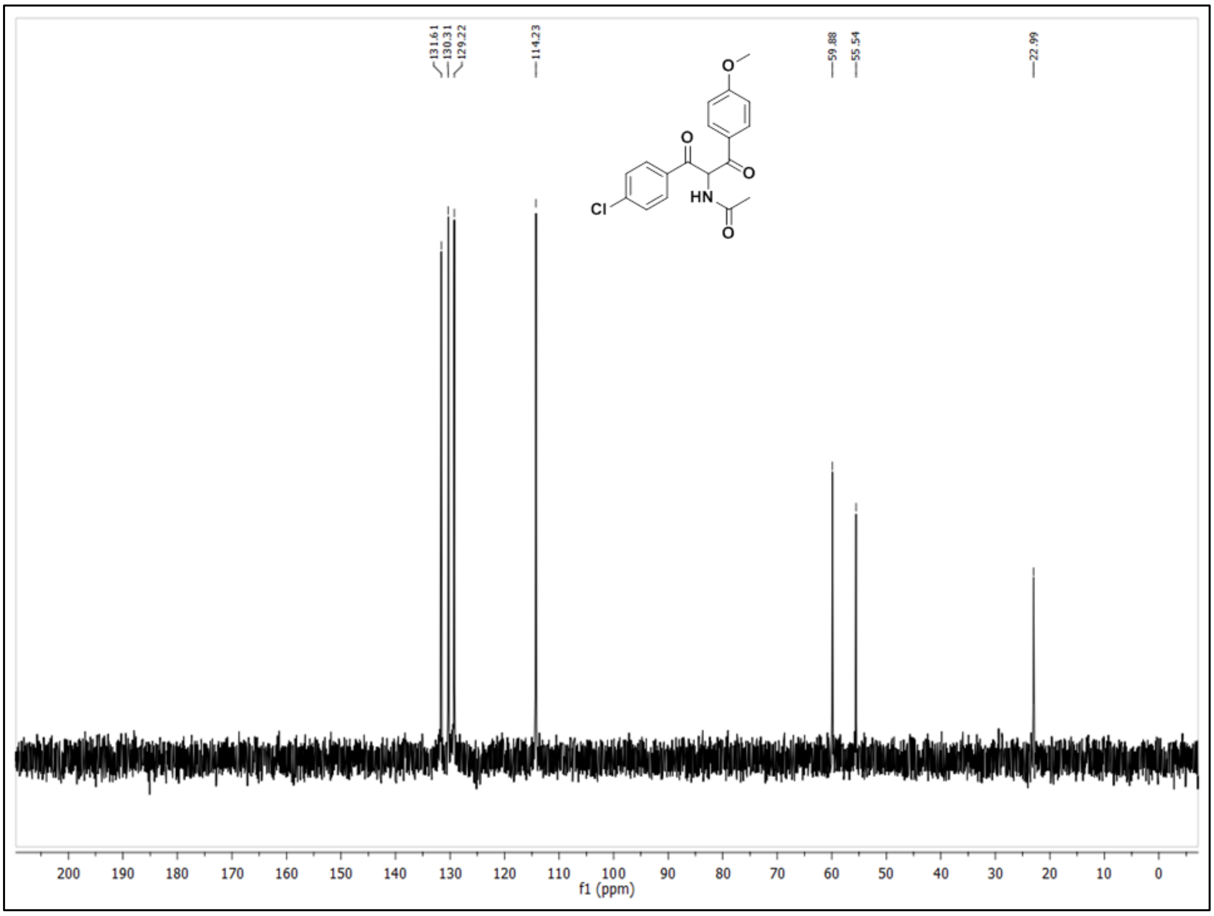

Fig. 15: DEPT-135 Spectrum of compound **3e**

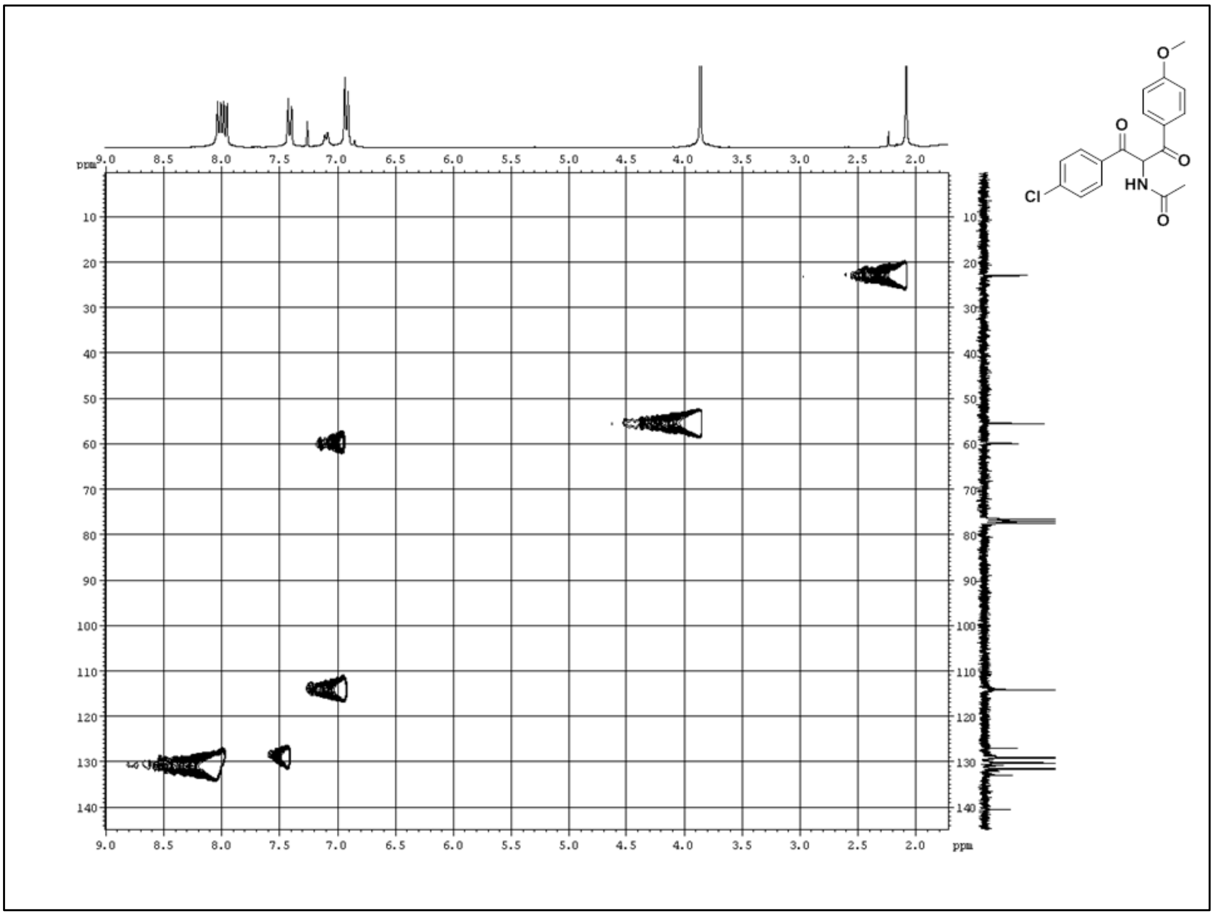

Fig. 16: C,H COSY Spectrum of compound **3e**

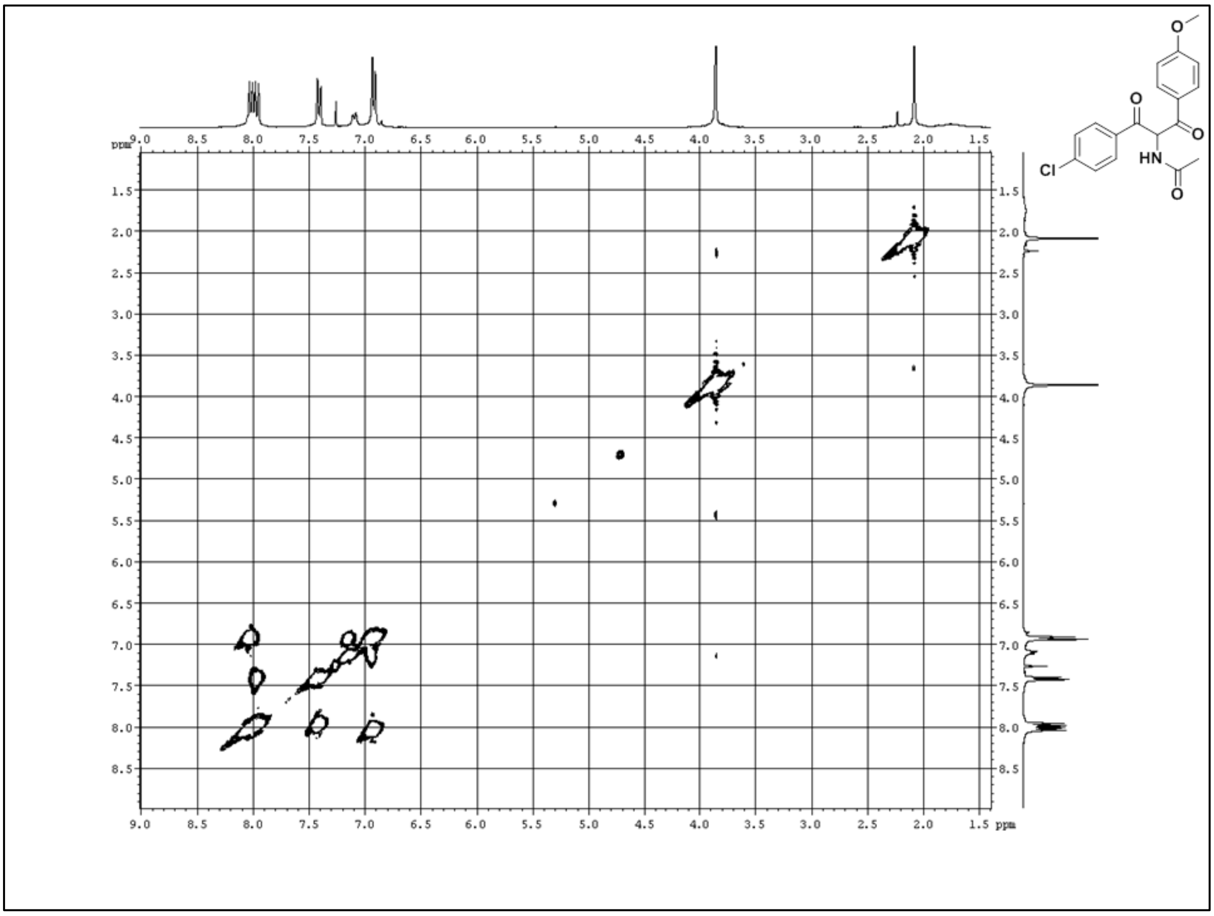

Fig. 17: H,H COSY Spectrum of compound **3e**

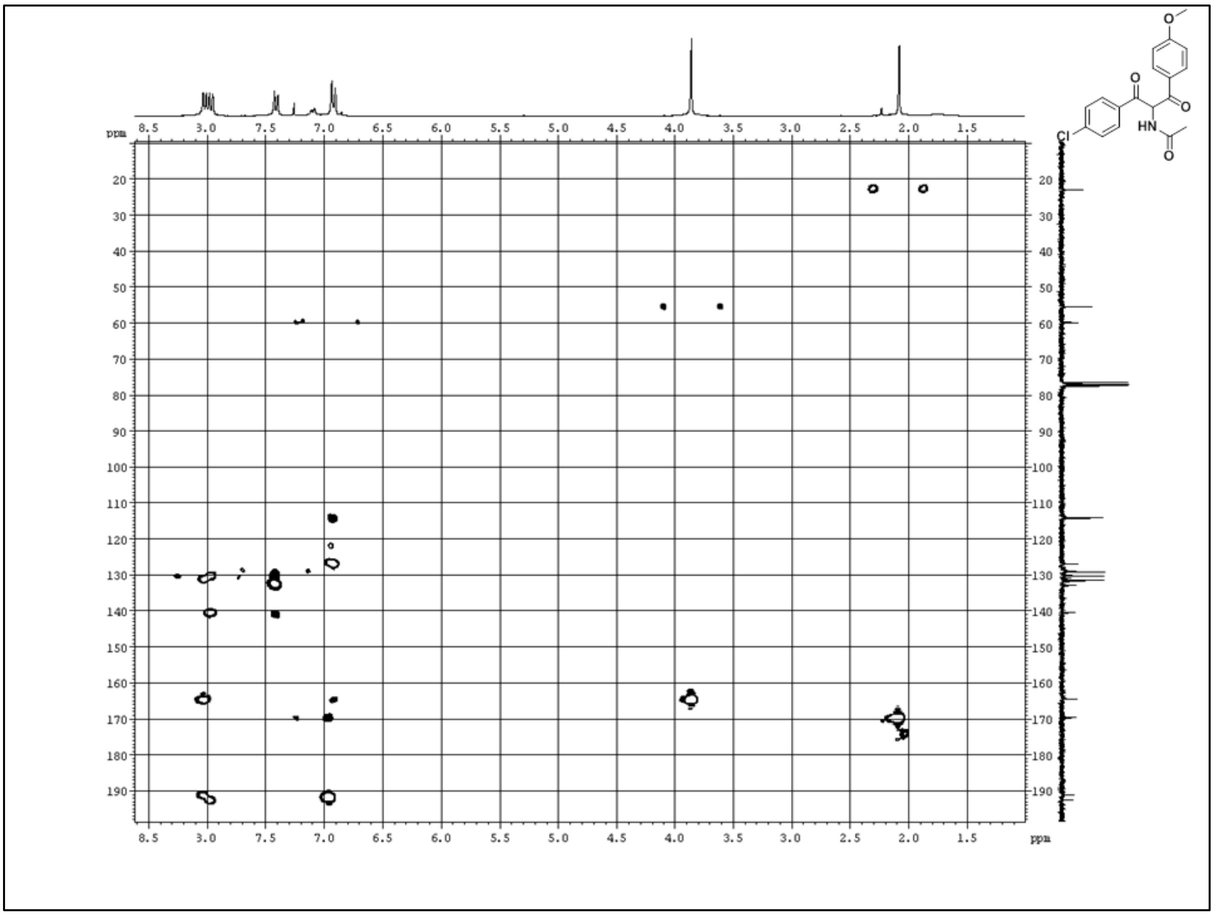

Fig. 18: HMBC Spectrum of compound **3e**

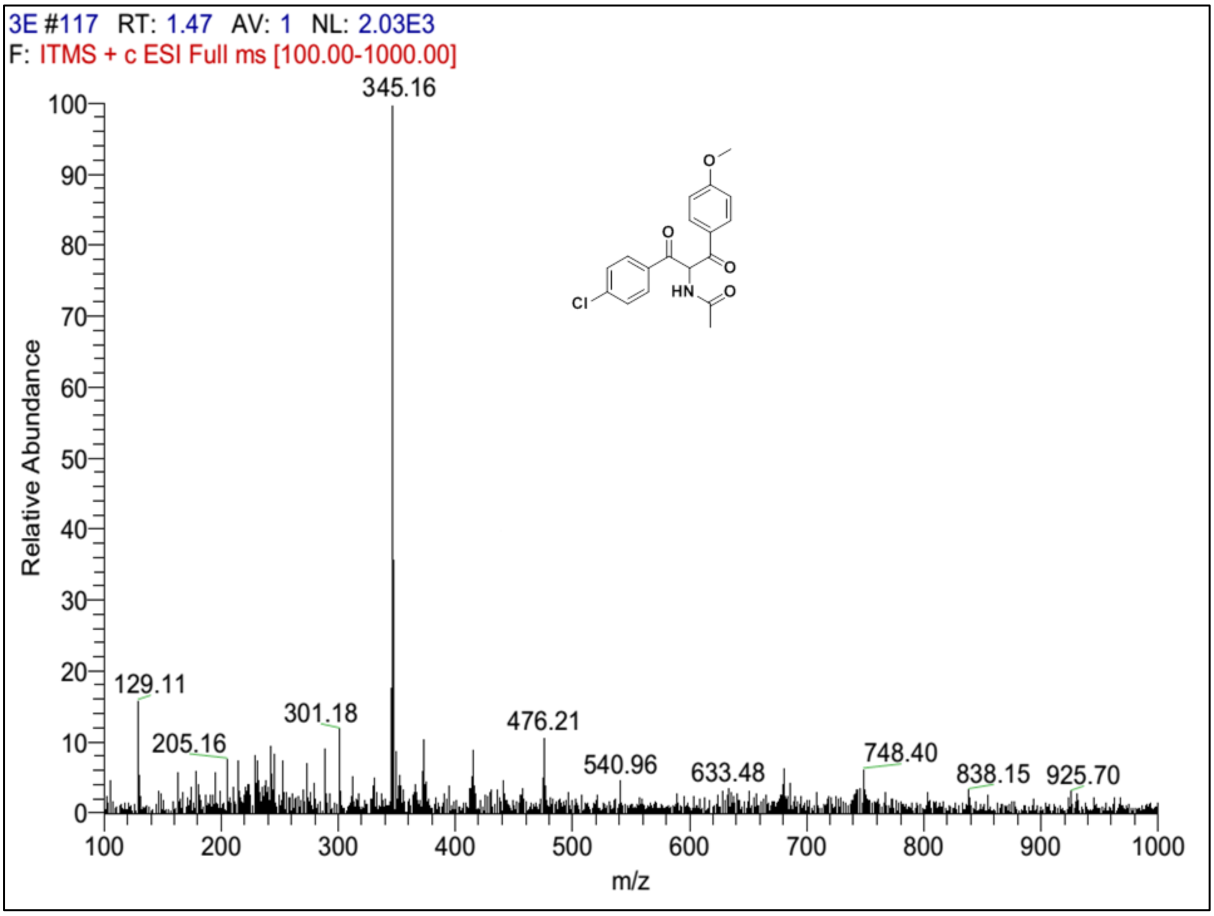

Fig. 19: Mass Spectrum of compound **3e**

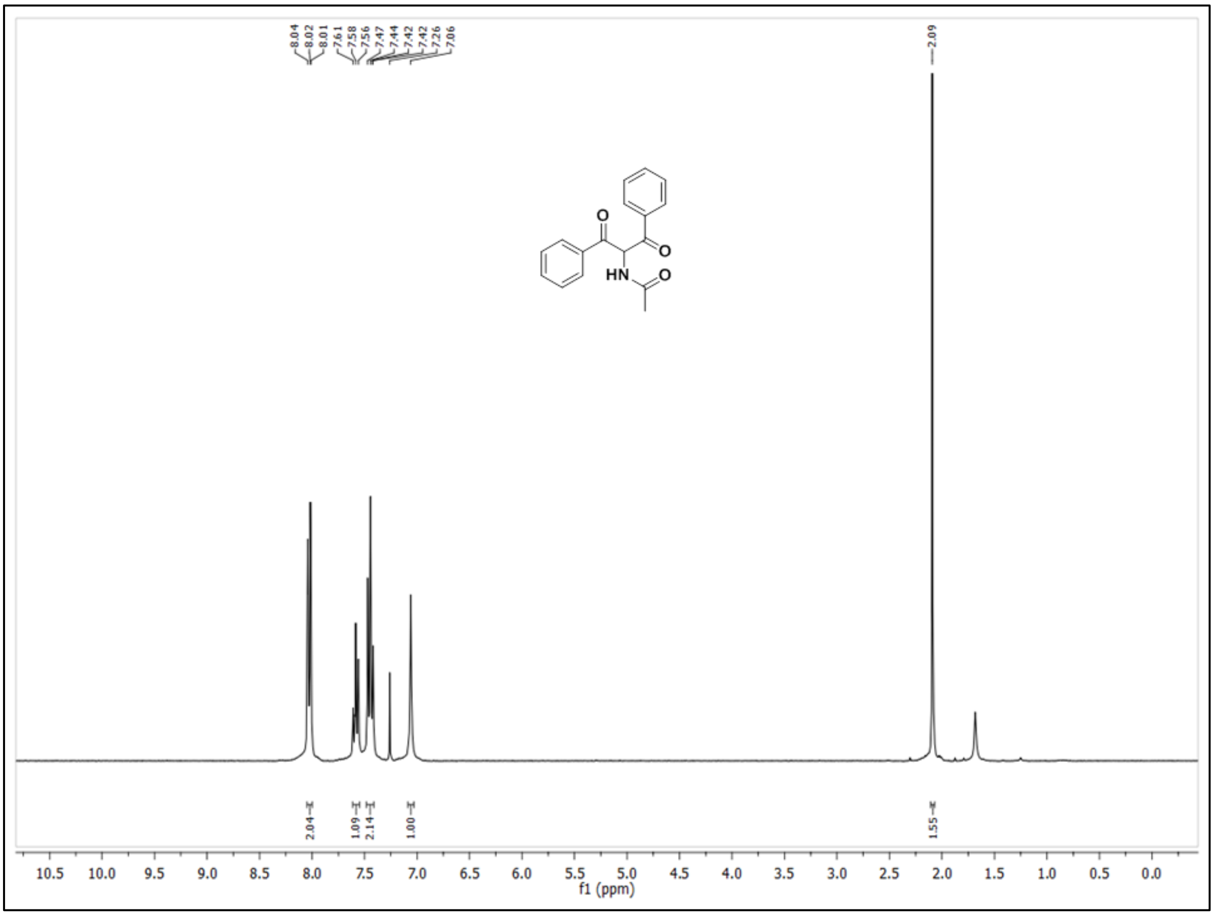

Fig. 20: ^{1}H NMR Spectrum of compound **3f**

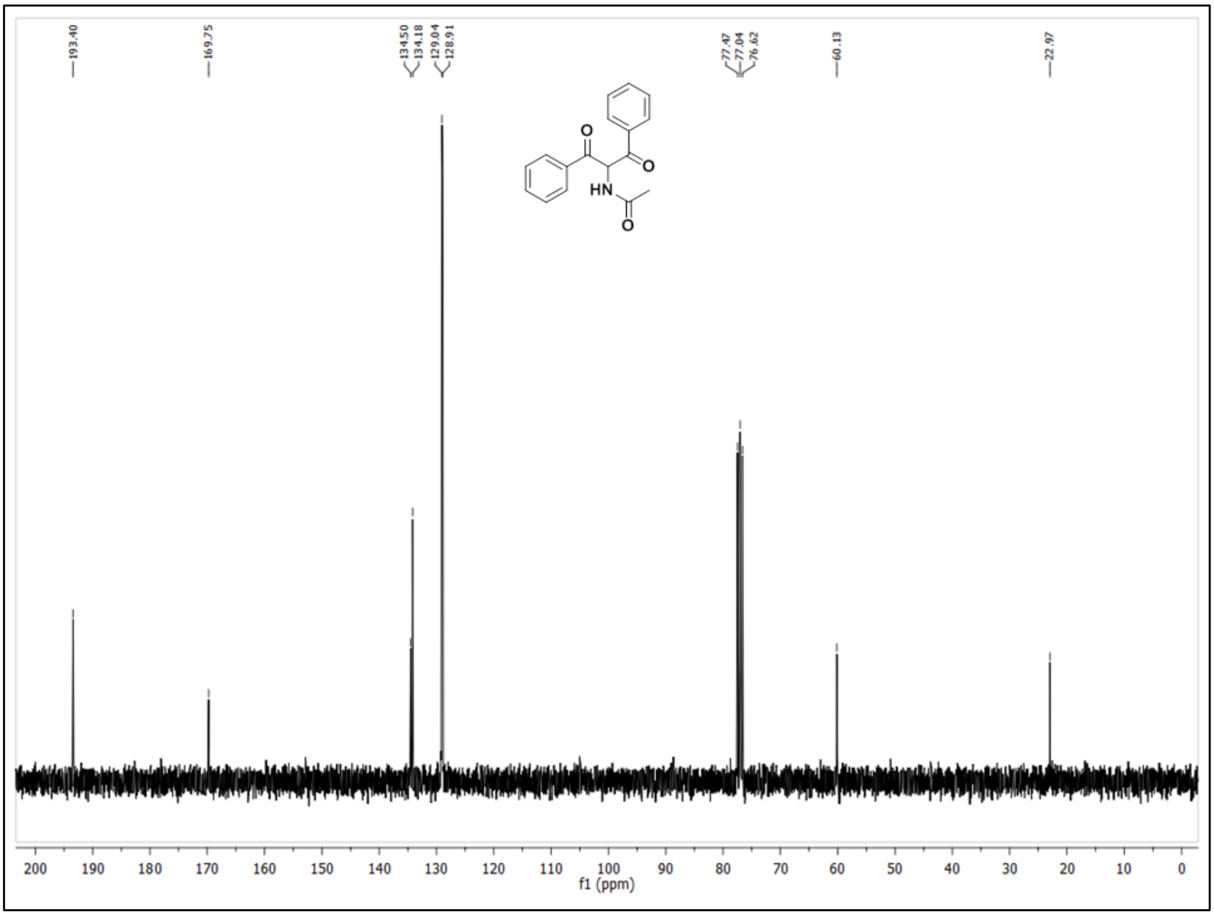

Fig. 21: ^{13}C NMR Spectrum of compound **3f**

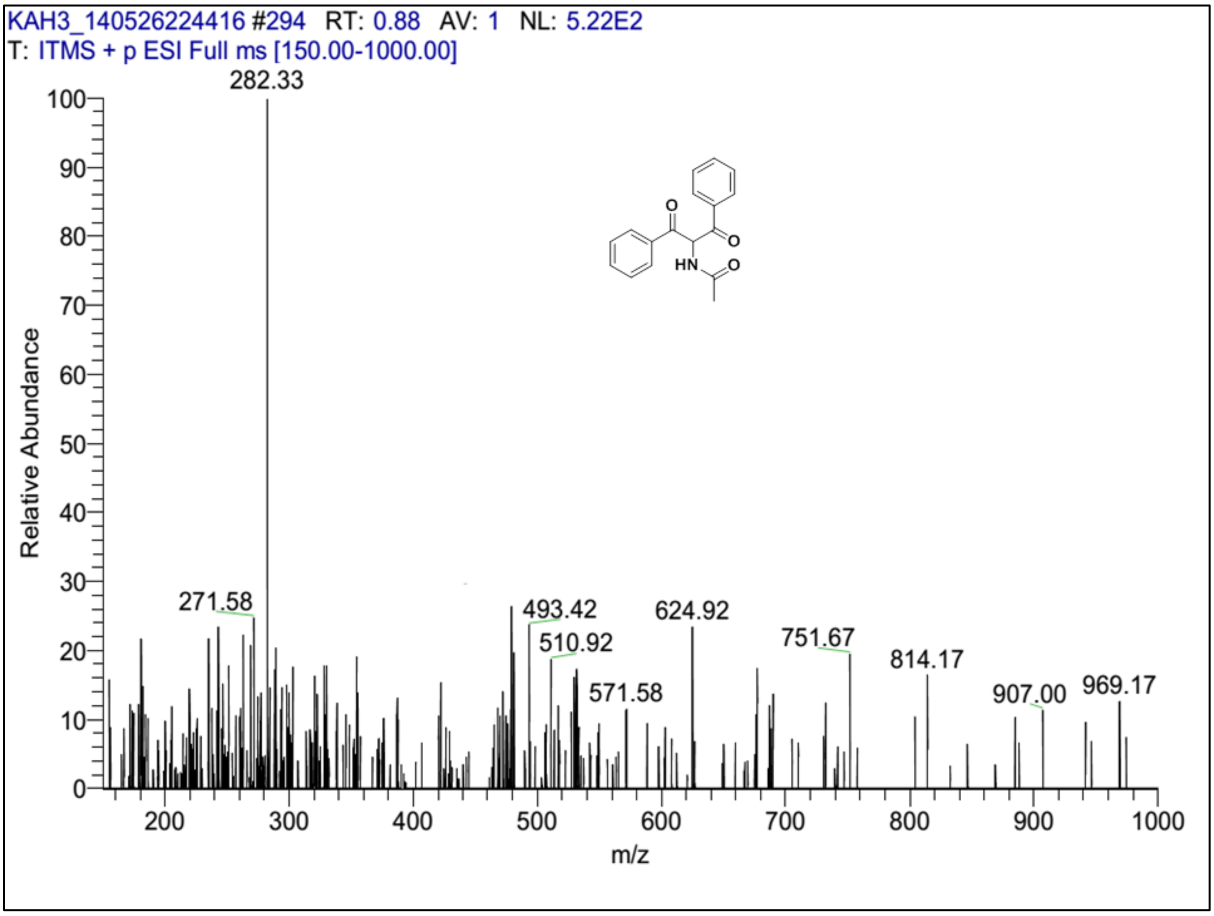

Fig. 22: Mass Spectrum of compound **3f**

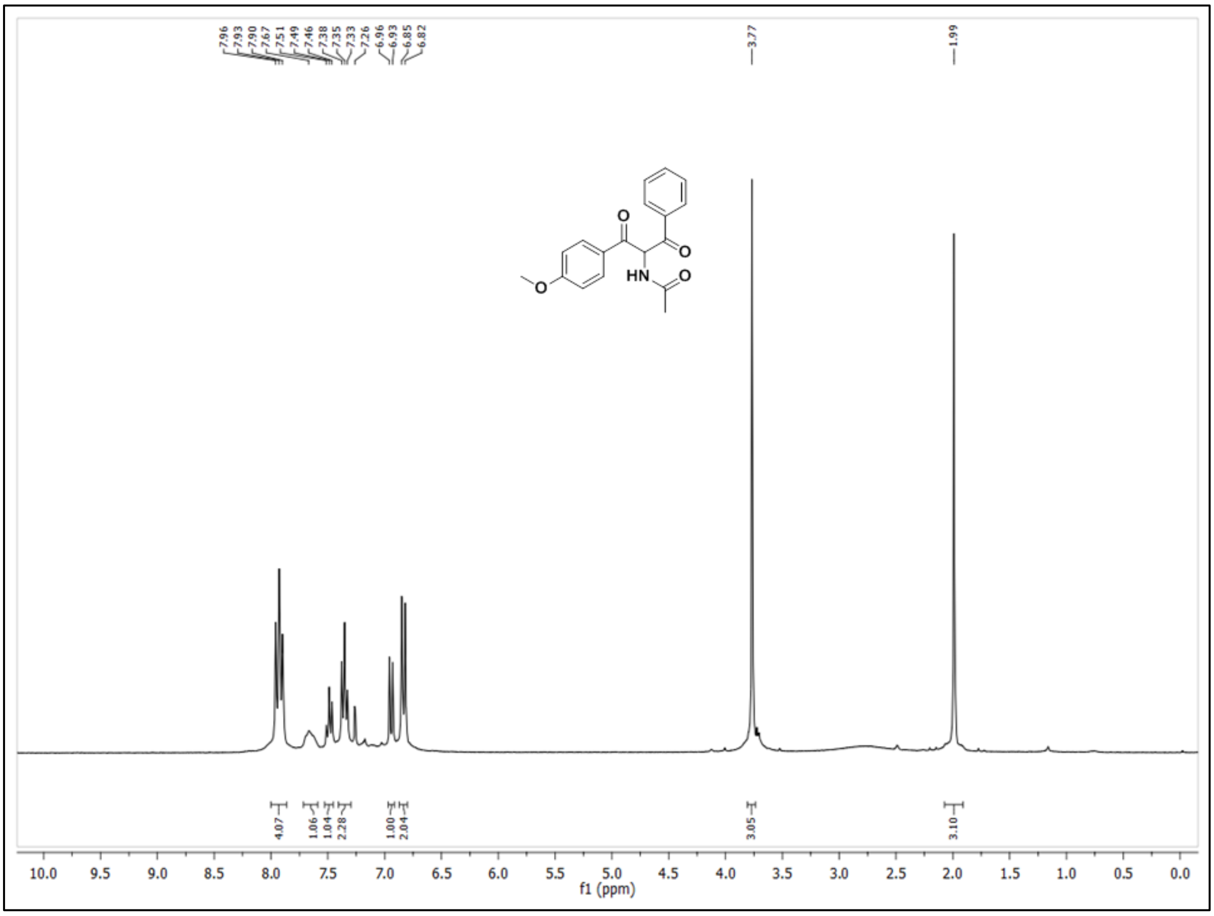

Fig. 23: ^{1}H NMR Spectrum of compound **3g**

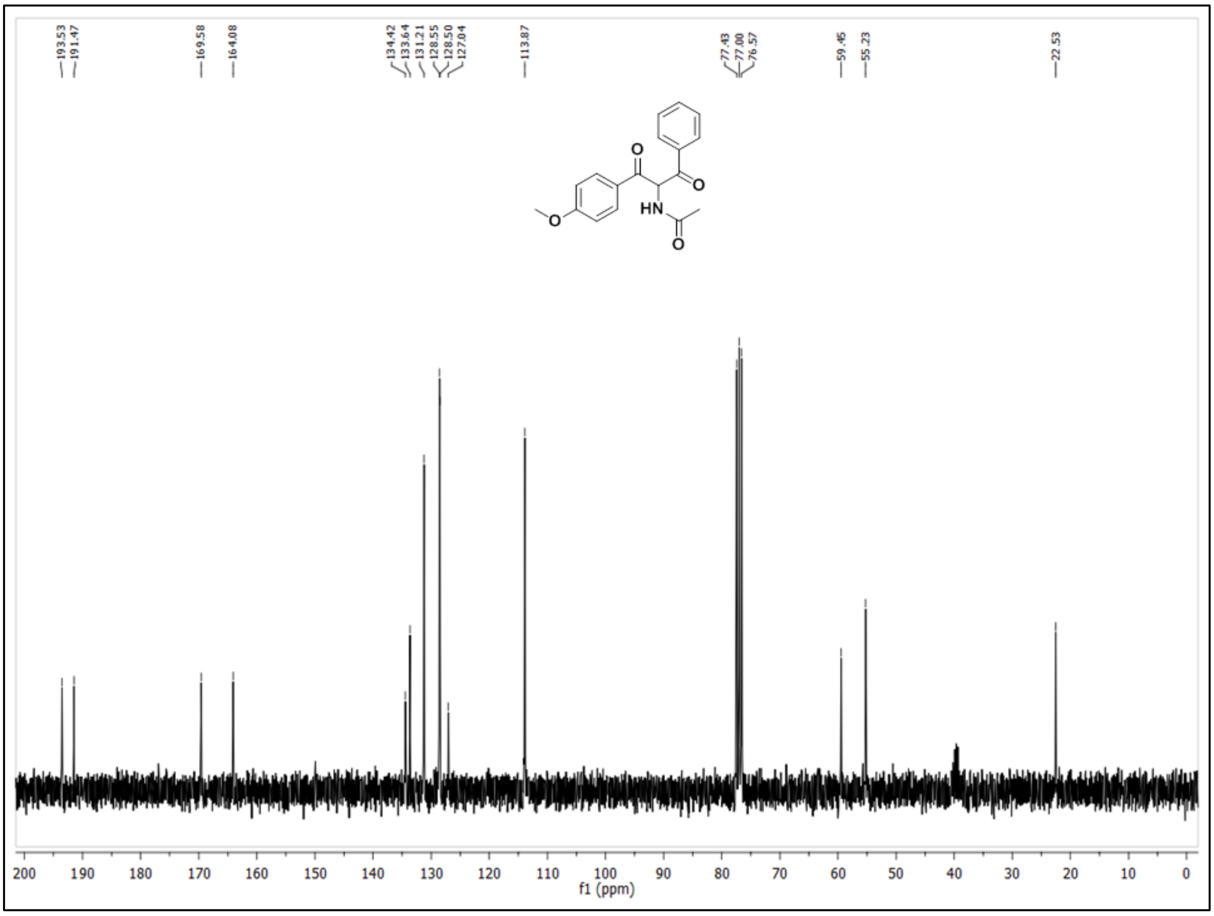

Fig. 24: ^{13}C NMR Spectrum of compound **3g**

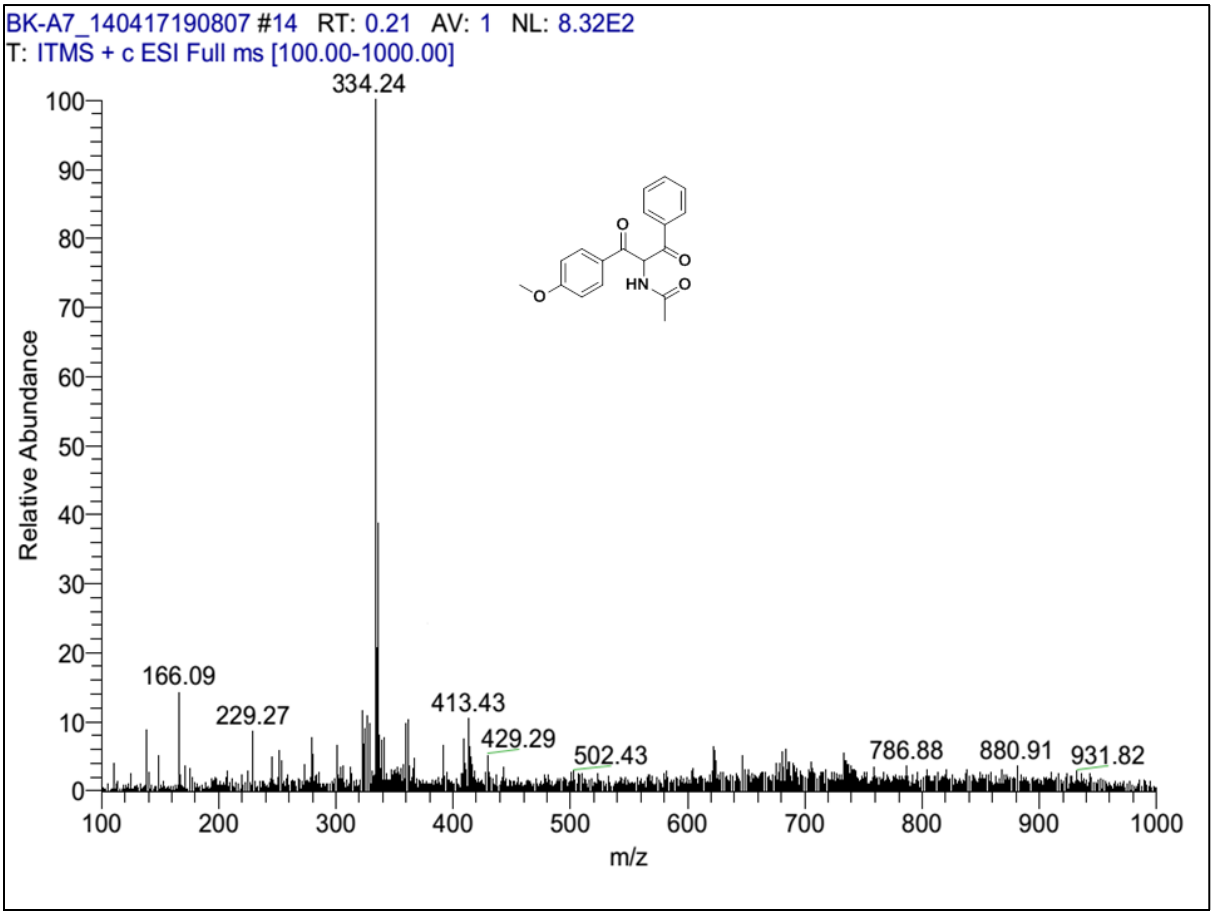

Fig. 25: Mass Spectrum of compound **3g**

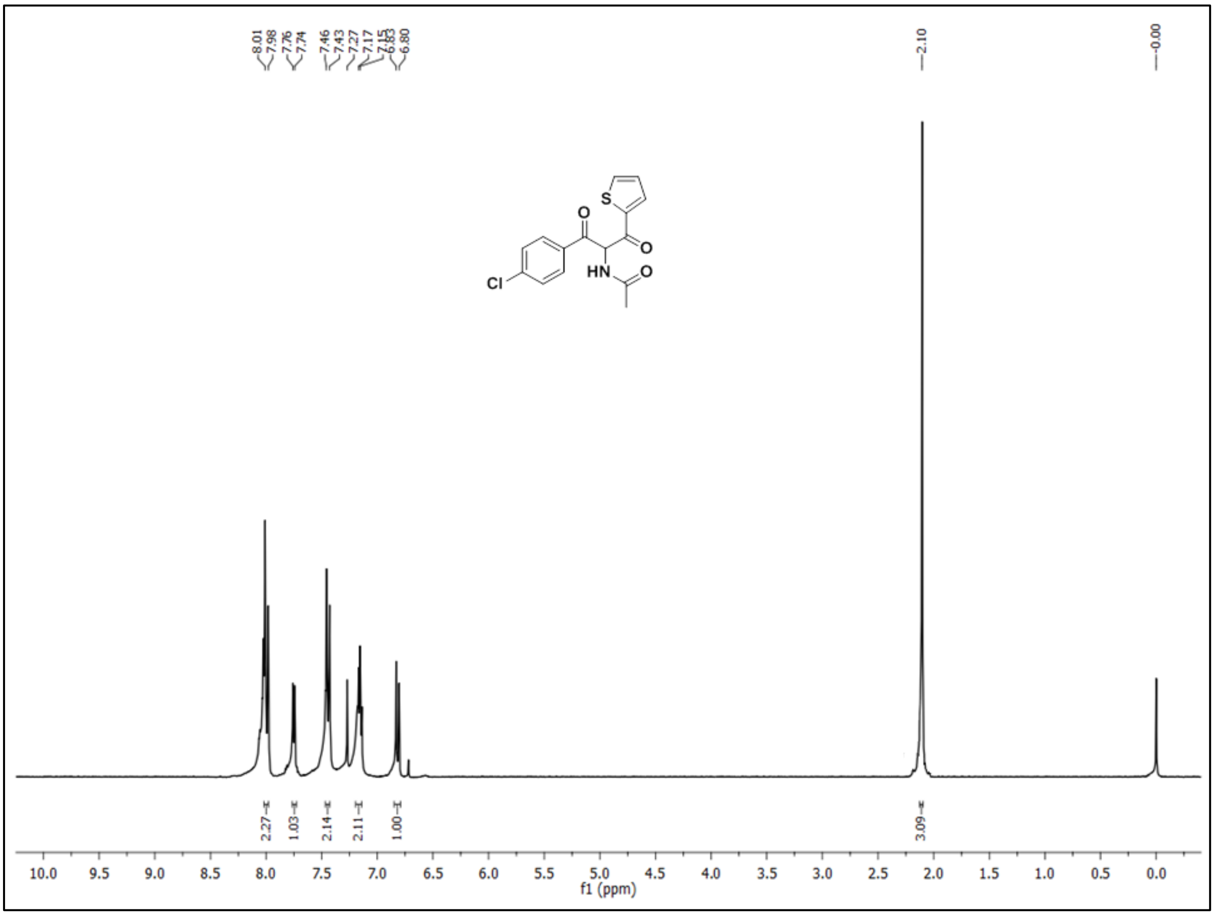

Fig. 26: ^{1}H NMR Spectrum of compound **3h**

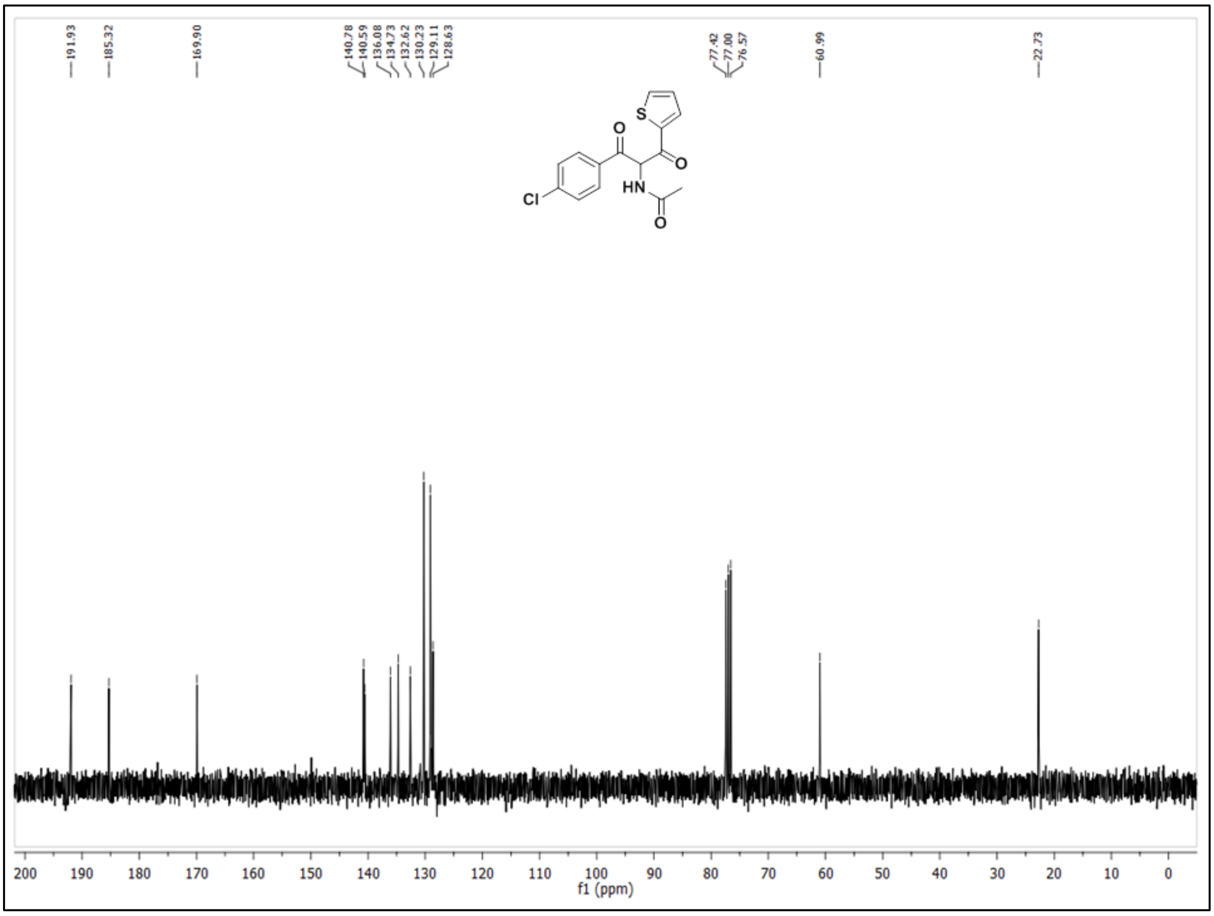

Fig. 26: ^{13}C NMR Spectrum of compound **3h**

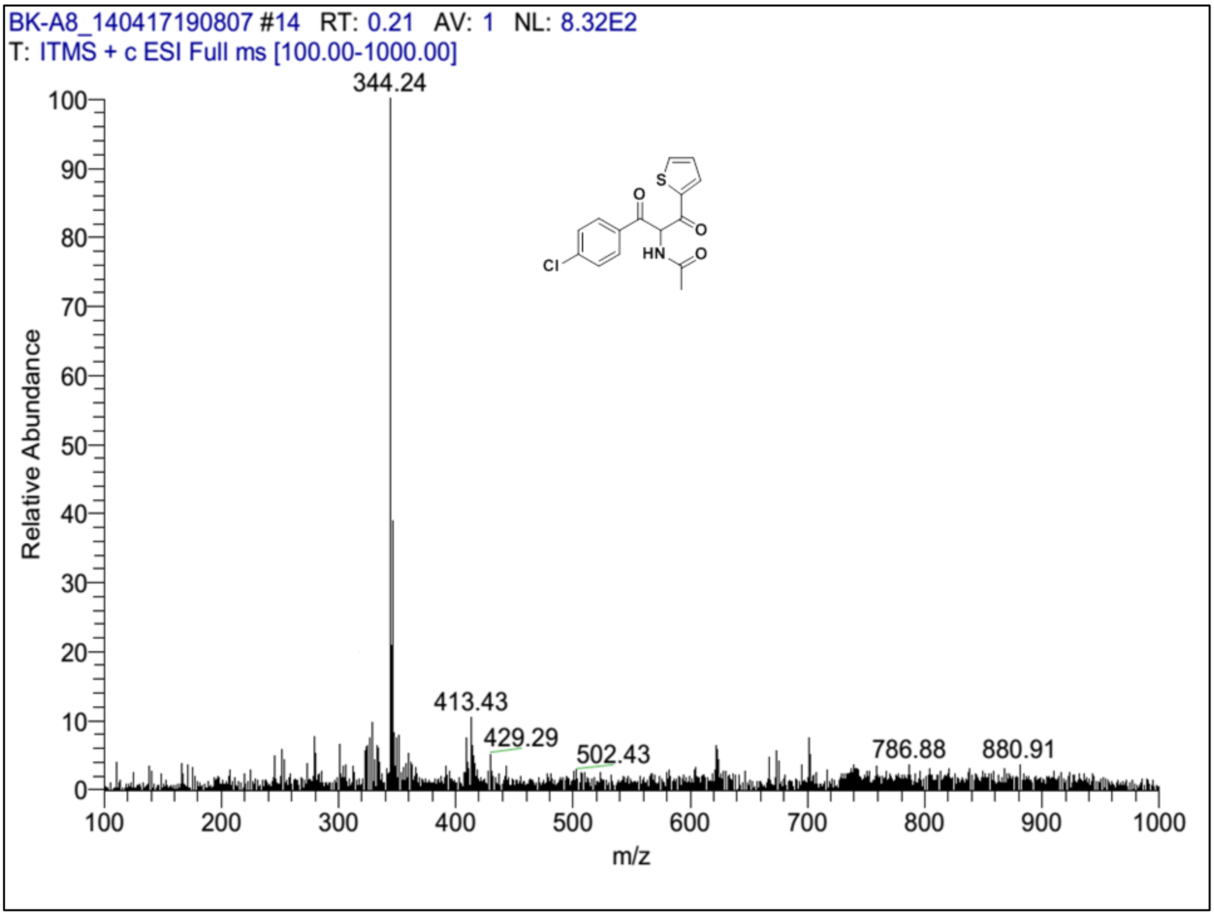

Fig. 27: Mass Spectrum of compound **3h**

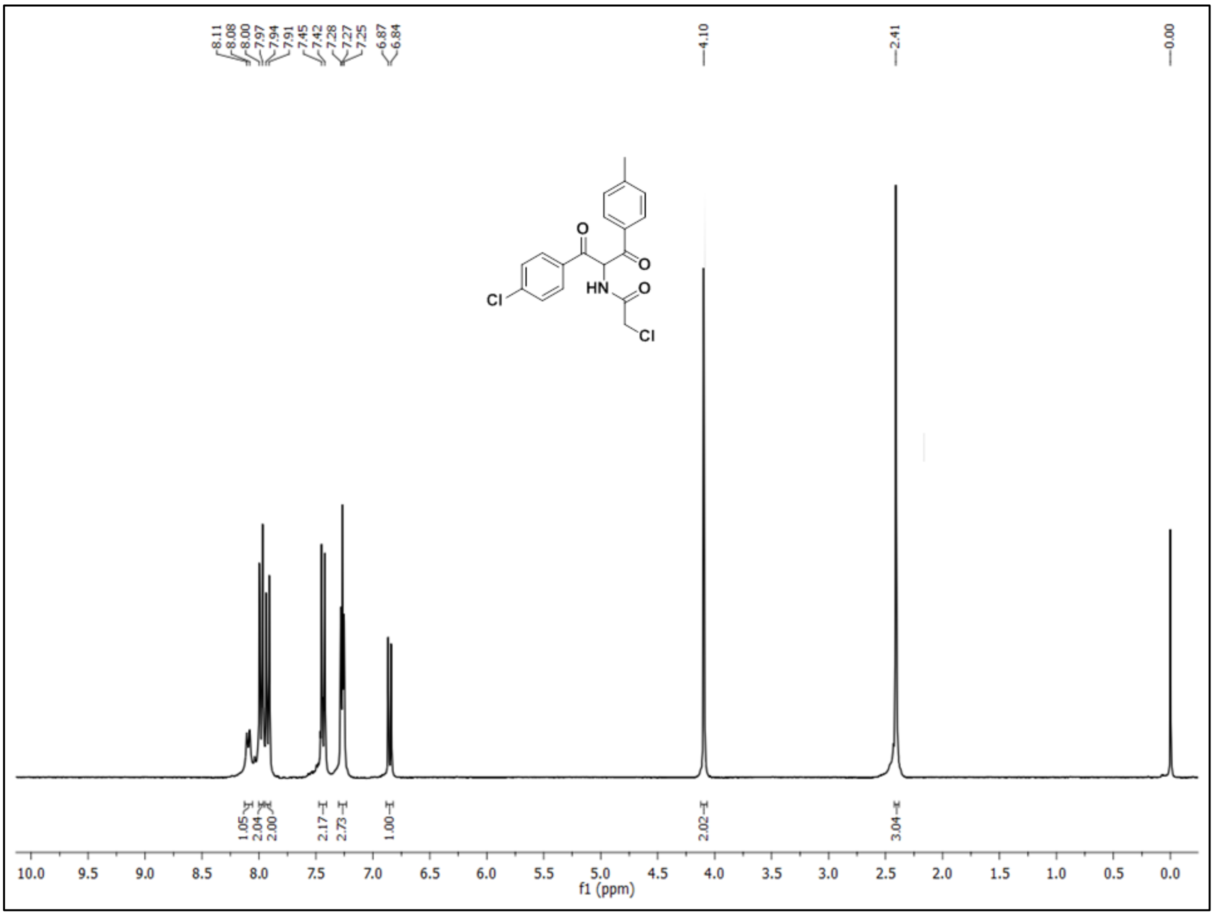

Fig. 28: ^{1}H NMR Spectrum of compound **3i**

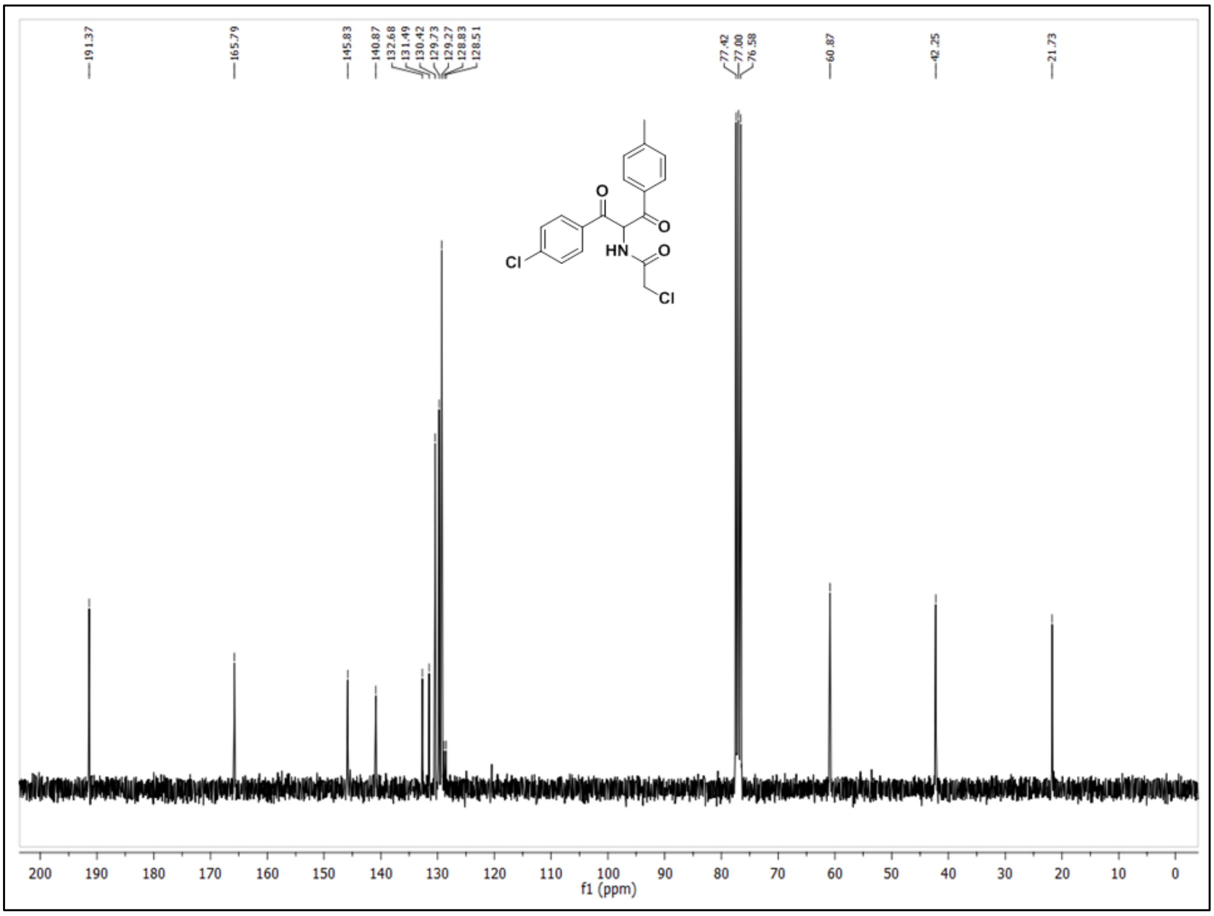

Fig. 29: ^{13}C NMR Spectrum of compound **3i**

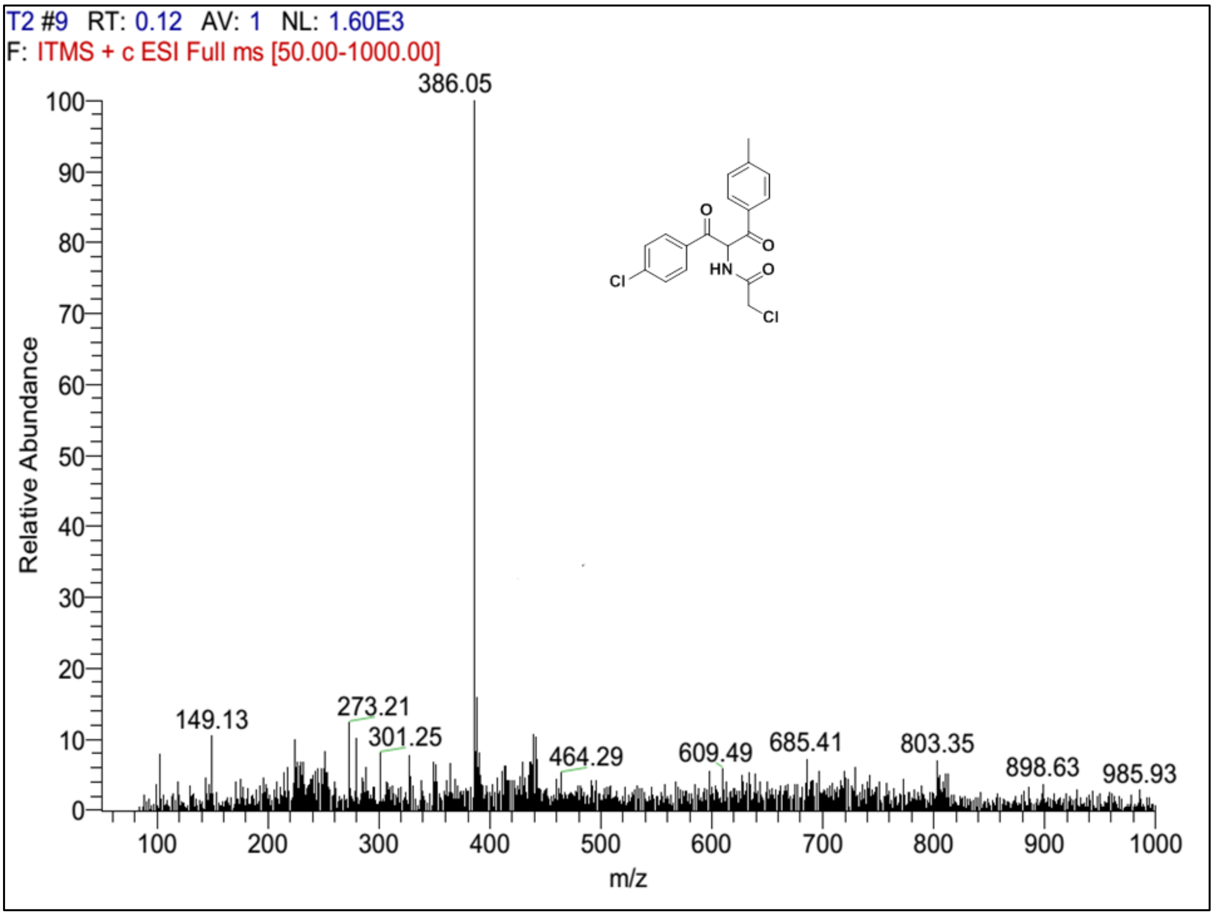

Fig. 30: Mass Spectrum of compound **3i**

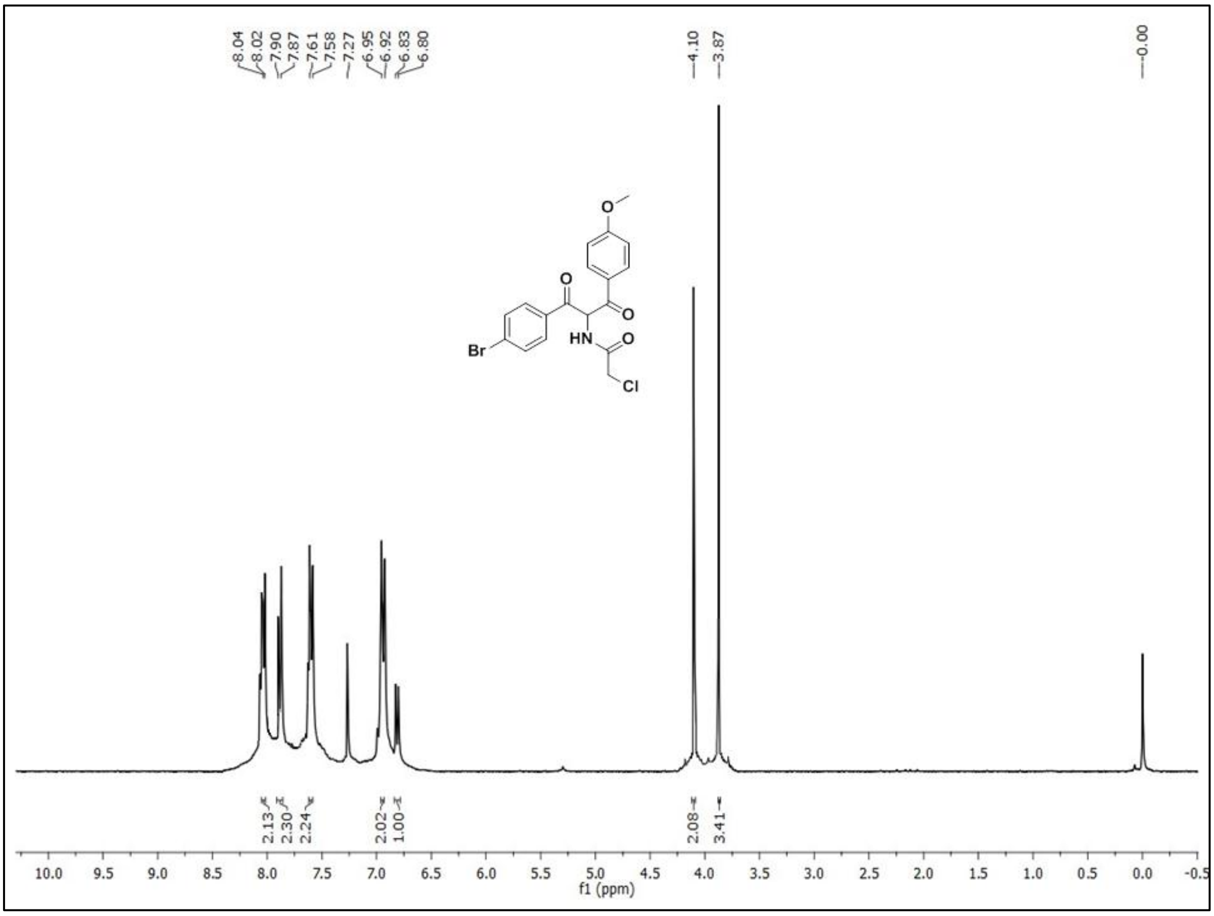

Fig. 31: ^{1}H NMR Spectrum of compound **3j**

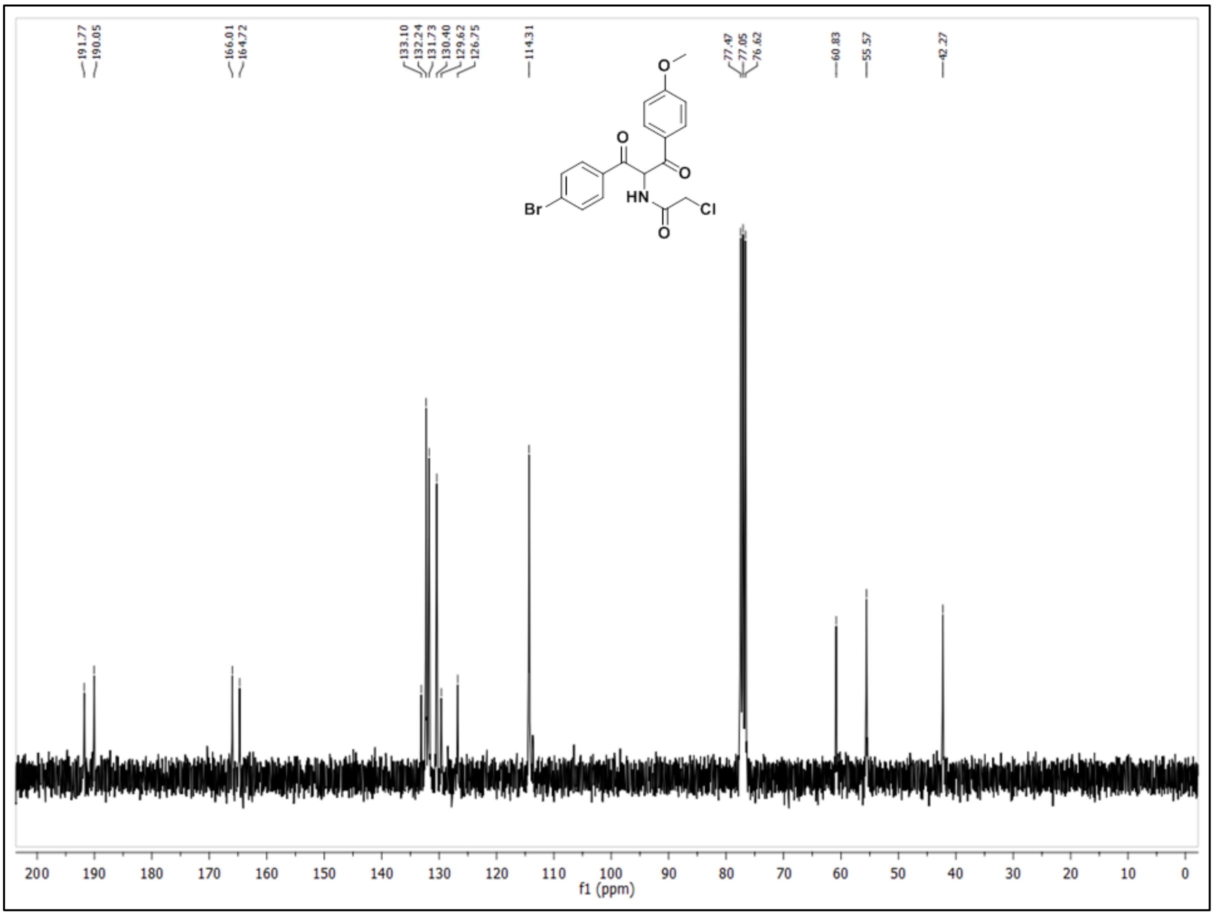

Fig. 32: ^{13}C NMR Spectrum of compound **3j**

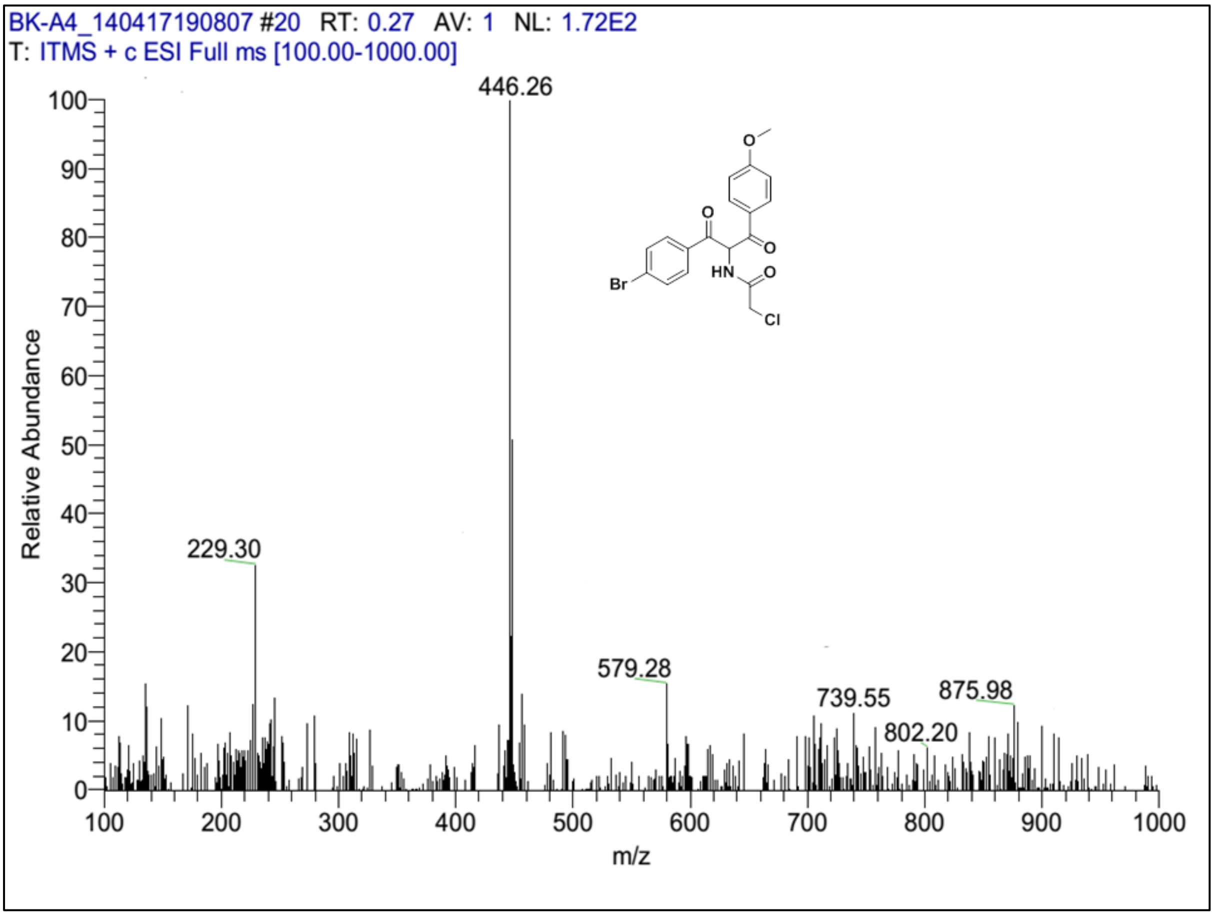

Fig. 33: Mass Spectrum of compound **3j**

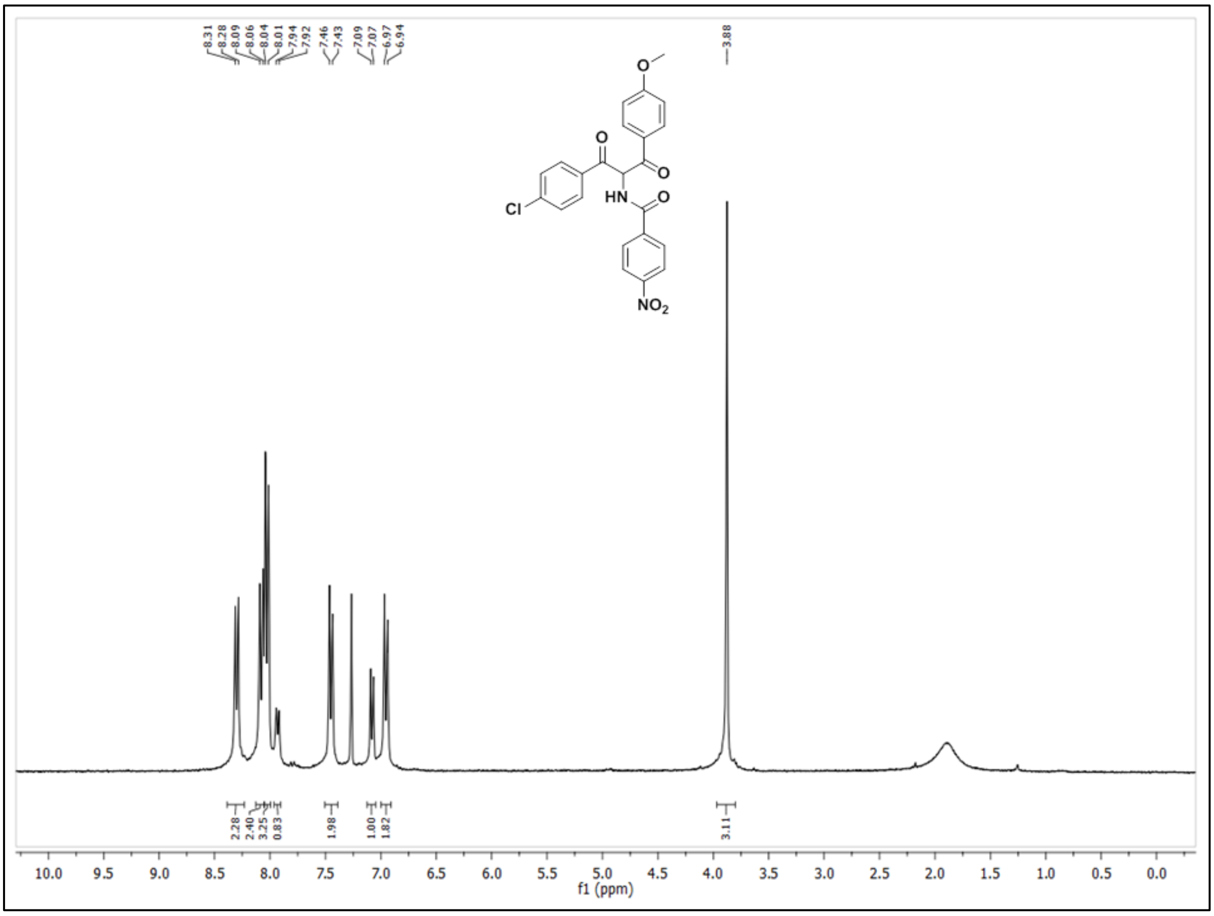

Fig. 34: ^{1}H NMR Spectrum of compound **3k**

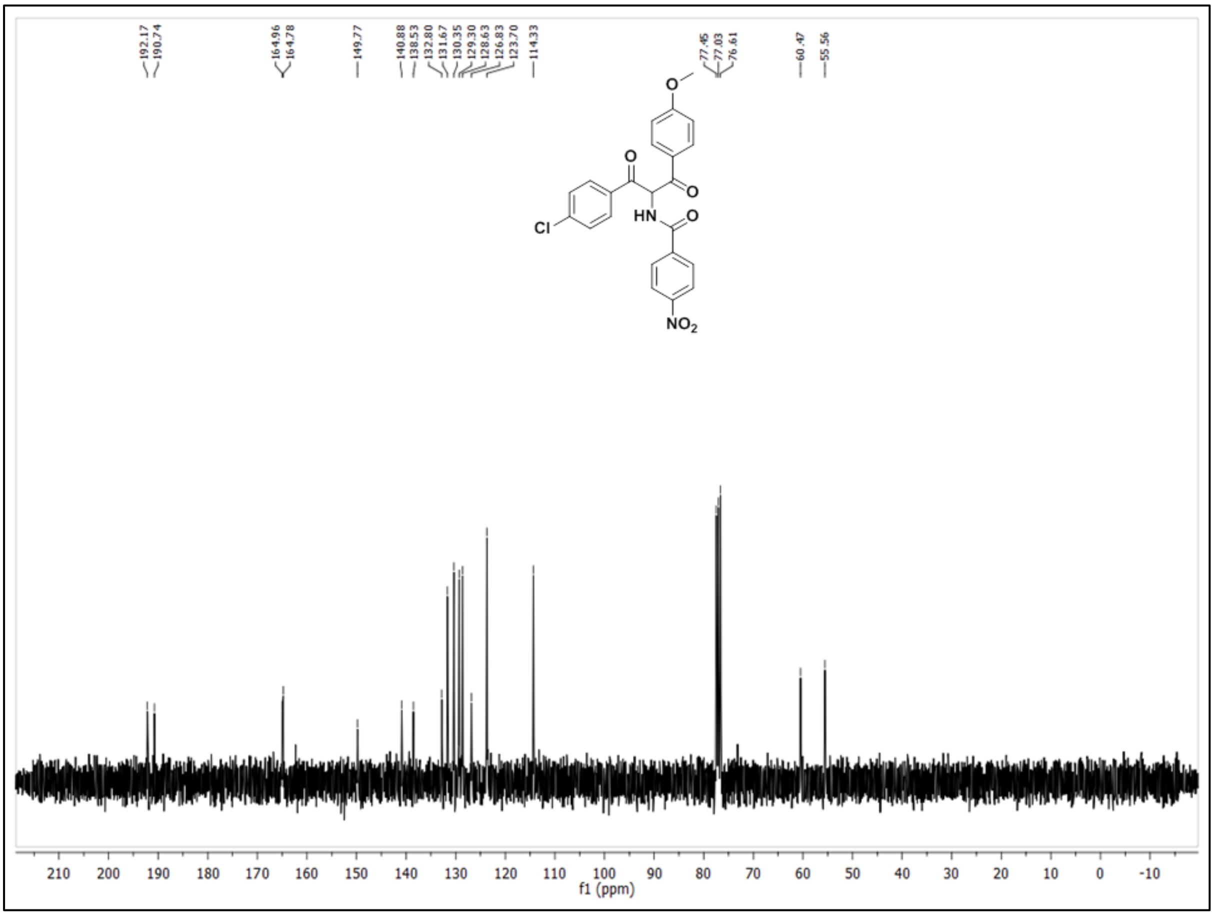

Fig. 35: ^{13}C NMR Spectrum of compound **3k**

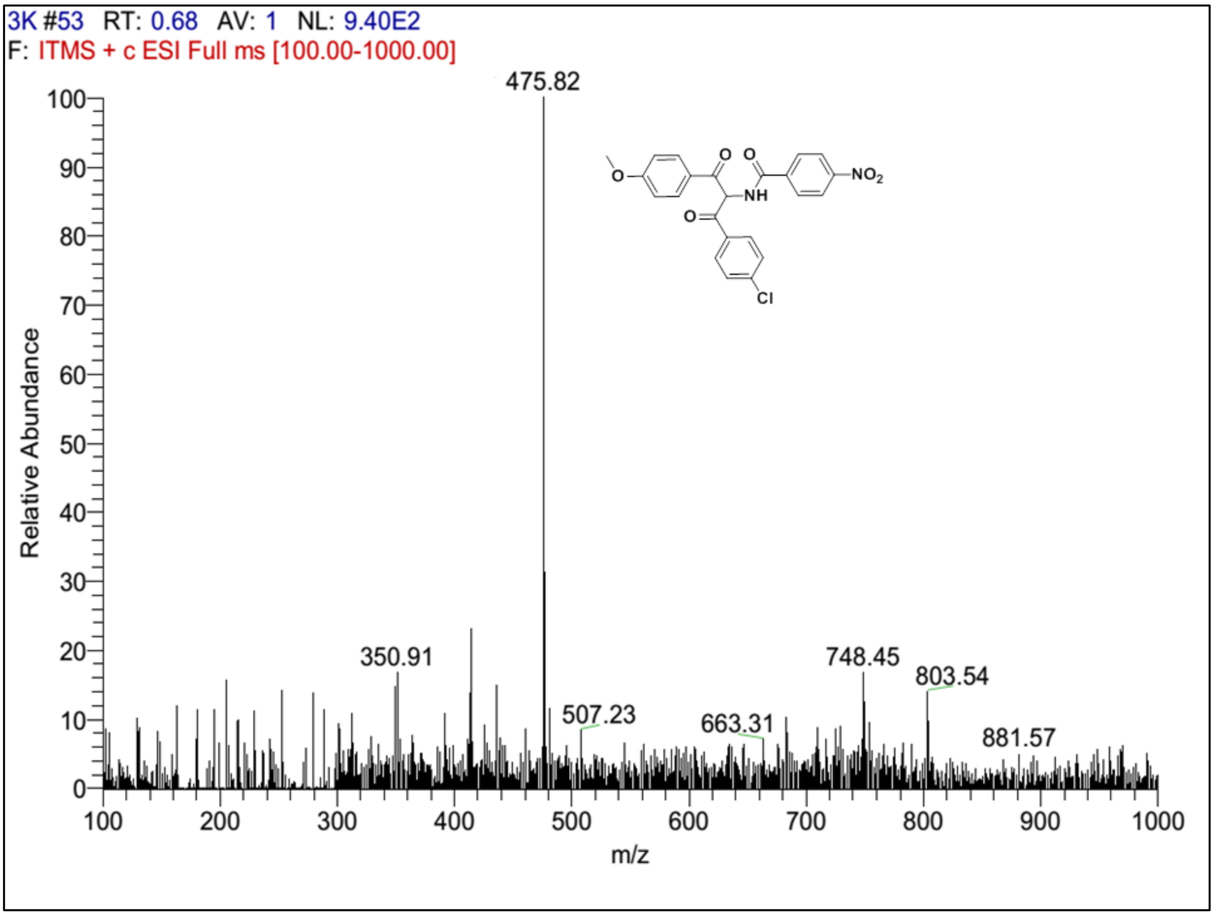

Fig. 36: Mass Spectrum of compound **3k**

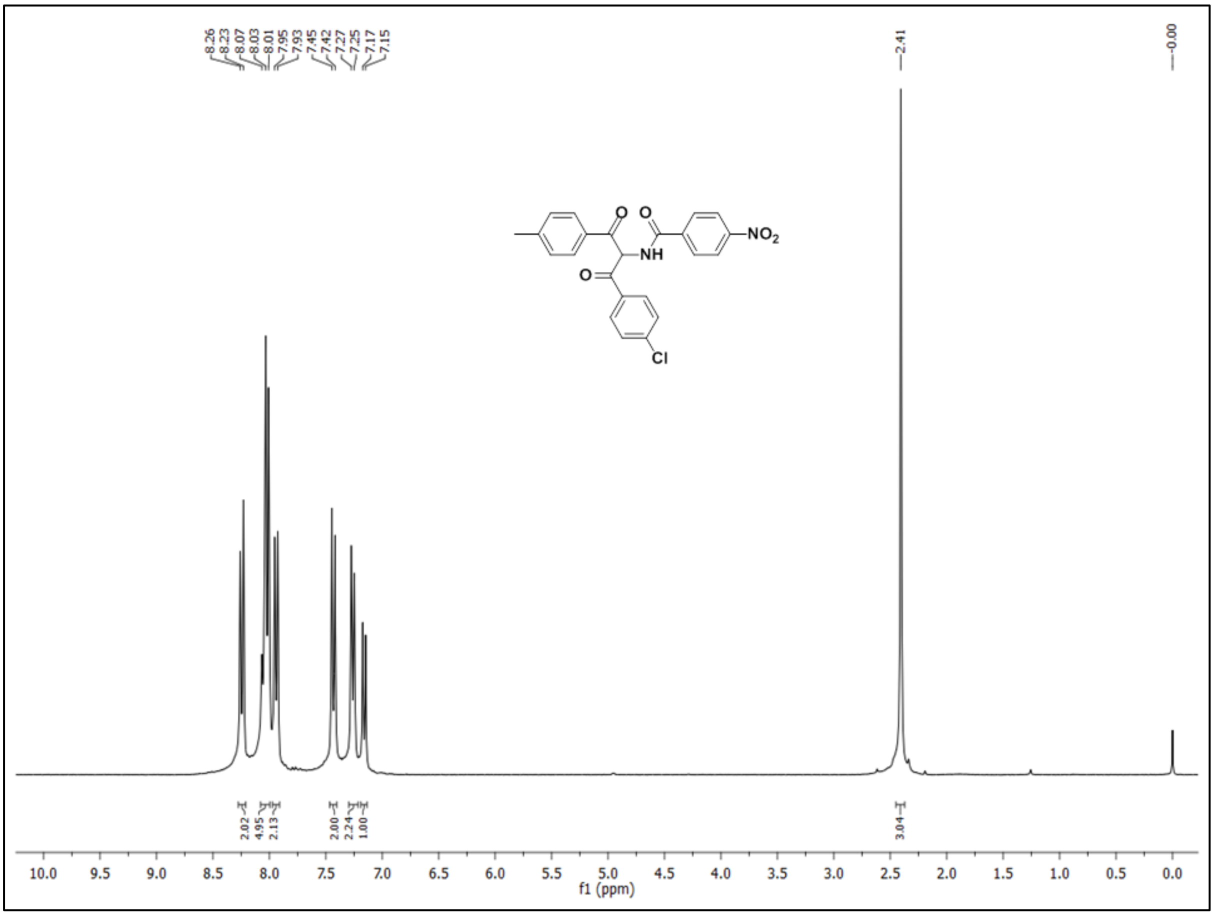

Fig. 37: ^{1}H NMR Spectrum of compound **3l**

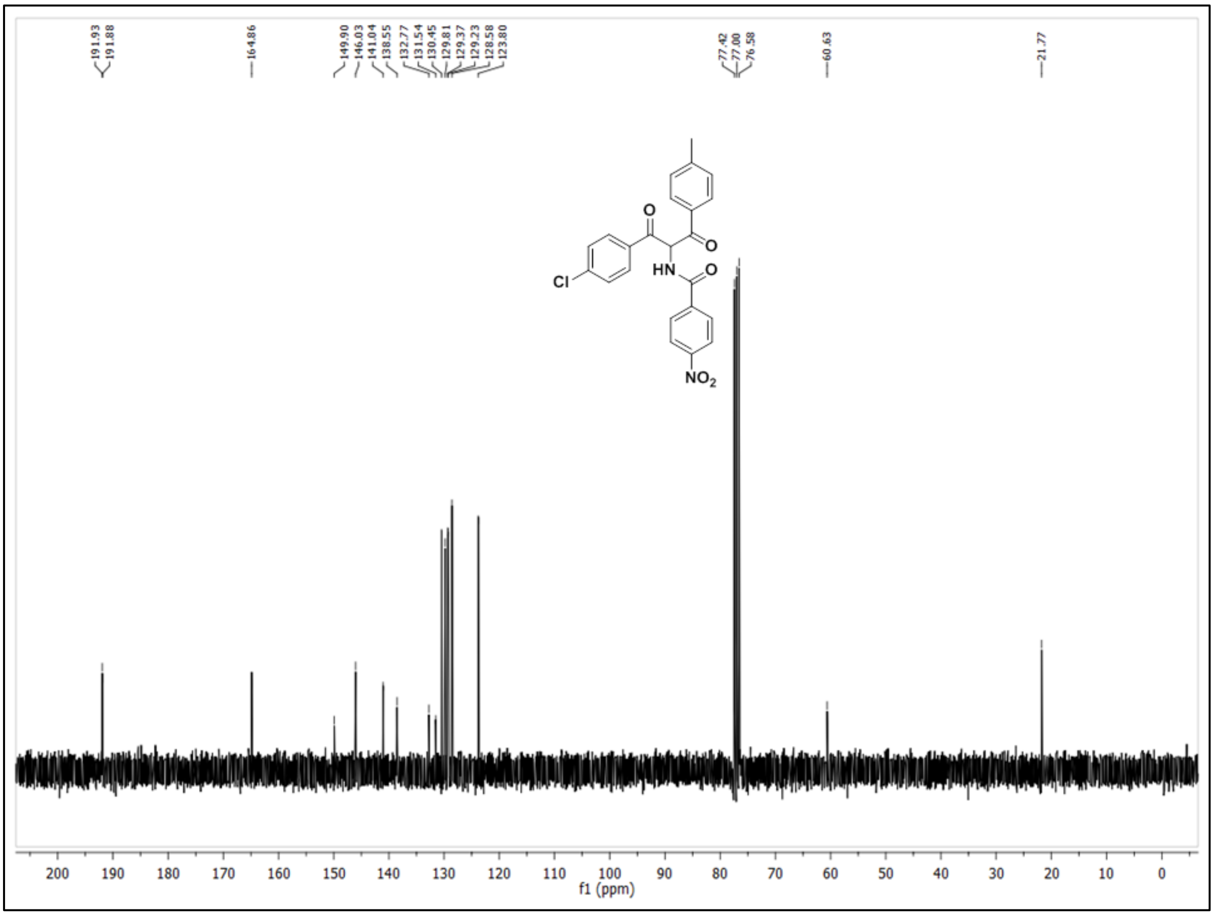

Fig. 38: ^{13}C NMR Spectrum of compound **3l**

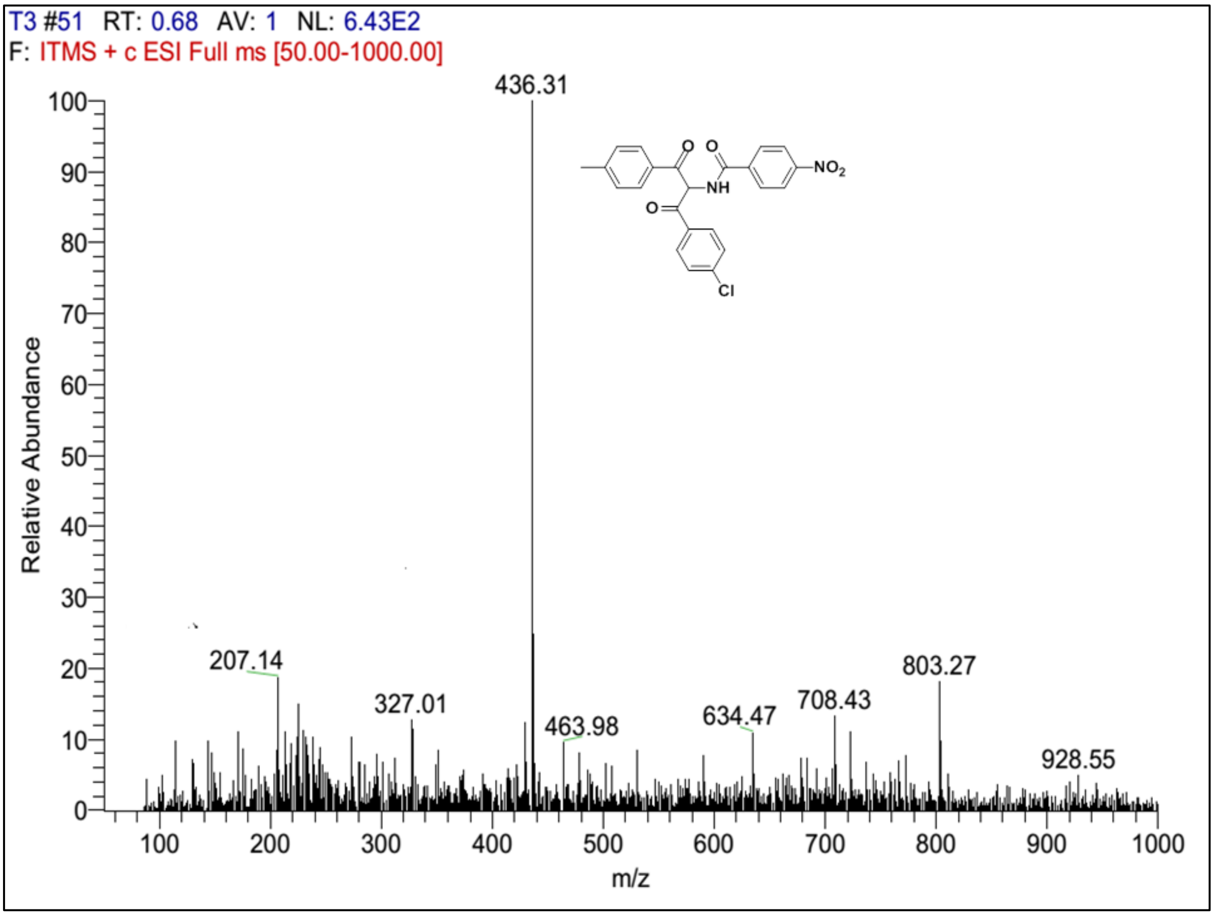

Fig. 39: Mass Spectrum of compound **3l**

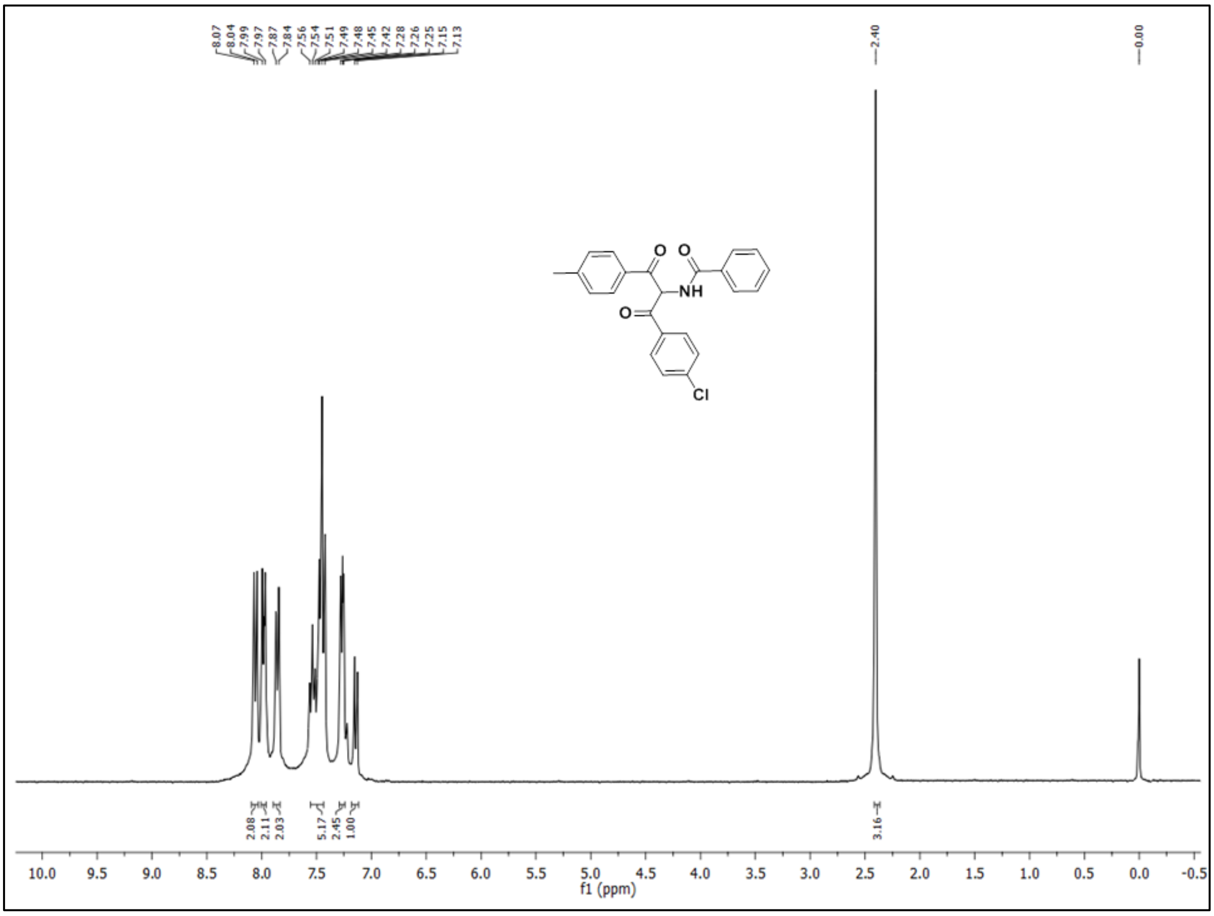

Fig. 40: ^{1}H NMR Spectrum of compound **3m**

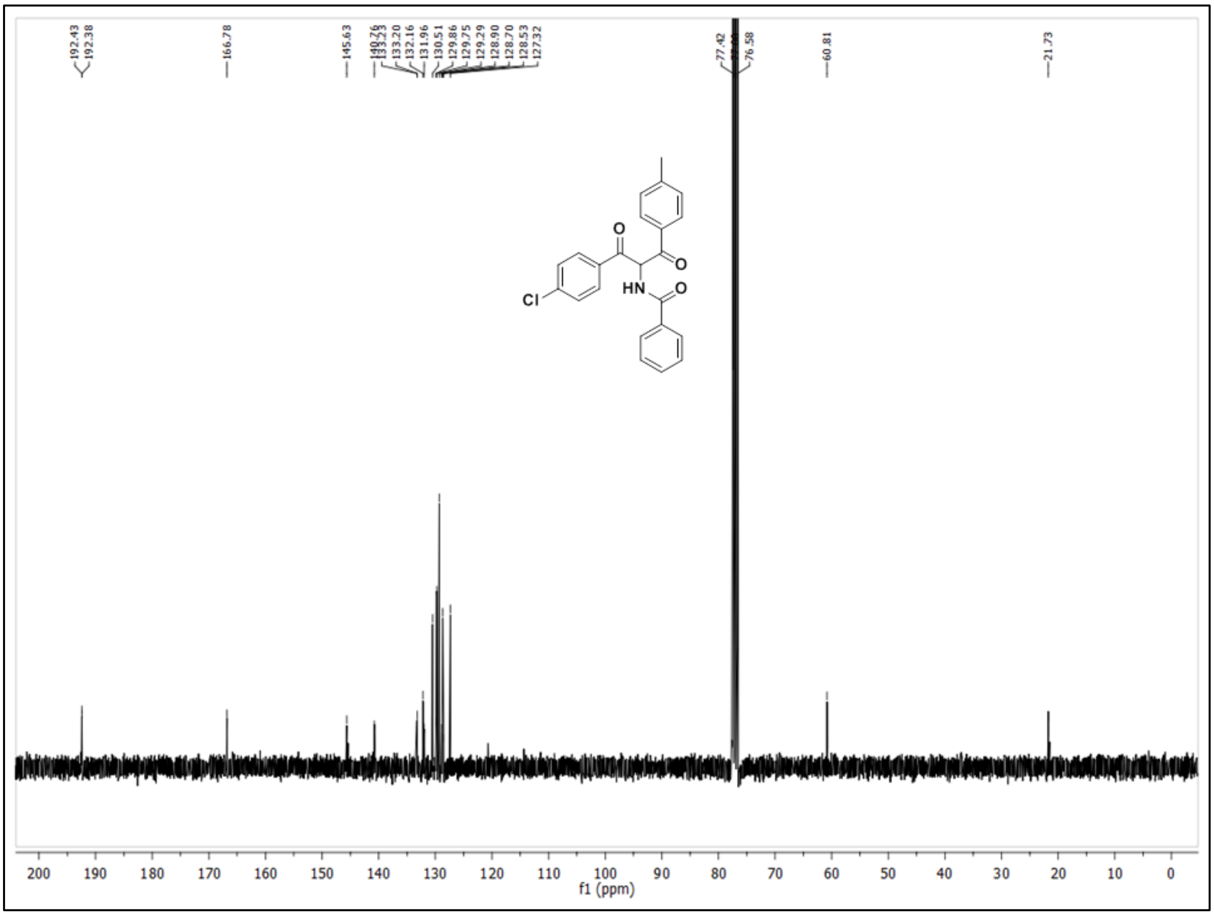

Fig. 41: ^{13}C NMR Spectrum of compound **3m**

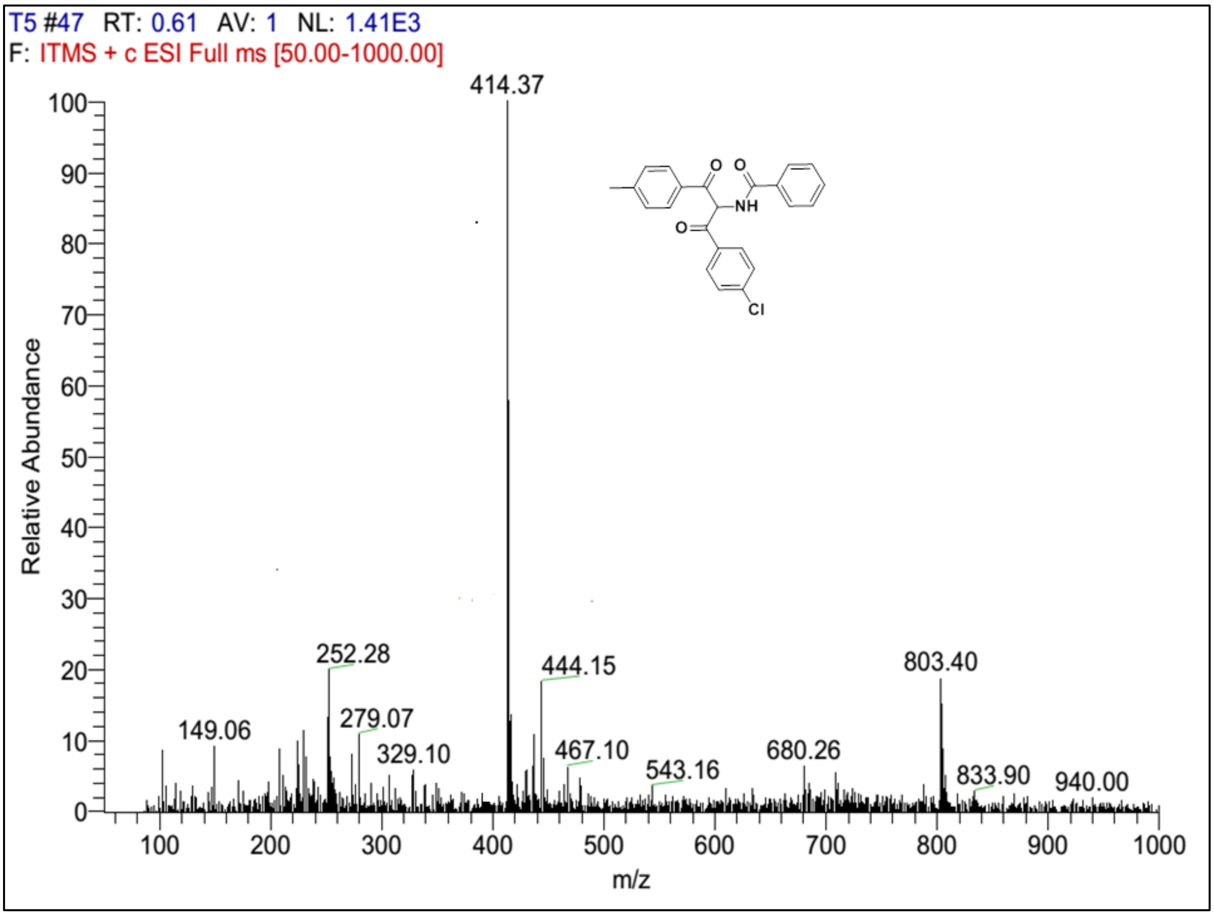

Fig.42 : Mass Spectrum of compound **3m**

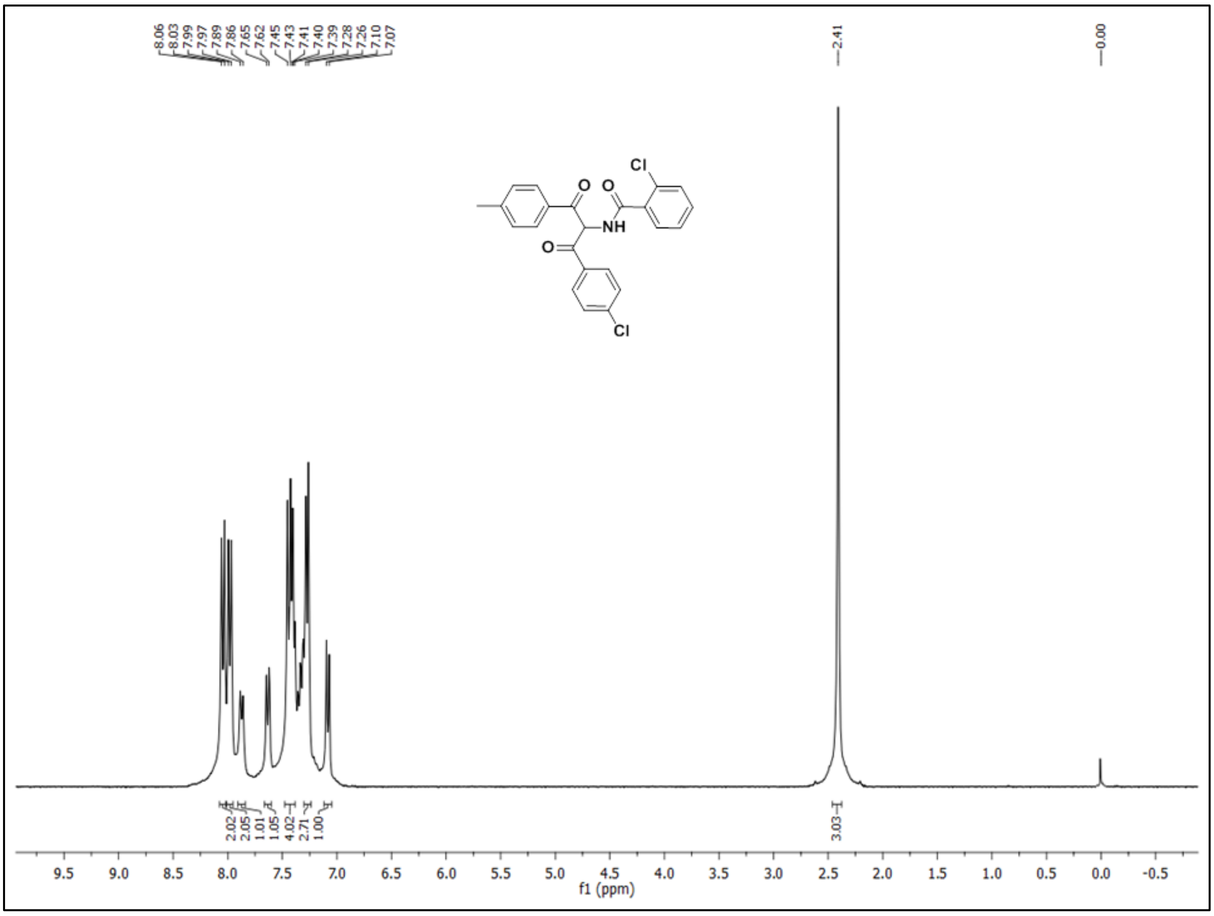

Fig. 43: ^{1}H NMR Spectrum of compound **3n**

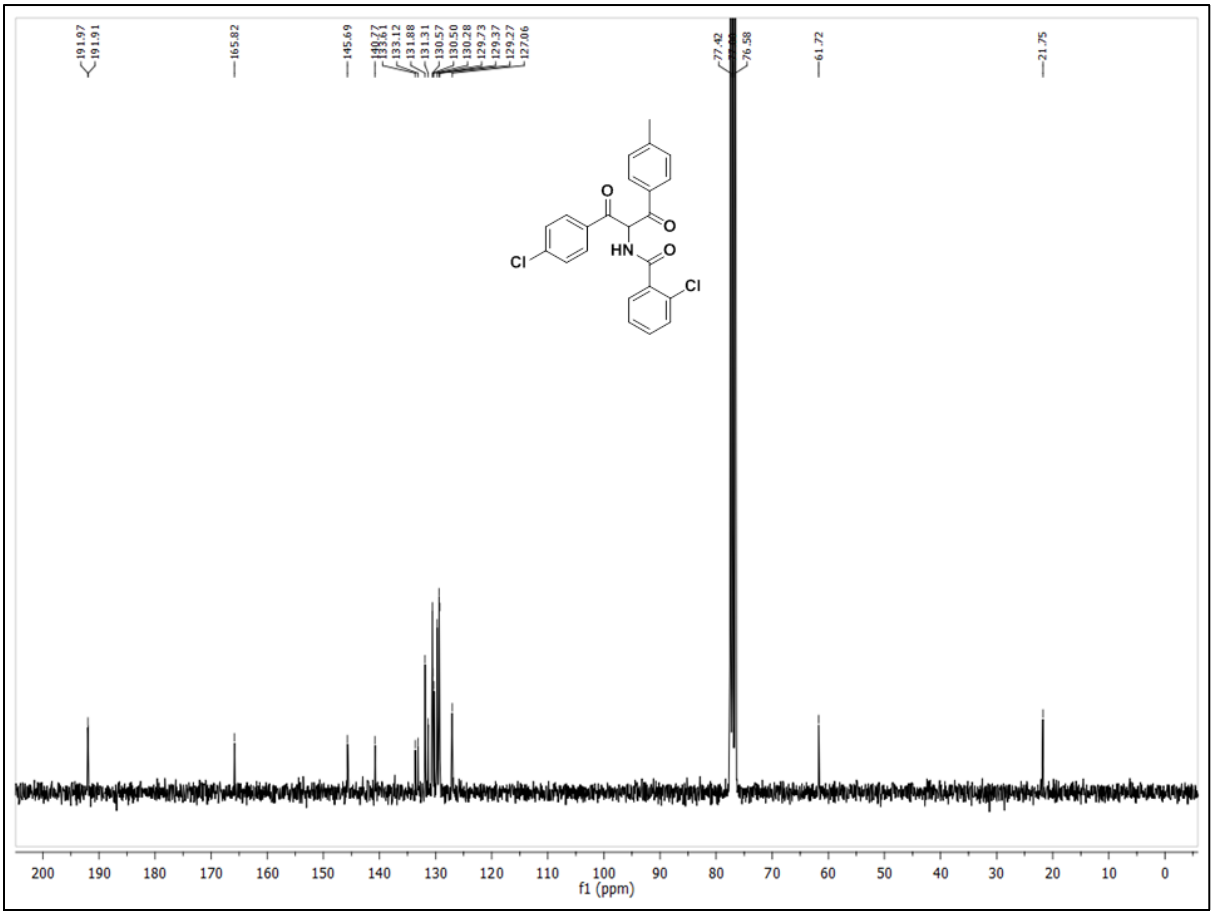

Fig. 44: ^{13}C NMR Spectrum of compound **3n**

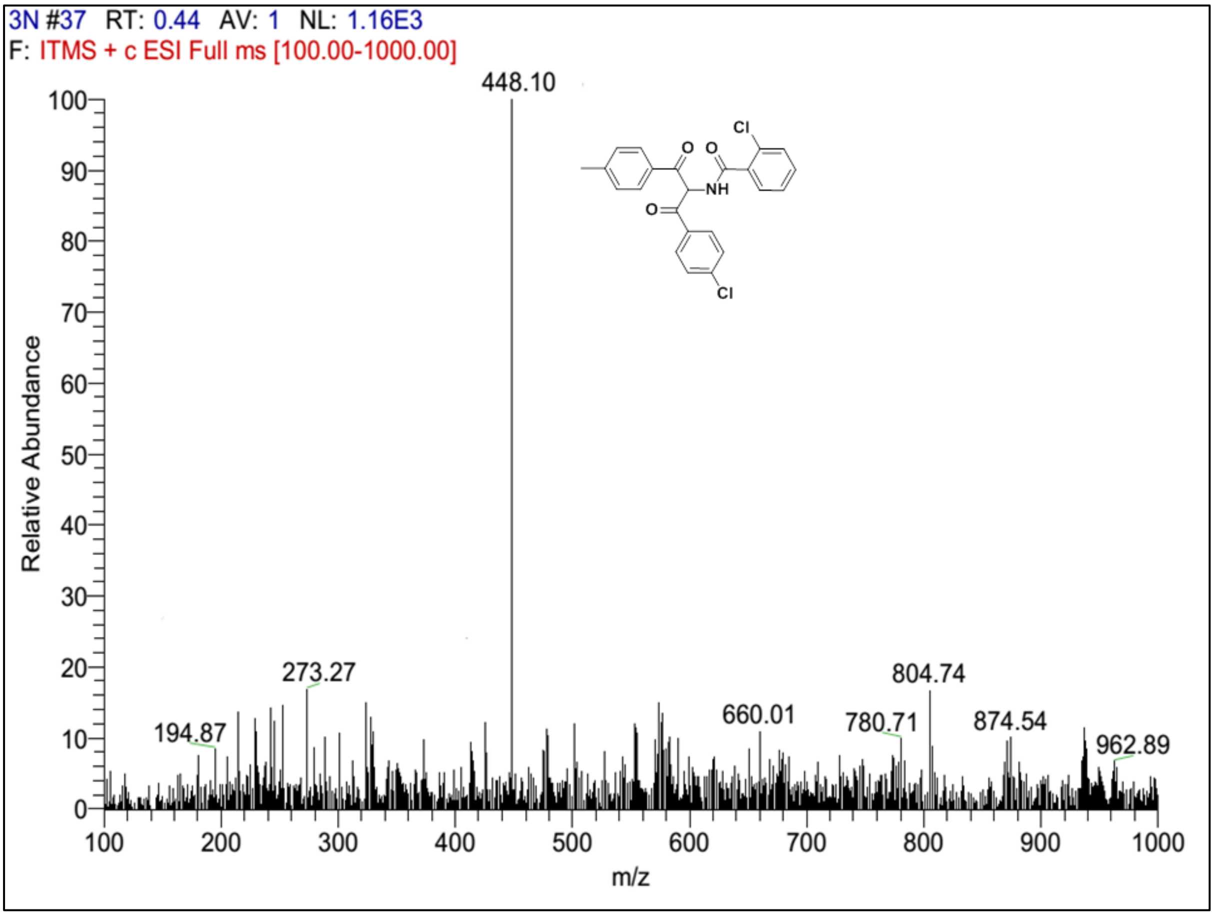

Fig. 45: Mass Spectrum of compound **3n**

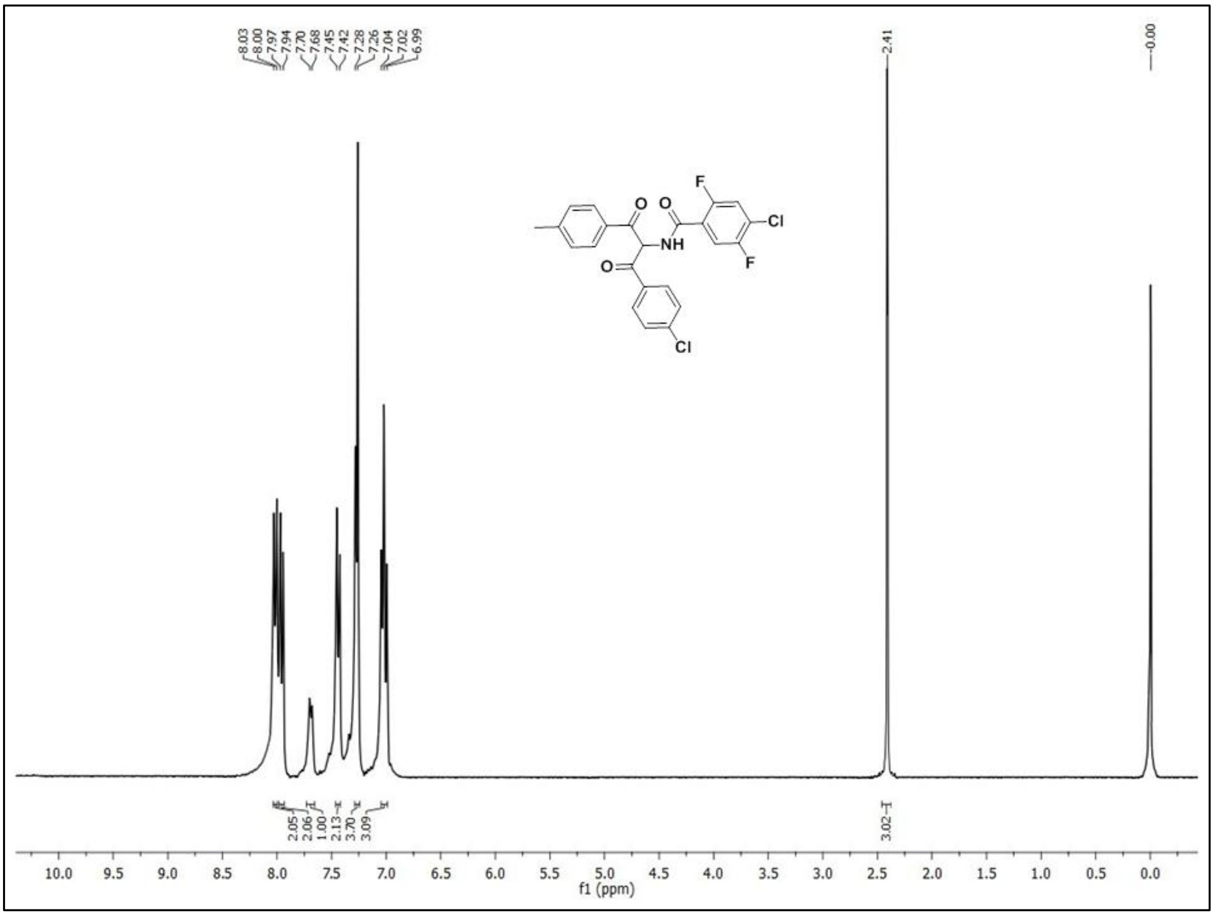

Fig. 46: ^{1}H NMR Spectrum of compound **3o**

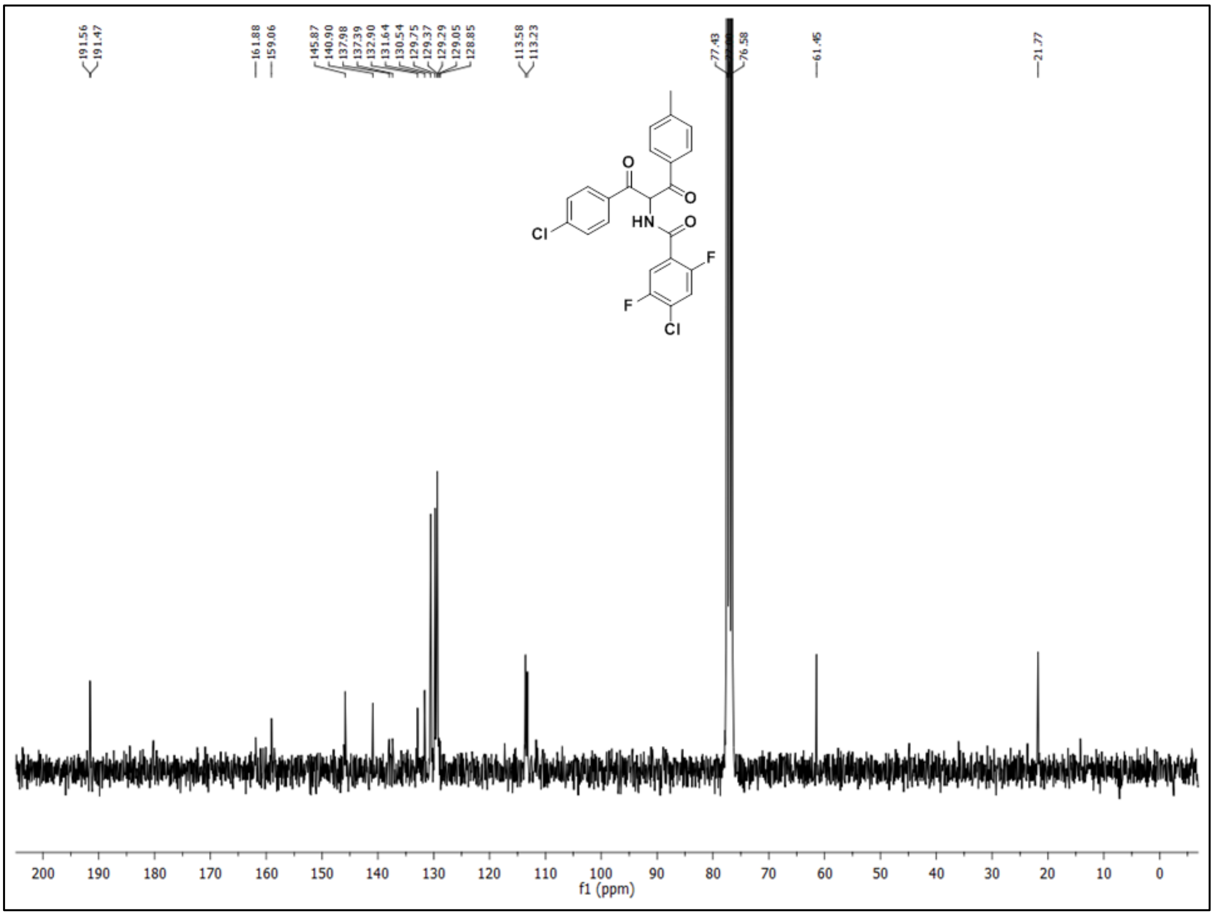

Fig. 47: ^{13}C NMR Spectrum of compound **3o**

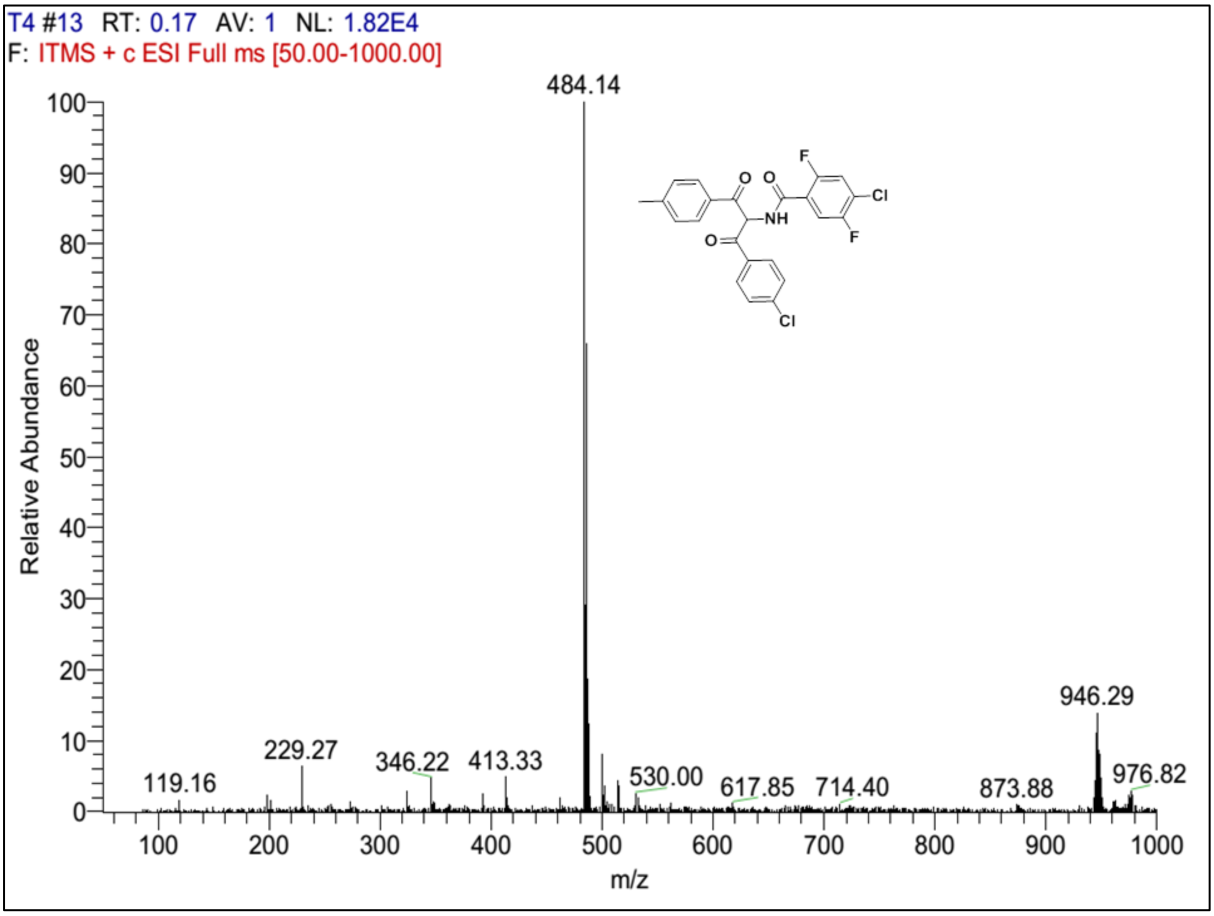

Fig. 48: Mass Spectrum of compound **3o**

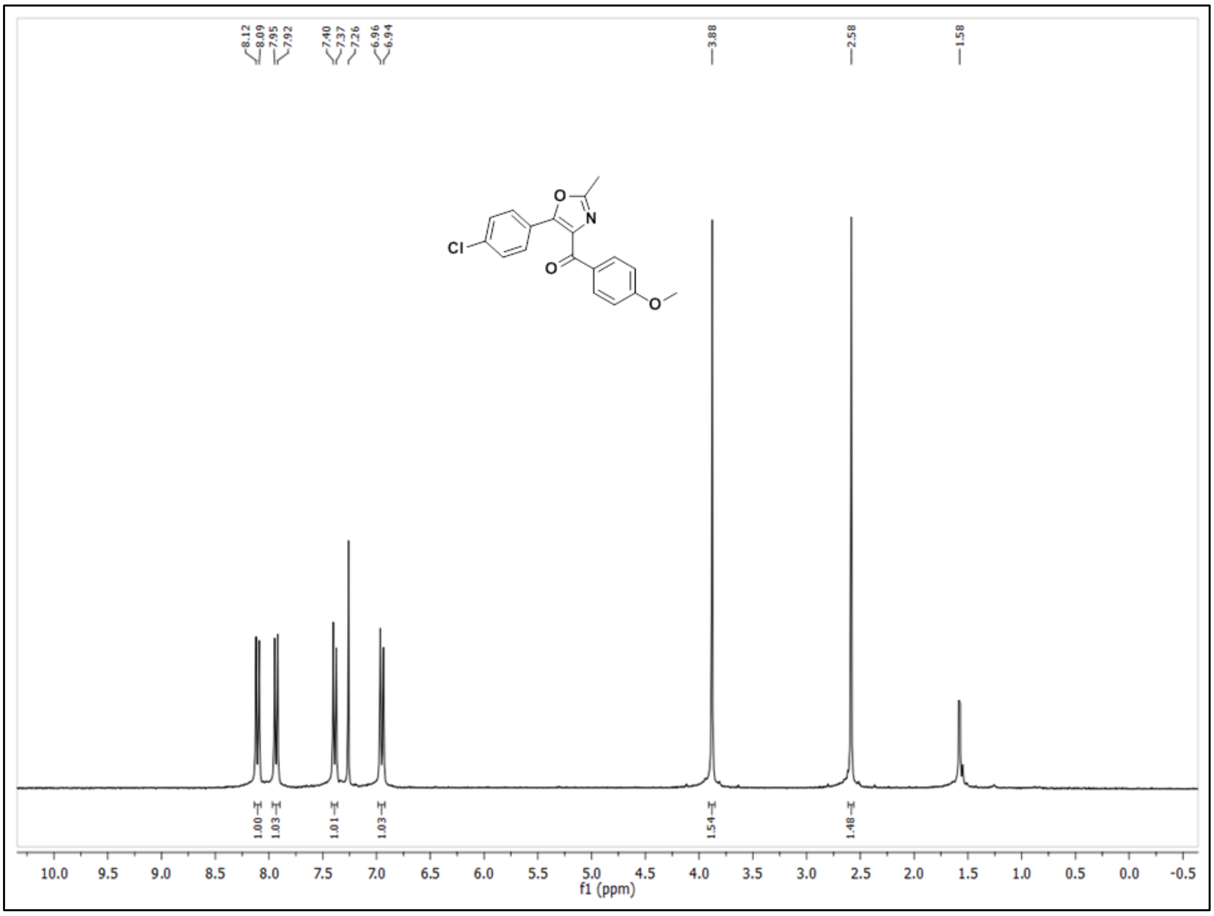

Fig. 49: ^{1}H NMR Spectrum of compound **7a**

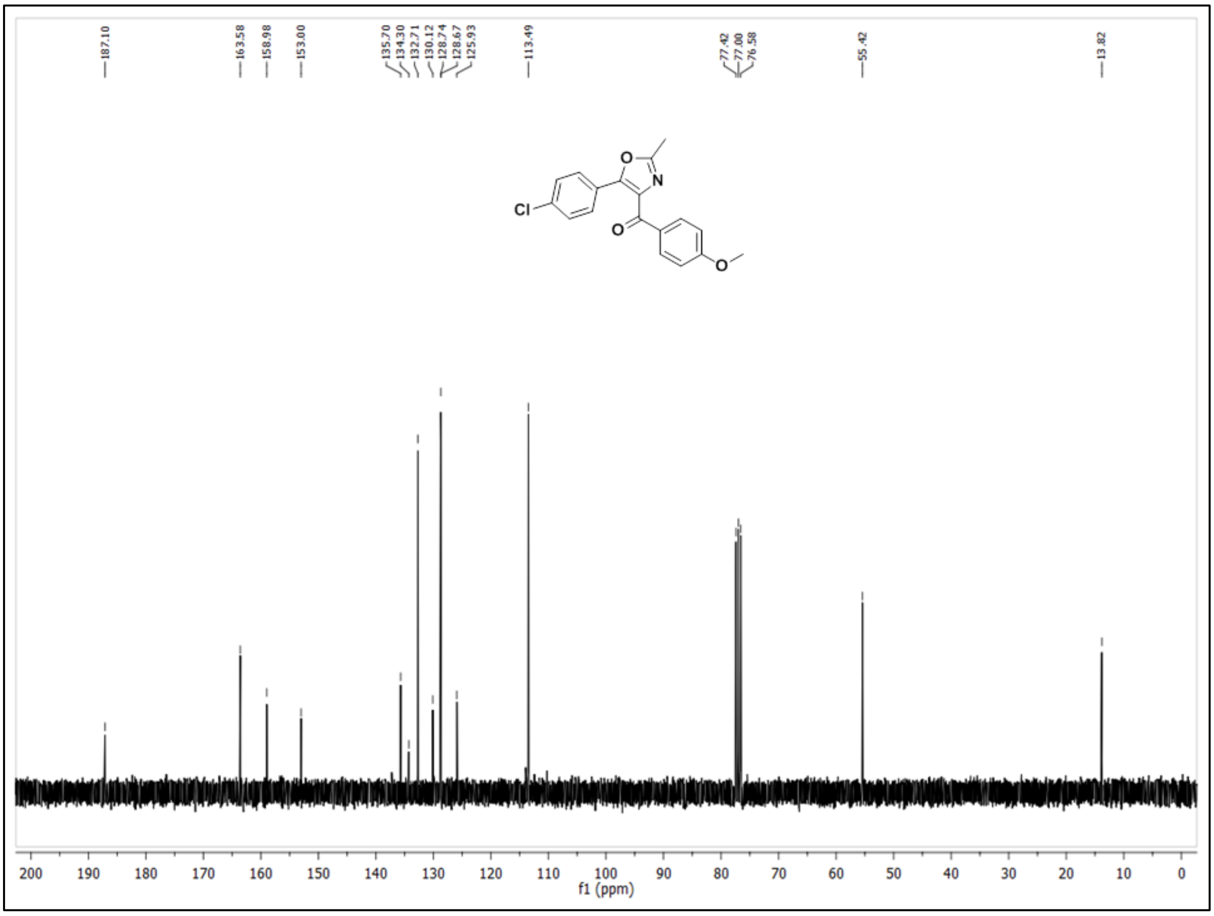

Fig. 50: ^{13}C NMR Spectrum of compound **7a**

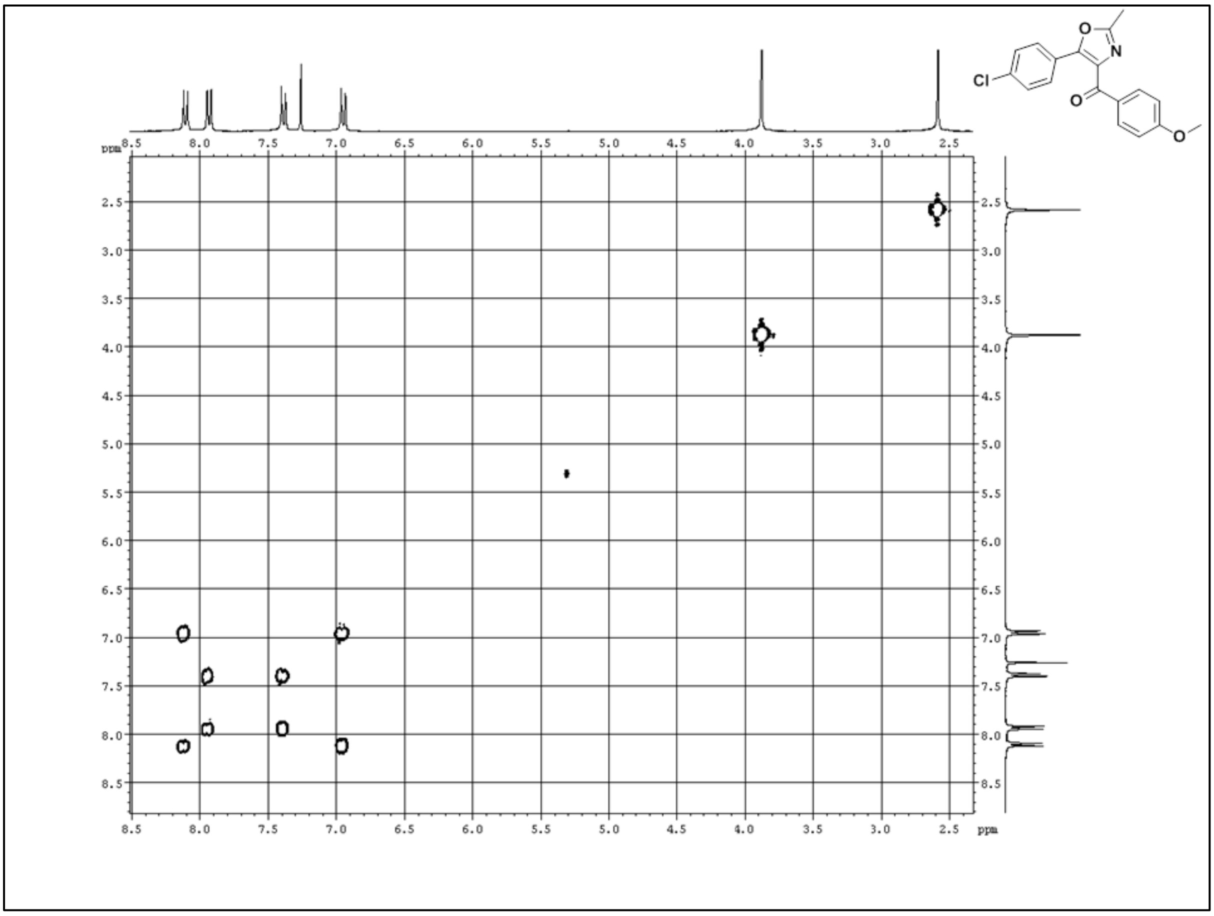

Fig. 51: HH COSY Spectrum of compound **7a**

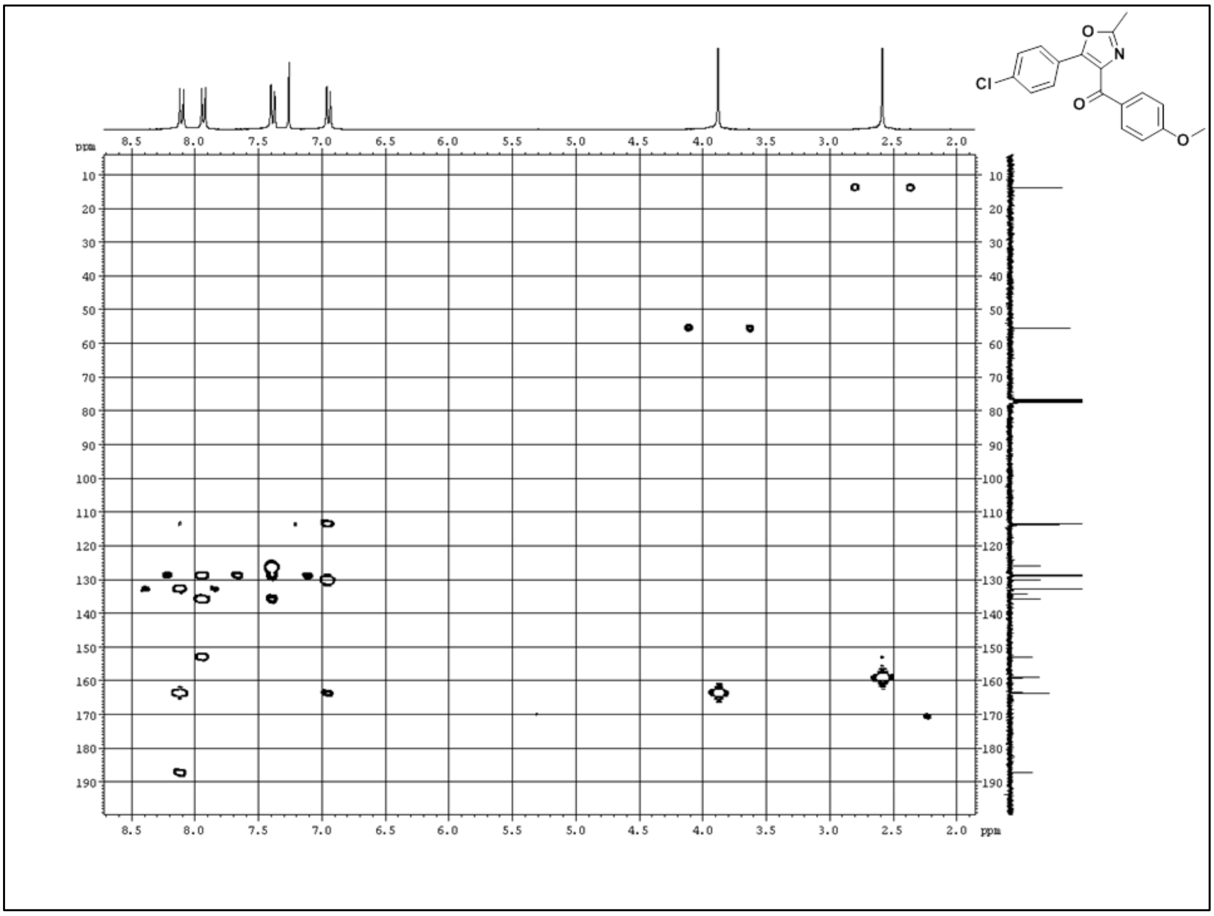

Fig. 52: HMBC Spectrum of compound **7a**

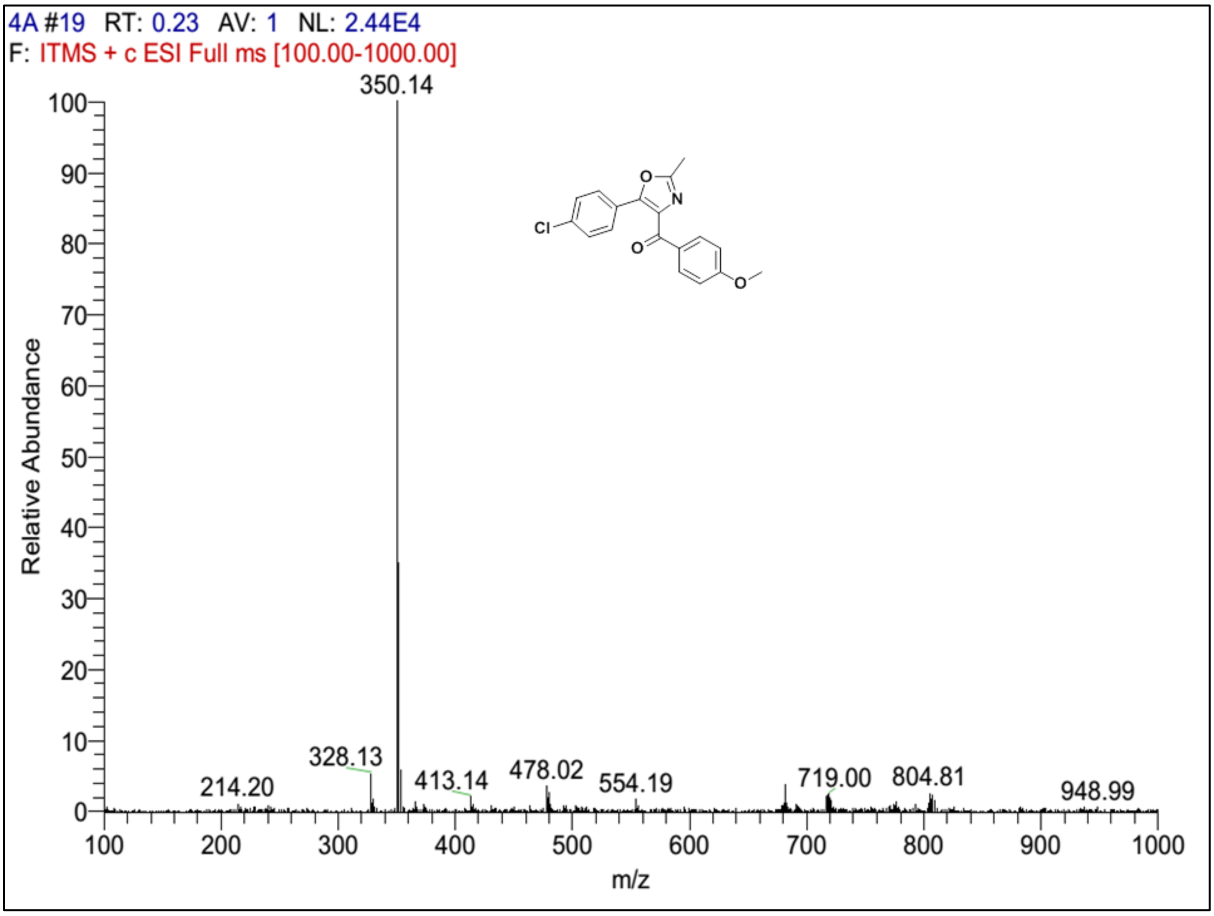

Fig. 53: Mass Spectrum of compound **7a**

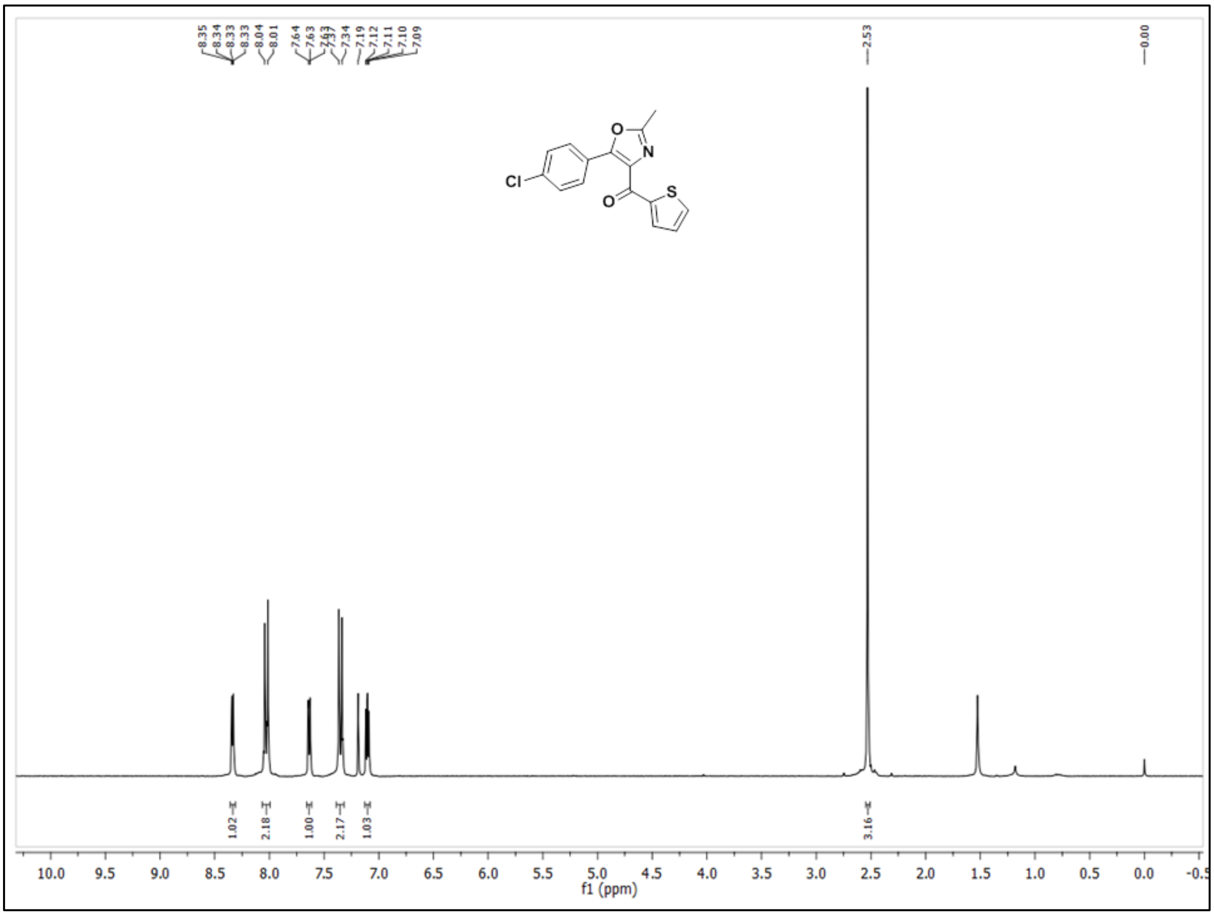

Fig. 54: ^{1}H NMR Spectrum of compound **7b**

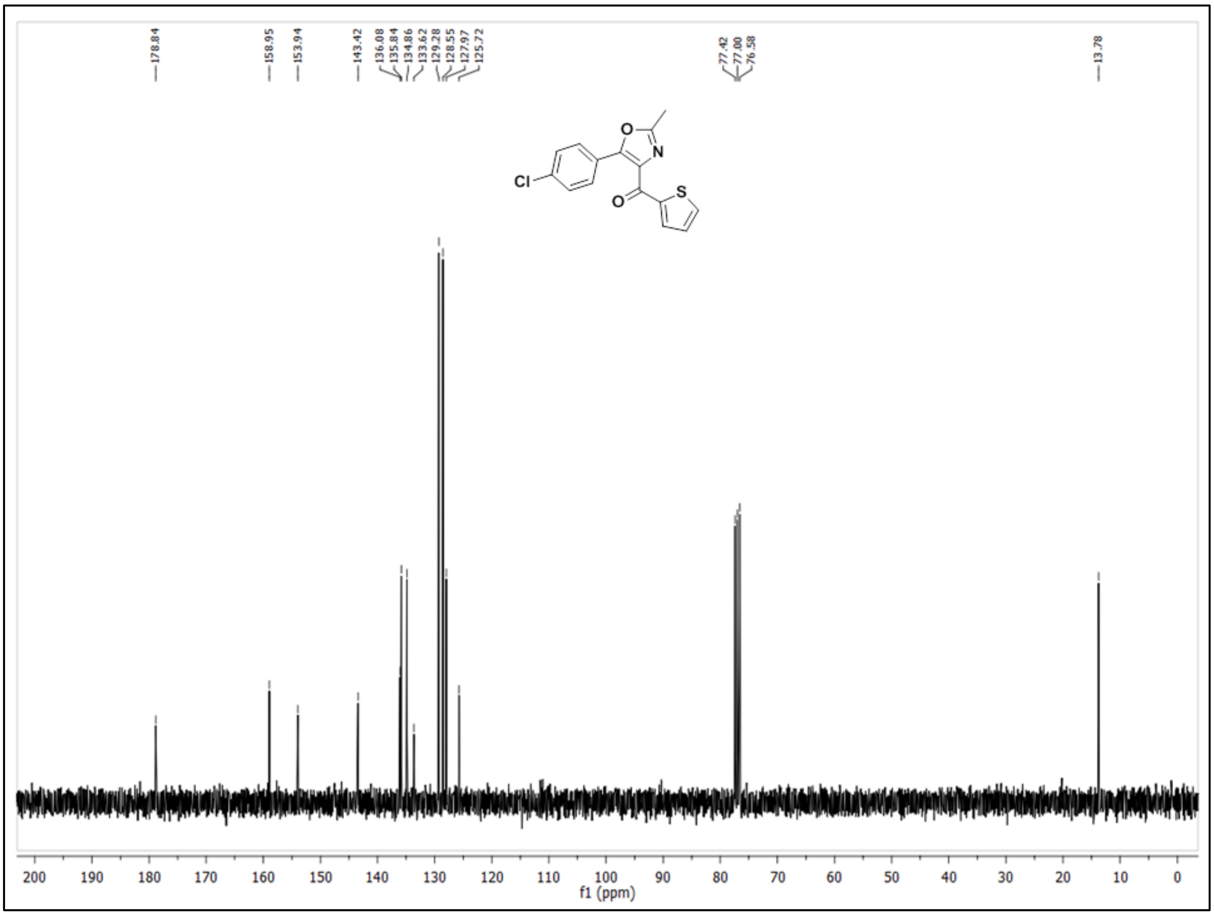

Fig. 55: ^{13}C NMR Spectrum of compound **7b**

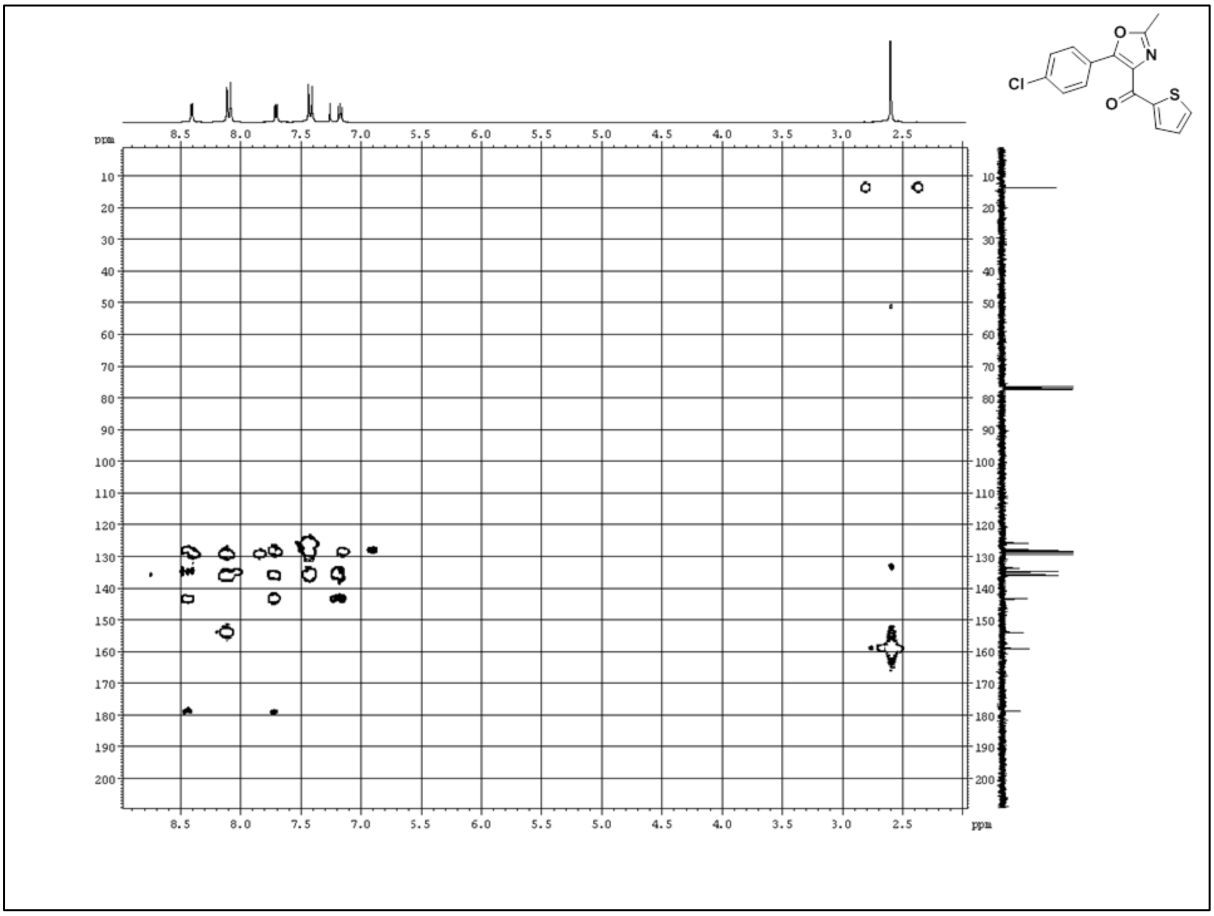

Fig. 56: HMBC Spectrum of compound **7b**

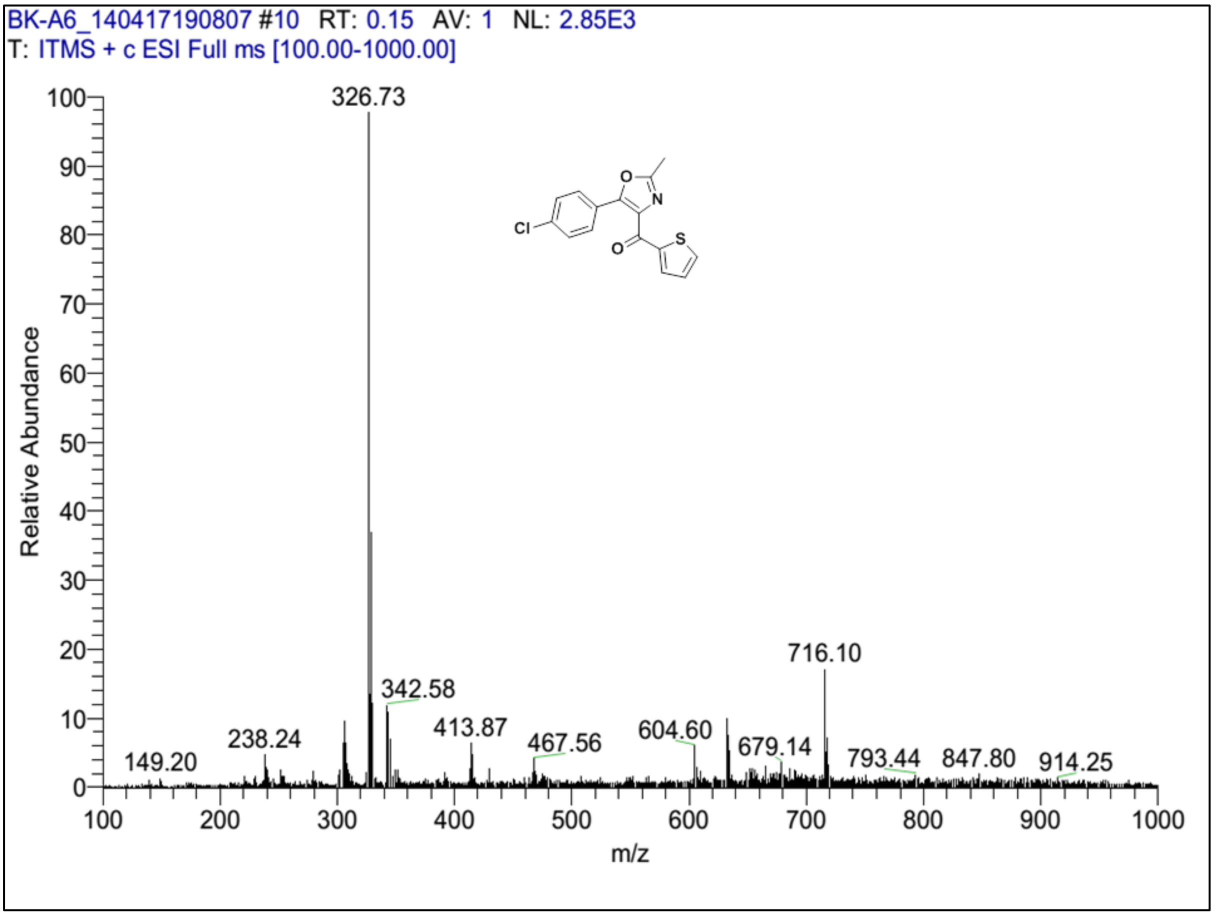

Fig. 57: Mass Spectrum of compound **7b**

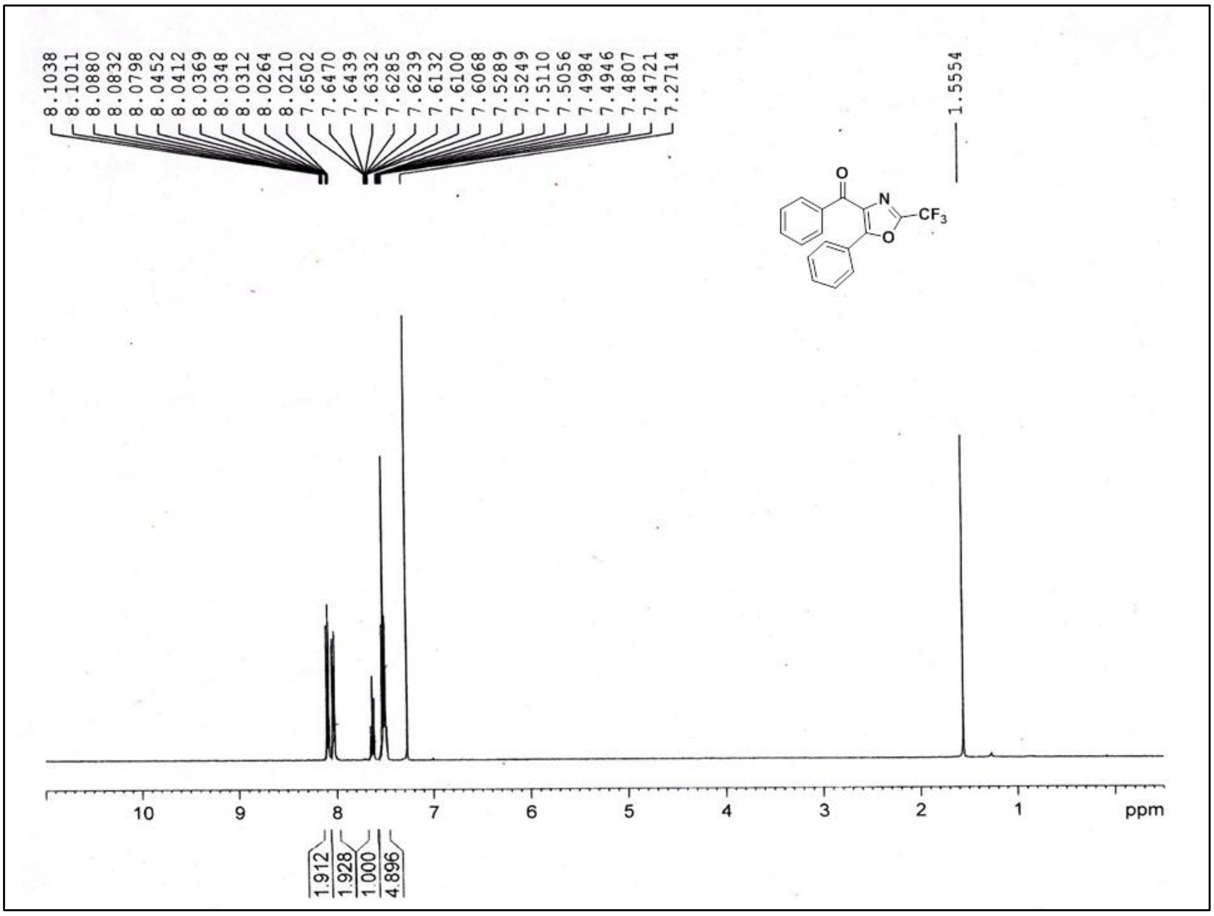

Fig. 58: ^{1}H NMR Spectrum of compound **8a**

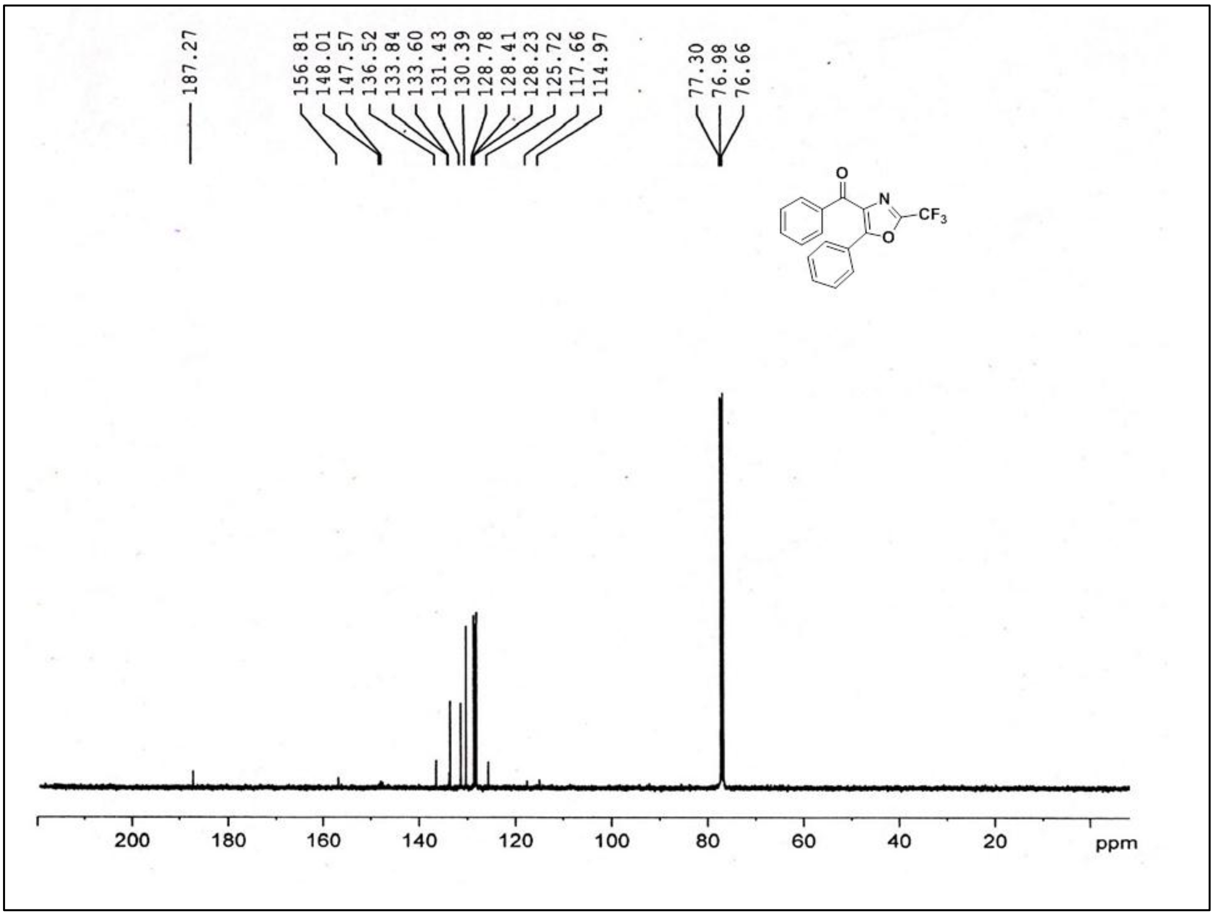

Fig. 59: ^{13}C NMR Spectrum of compound **8a**

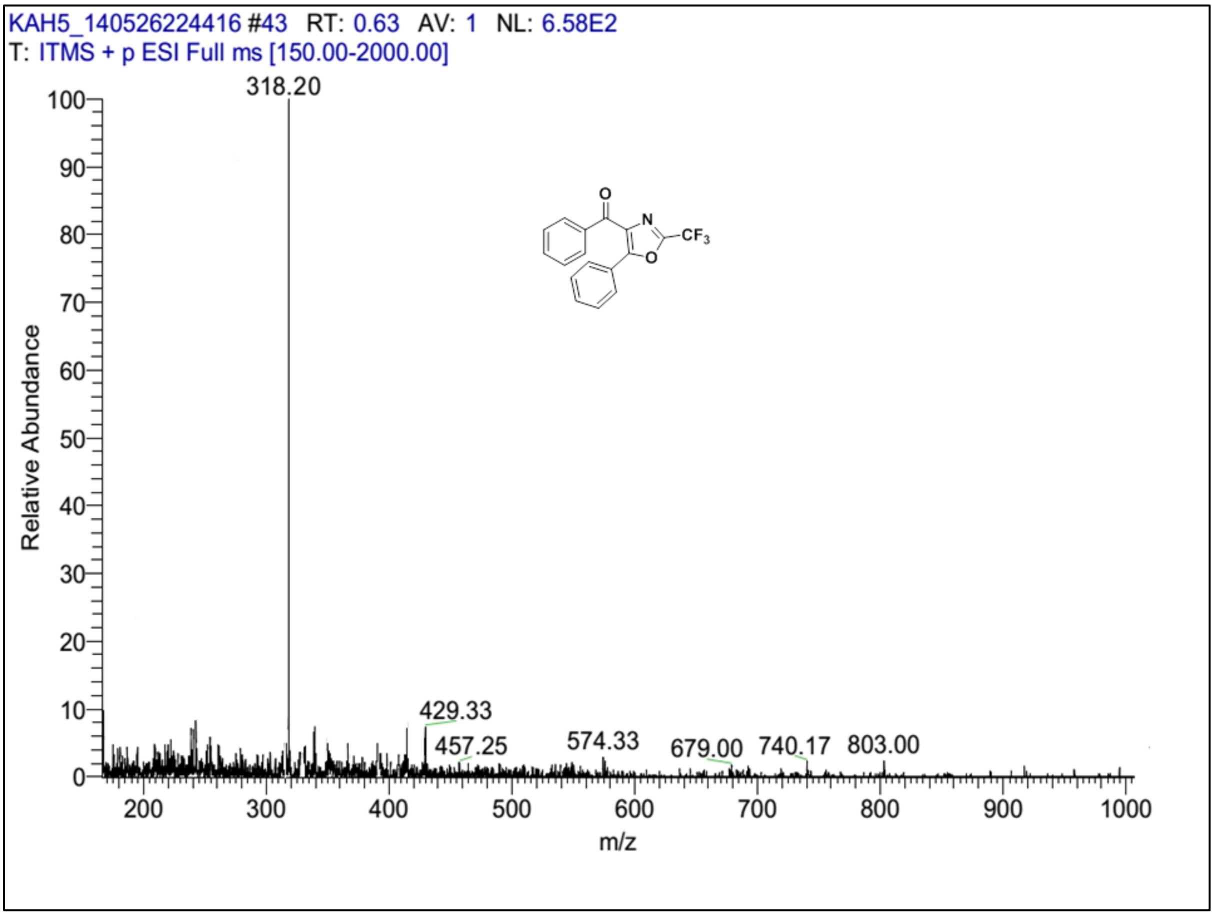

Fig. 60: Mass Spectrum of compound **8a**

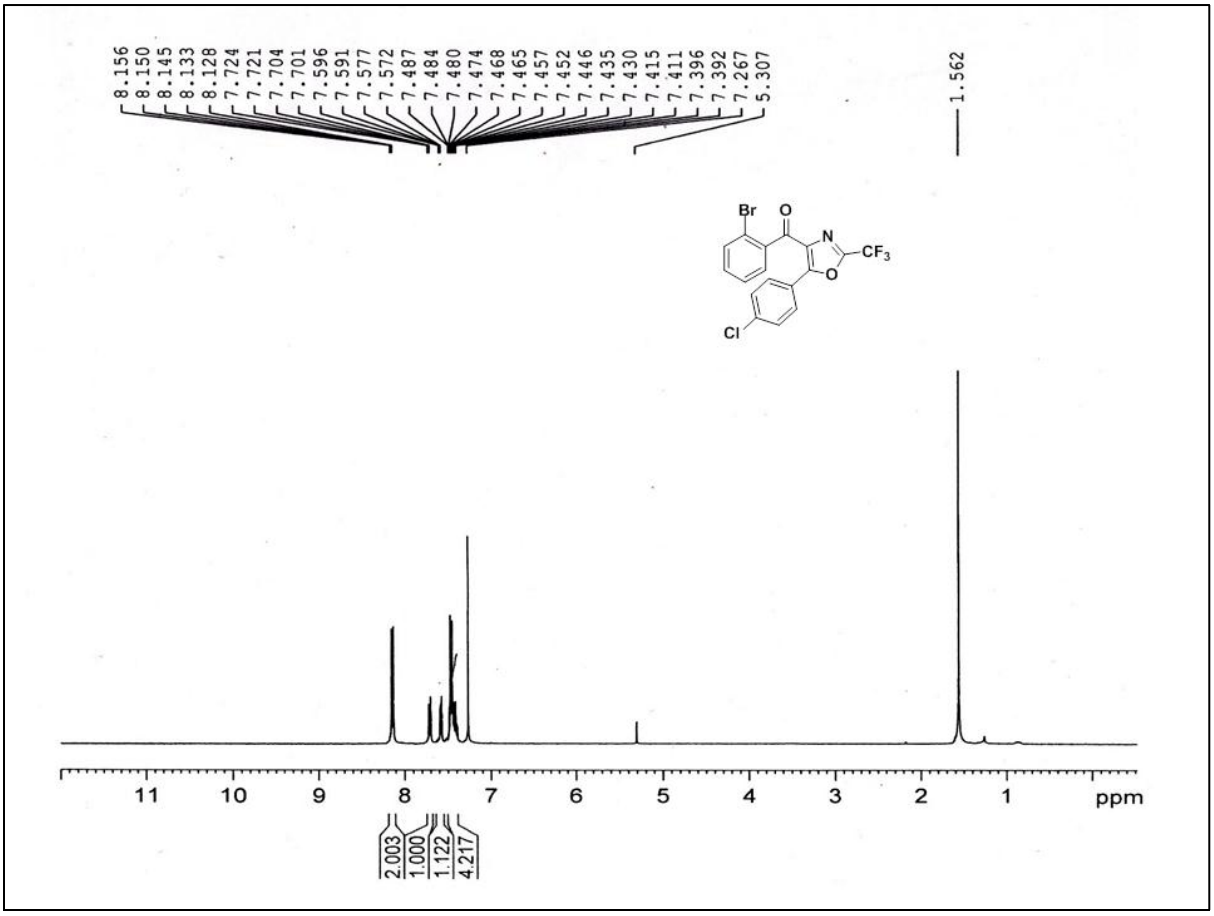

Fig. 61: ^{1}H NMR Spectrum of compound **8b**

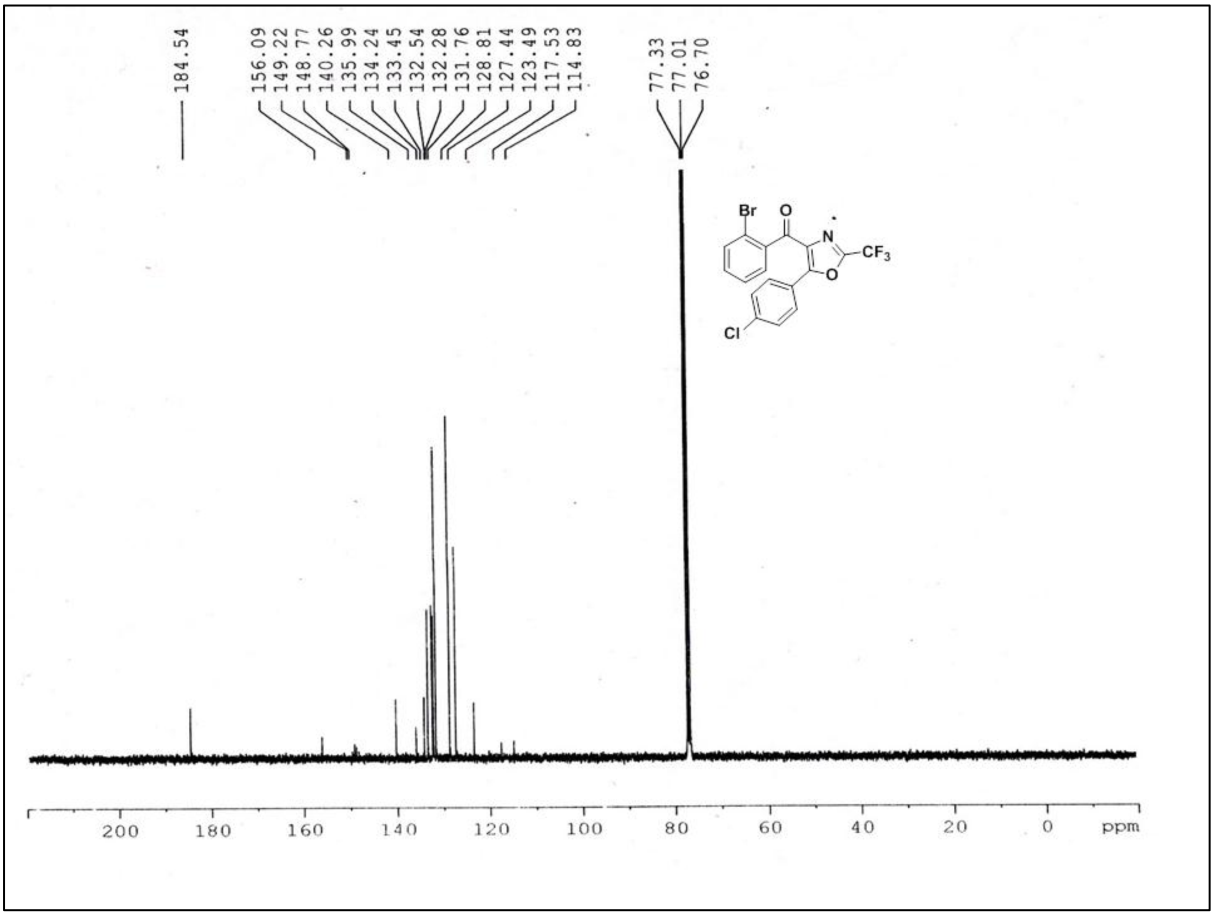

Fig. 62: ^{13}C NMR Spectrum of compound **8b**

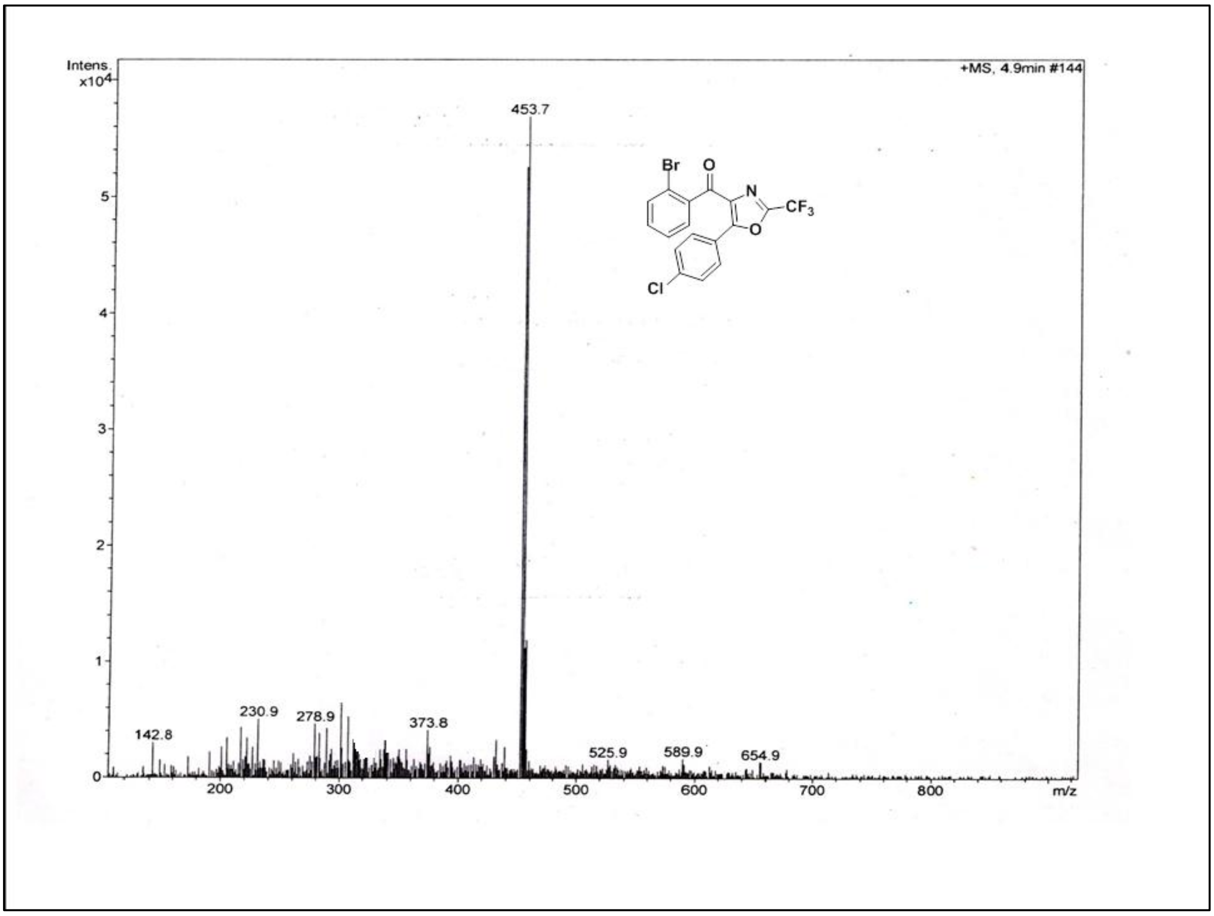

Fig. 63: Mass Spectrum of compound **8b**

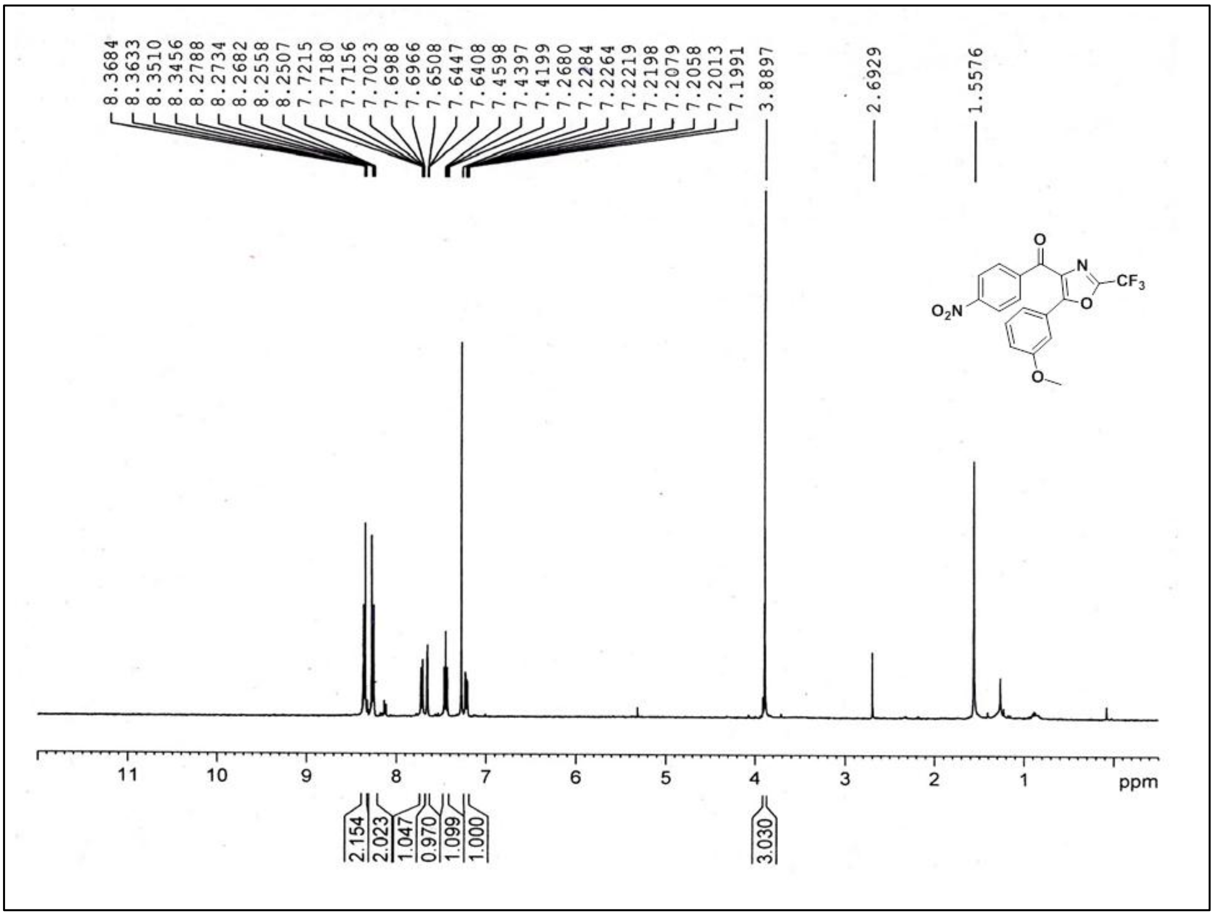

Fig. 64: ^{1}H NMR Spectrum of compound **8c**

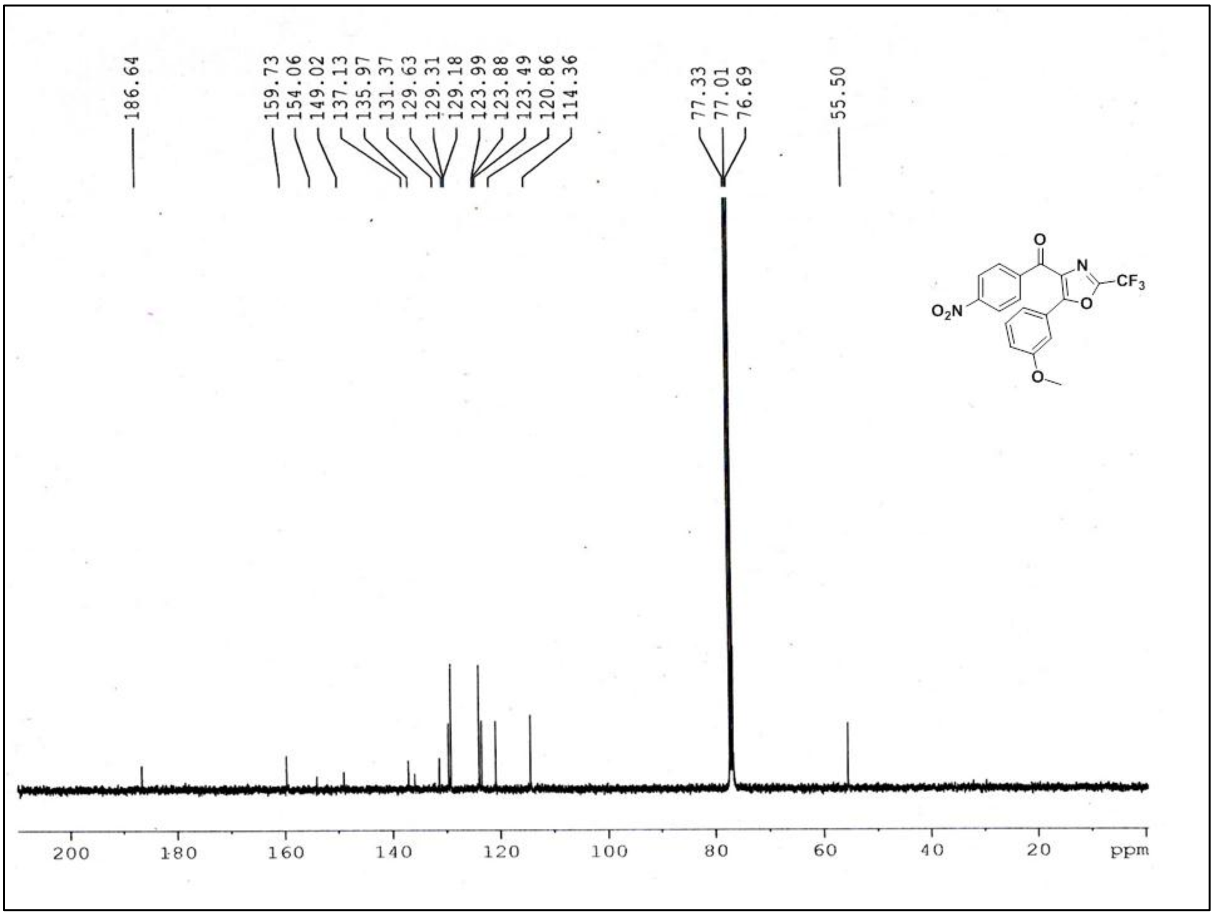

Fig. 65: ^{13}C NMR Spectrum of compound **8c**

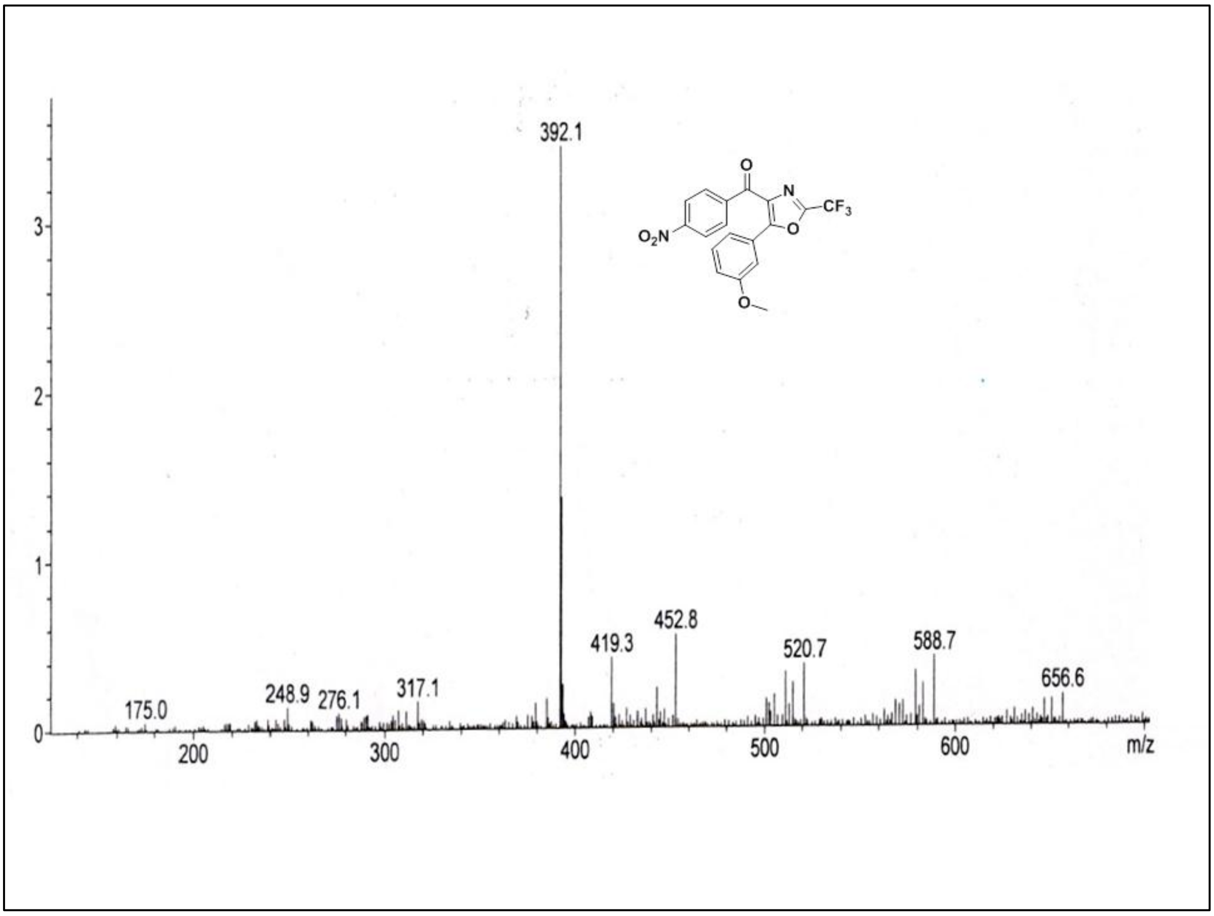

Fig. 66: Mass Spectrum of compound **8c**

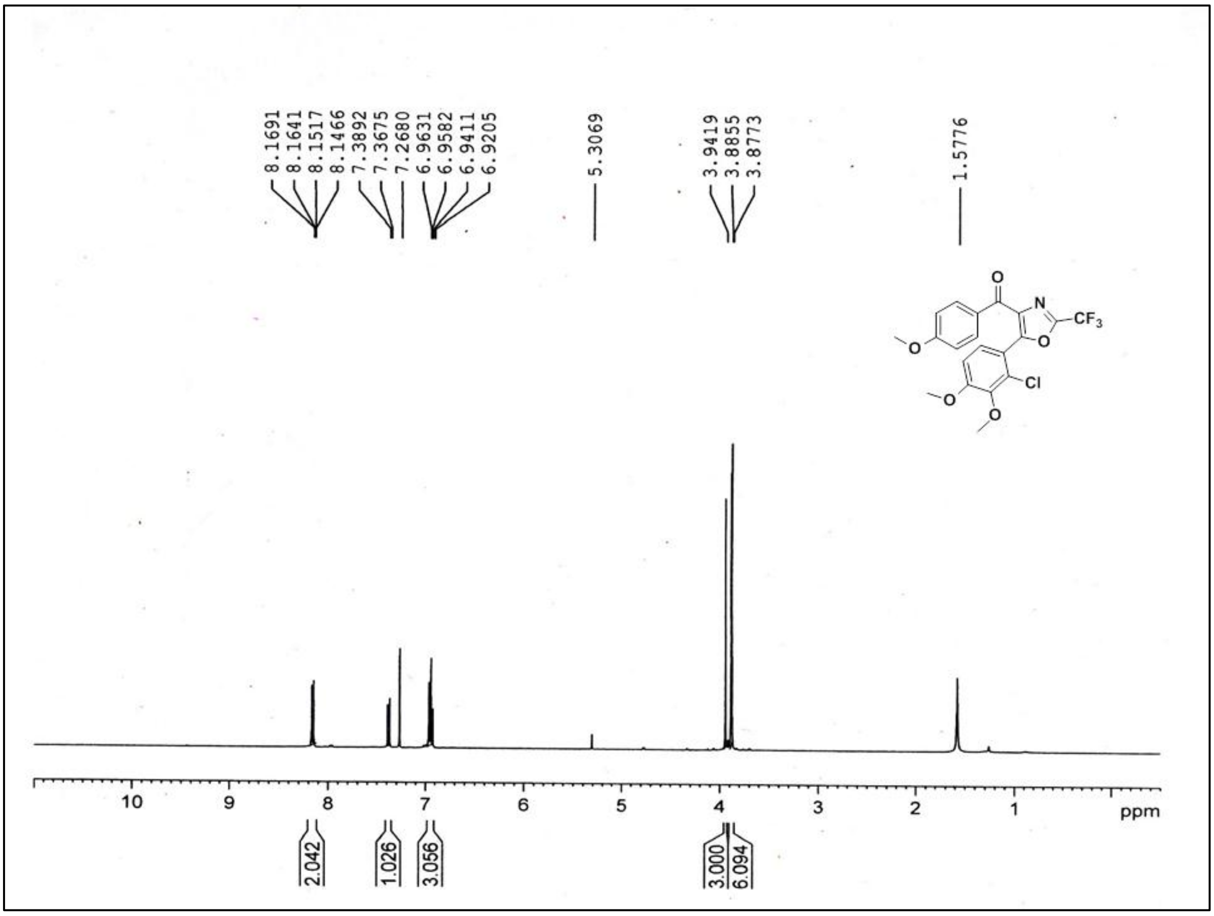

Fig. 67: ^{1}H NMR Spectrum of compound **8d**

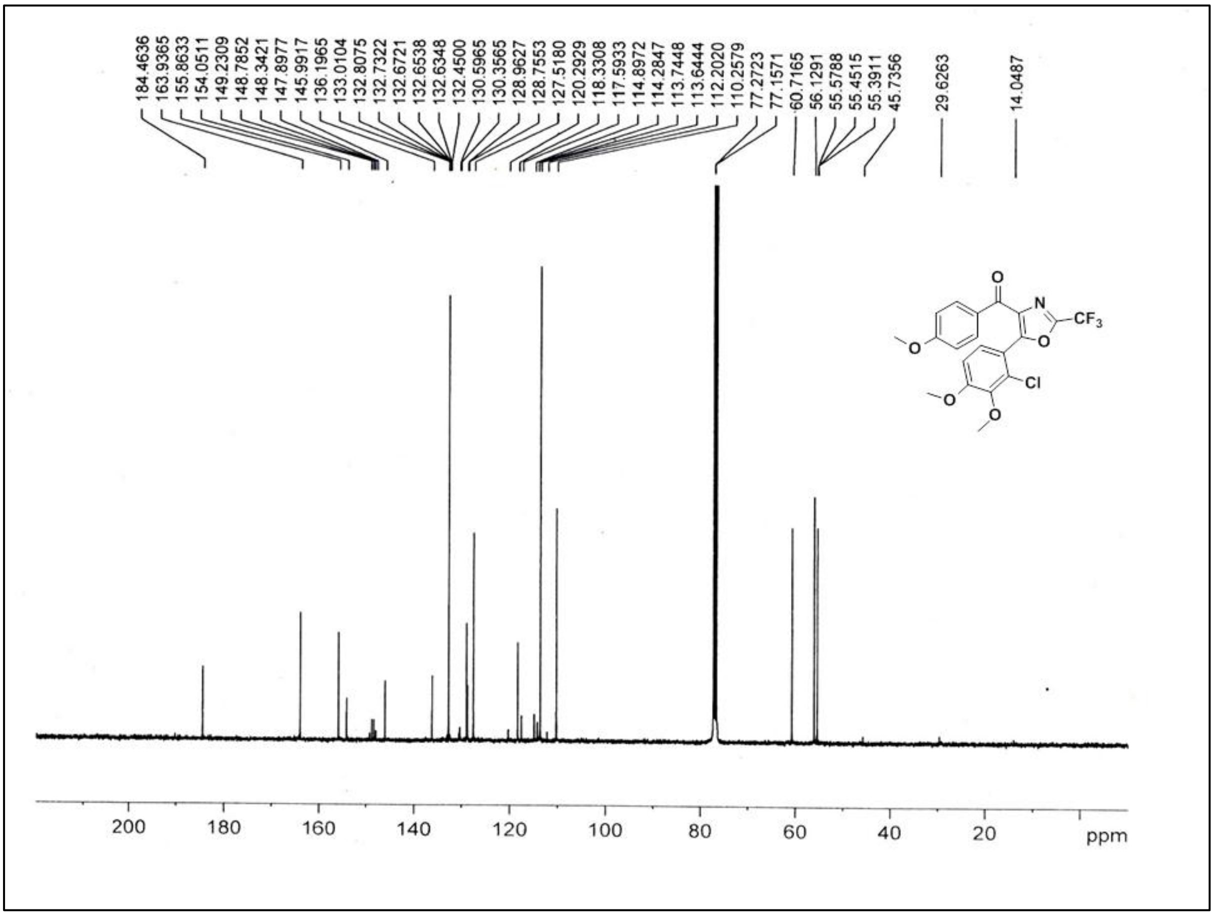

Fig. 68: ^{13}C NMR Spectrum of compound **8d**

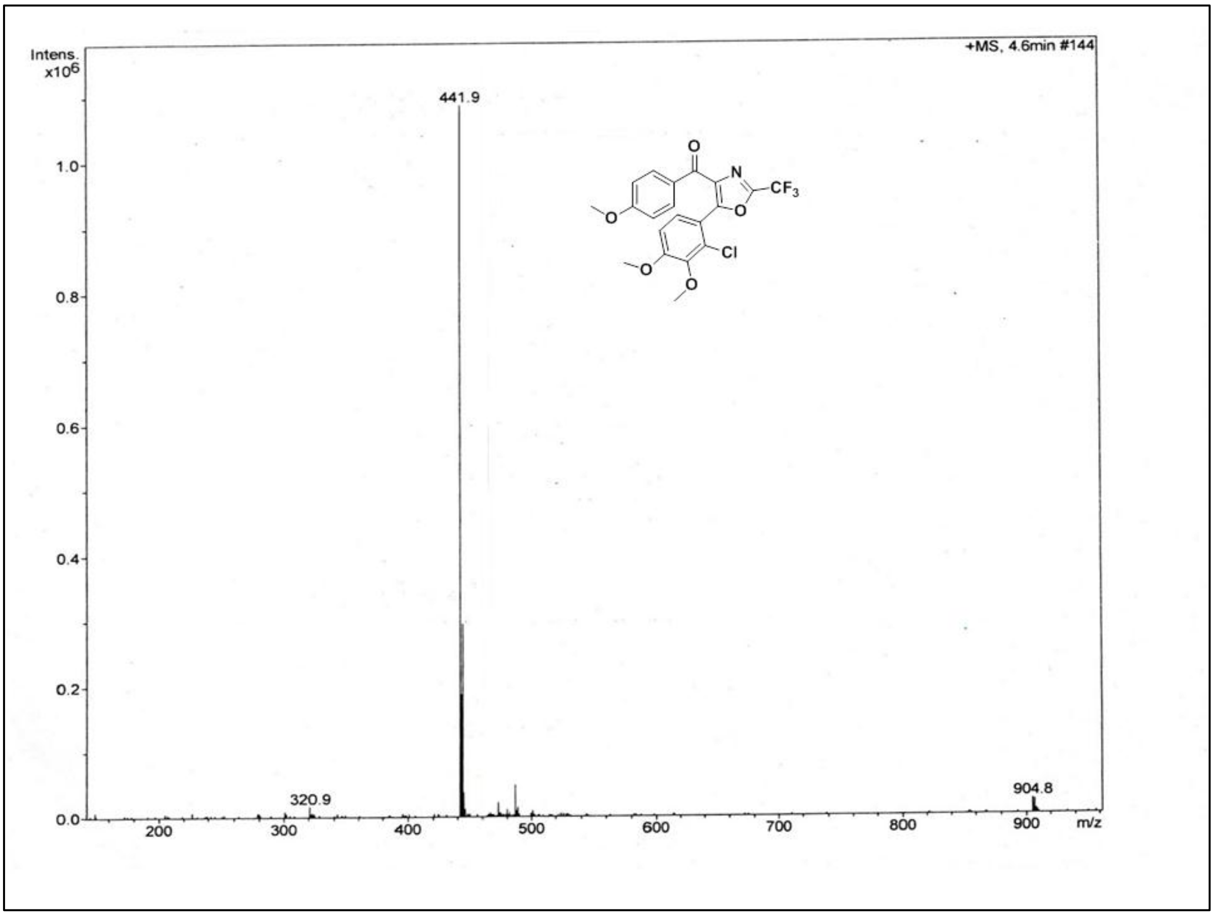

Fig. 69: Mass Spectrum of compound **8d**

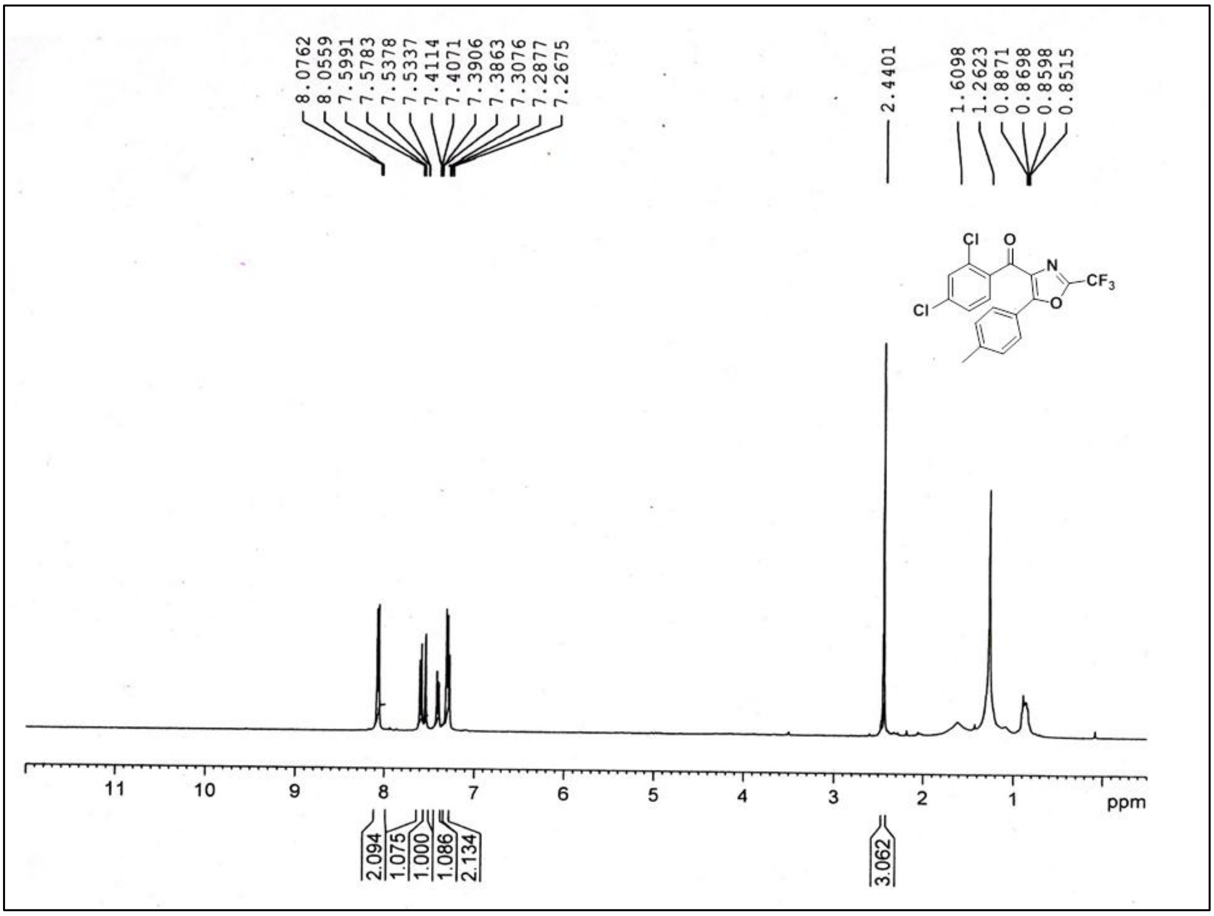

Fig. 70: ^{1}H NMR Spectrum of compound **8e**

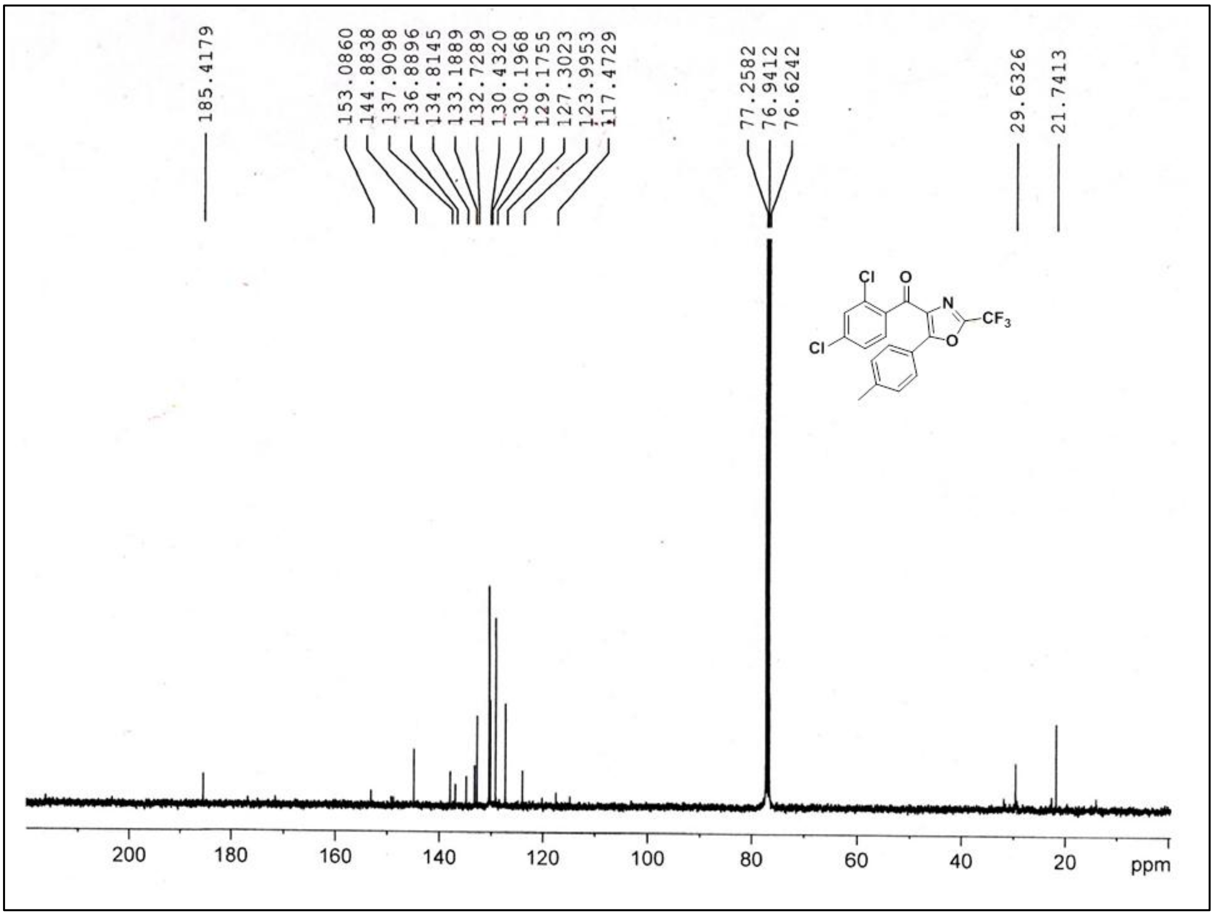

Fig. 71: ^{13}C NMR Spectrum of compound **8e**

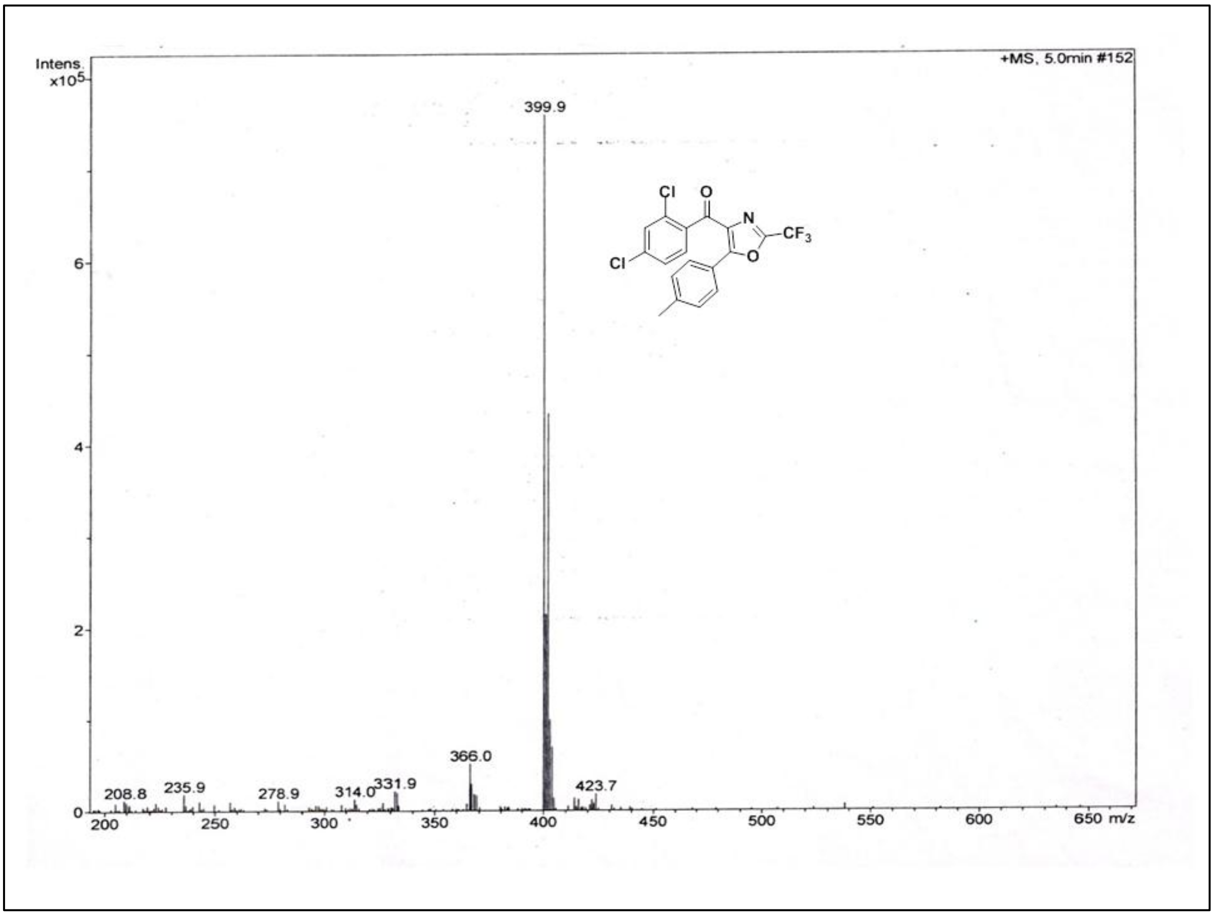

Fig.72: Mass Spectrum of compound **8e**
